# Genome-wide transcriptome and functional analysis of two contrasting genotypes reveals key genes for cadmium tolerance in barley

**DOI:** 10.1186/1471-2164-15-611

**Published:** 2014-07-19

**Authors:** Fangbin Cao, Fei Chen, Hongyan Sun, Guoping Zhang, Zhong-Hua Chen, Feibo Wu

**Affiliations:** College of Agriculture and Biotechnology, Zijingang Campus, Zhejiang University, Hangzhou, 310058 P.R. China; School of Science and Health, Hawkesbury Campus, University of Western Sydney, Locked Bag 1797, Penrith, NSW 2751 Australia

**Keywords:** ATPase, Cadmium fluorescent indicator, Cadmium tolerance, Microarray, *Hordeum vulgare* L, Integrated molecular mechanism

## Abstract

**Background:**

Cadmium (Cd) is a severe detrimental environmental pollutant. To adapt to Cd-induced deleterious effects, plants have evolved sophisticated defence mechanisms. In this study, a genome-wide transcriptome analysis was performed to identify the mechanisms of Cd tolerance using two barley genotypes with distinct Cd tolerance.

**Results:**

Microarray expression profiling revealed that 91 genes were up-regulated by Cd in Cd-tolerant genotype Weisuobuzhi and simultaneously down-regulated or non-changed in Cd-sensitive Dong17, and 692 genes showed no change in Weisuobuzhi but down-regulated in Dong17. Novel genes that may play significant roles in Cd tolerance were mainly *via* generating protectants such as catalase against reactive oxygen species, Cd compartmentalization (e.g. phytochelatin-synthase and vacuolar ATPase), and defence response and DNA replication (e.g. chitinase and histones). Other 156 up-regulated genes in both genotypes also included those encoding proteins related to stress and defence responses, and metabolism-related genes involved in detoxification pathways. Meanwhile, biochemical and physiological analysis of enzyme (ATPase and chitinase), phytohormone (ethylene), ion distribution and transport (Cd, Na^+^, K^+^, Ca^2+^, ABC transporter) demonstrated that significantly larger Cd-induced increases of those components in Weisuobuzhi than those in Dong17. In addition, Cd-induced DNA damage was more pronounced in Dong17 than that in Weisuobuzhi.

**Conclusions:**

Our findings suggest that combining microarray, physiological and biochemical analysis has provided valuable insights towards a novel integrated molecular mechanism of Cd tolerance in barley. The higher expression genes in Cd tolerant genotype could be used for transgenic overexpression in sensitive genotypes of barley or other cereal crops for elevating tolerance to Cd stress.

**Electronic supplementary material:**

The online version of this article (doi:10.1186/1471-2164-15-611) contains supplementary material, which is available to authorized users.

## Background

Cadmium (Cd) in soil represents a direct contact risk to both humans and ecological recipients due to its high toxicity and ready uptake by plants [1, 2]. At present, Cd has become one of the most harmful and widespread pollutants in agricultural soils. This situation has resulted primarily from industrial emissions, application of Cd containing phosphate fertilisers and municipal waste disposal [1, 3]. Once released into the soils, moderate Cd pollution could result in considerable Cd accumulation in edible parts of crops. Such levels of Cd not only affect the quality and yield of crops, but pose a great threat to human health [2]. Accordingly, these developments raise serious concerns for both the environment and human health. Therefore, there is an urgent need to elucidate the mechanisms of Cd tolerance in plants and to develop crop varieties with high Cd tolerance and yield.

Cd affects many important physiological processes and inhibits plant growth and development [4, 5]. To minimise the detrimental effects of Cd toxicity, plants have evolved a range of detoxification mechanisms, including Cd exclusion, chelation and compartmentalisation in vacuoles [6]. For example, phytochelatins (PCs) and Cd have been found to form PC-Cd complexes in cytosol, which are subsequently transported into vacuoles. Thereby, it can protect plants from the deleterious effects of Cd [7]. The elevated expression of *heavy metal transporting ATPases4* (*HMA4*) P_1B_-type ATPase furnishes an efficient mechanism for increasing Cd tolerance in plants under Cd toxicity *via* the maintenance of low cellular Cd in the cytoplasm [8]. However, regulatory mechanisms in Cd-tolerance, which is still a focal point in plant research, is a complex process that contains many genes regulated by a variety of physiological pathways [2].

Identification of stress-induced genes and proteins are fundamental in understanding the molecular mechanisms of stress responses and in developing transgenic plants with enhanced tolerance [9]. Studies have been conducted to identify plant defences against Cd toxicity and investigate Cd-specific genes. For example, the up-regulation of well-known Cd-detoxifying proteins, such as phytochelatin synthase (PCS), antioxidative enzymes and glutathione S-transferases (GST), were observed in the response of plants to Cd stress using proteomic and metabolomic approaches [10]. Uraguchi and Fujiwara [2] summarised several Cd transport-related genes, such as *OsLCT1*, *OsHMA*, *OsNramp1* and *OsIRT1*, involved in Cd transport and tolerance in rice. Despite the identification of those genes, the underlying knowledge of molecular mechanisms for plant Cd tolerance is still fragmental.

Knowledge about networks of global gene expression is imperative for further understanding the molecular mechanisms in plant Cd tolerance. Microarray analysis is a powerful technique for analysing the profiles of gene expression related to abiotic stress in plants [11, 12]. Concerning Cd toxicity and detoxification in plants, the genome-wide transcriptome profiling has been explored mostly in herbaceous plants such as *Arabidopsis thaliana*, *Arabidopsis halleri*, and *Thlaspi caerulescen* [13–16]. However, the regulatory system for genes conferring Cd tolerance in many cereal crops is largely unknown and remains an essential issue to be addressed.

Barley, the fourth most important cereal in the world, is as an ideal model for genetic and physiological study [17]. Our previous studies have demonstrated distinctive genotypic difference between Cd-tolerant (Weisuobuzhi) and Cd-sensitive (Dong17) genotypes in response to Cd stress including plant growth, photosynthesis, antioxidant enzyme activities [1, 18, 19]. We, therefore, hypothesised that there were large differences between the two genotypes in their genome-wide response to Cd stress. Candidate genes associated with Cd tolerance in the tolerant genotype were identified using the Affymertix barley GeneChip whole genome arrays, and the expression and enzyme activity of many key genes were further validated by quantitative real-time PCR (qRT-PCR) and physiological and biochemical analysis. We also proposed an integrated schematic diagram of the mechanisms involved in Cd tolerance and adaptation. These results are very important to provide novel clues for understanding the mechanisms in Cd tolerance of barley and open prospective for excavating novel genes and for the genetic improvement of plant tolerance to Cd stress.

## Methods

### Plant material, growth conditions and Cd treatments

A greenhouse hydroponic experiment was carried out at Huajiachi Campus of Zhejiang University, Hangzhou, China. Two barley genotypes, Cd-tolerant Weisuobuzhi and Cd-sensitive Dong17 [1], were used throughout the experiments.

Barley seeds were surface sterilised by soaking in 2% H_2_O_2_ for 30 min and fully rinsed with deionised water. After soaking in deionised water at room temperature for 4 h, seeds were germinated in sterilised moist sand in an incubator at 22 ± 1°C. Ten-day-old healthy and uniform plants were selected and transplanted to 5-L containers filled with 4.5 L basal nutrient solution. The composition of nutrient solution was described by Chen et al. [18, 19]. A week after transplanting, Cd (as CdCl_2_) was added to the corresponding containers to form 4 treatments: basal nutrient solution (control, without Cd) and 5, 50 and 500 μM Cd. The experiment was laid in a split-plot design with Cd-treatment as main-plot with six replicates. The nutrient solution was continuously aerated with pumps and renewed every 5 d.

Plant samples were harvested 15 d after Cd treatment, and root tips were used for fluorescence imaging of Cd. The second fully expanded leaves of control and 5 μM Cd treatment were collected for microarray, physiological and biochemical analysis.

### Fluorescence imaging

Fresh root tips were immersed in 20 mM disodium ethylenediamine tetra-acetic acid (Na_2_-EDTA) for 15 min and then gently rinsed for three times with deionised water. The specimen sections were then immediately immersed in the Cd probe Leadmium™ Green AM solution (Molecular Probes, Life Technologies, California, USA) for 45 min in the dark and then washed three times (5 min each time) with deionised water. A stock solution of Leadmium™ Green AM was made by adding 50 μL of dimethyl sulfoxide (DMSO) to one vial of the dye. This stock solution was then diluted 1:10 with 0.85% NaCl. Roots were immersed in this solution for 90 min in the dark. The sections were examined with a laser confocal scanning microscope (Leica TCS SP5; Berlin, Germany) with excitation and emission wavelengths at 488 and 515 nm, respectively. The fluorescence density of Cd was calculated by selecting the root sections and measuring the total Integrated Density using “Analyse and Measure” function of the Image J software (NIH, Bethesda, MD, USA).

### RNA isolation, probe preparation and microarray hybridisation

Total RNA was initially extracted from leaf tissue of Weisuobuzhi and Dong17 under control and 5 μM Cd treatment using Trizol reagent protocol (P/N 15596–018, Life Technologies, Carlsbad, CA, USA). RNA was purified on RNeasy spin columns (RNeasy MinElute Cleanup Kit, Qiagen, Dusseldorf, Germany) and with on-column DNase1 treatment. The eluted total RNAs were quantified on an Agilent 2100 bioanalyser (Palo Alto, CA, USA) and adjusted to a final concentration of 1 μg μL^-1^. Sample processing, cDNA synthesis, biotin-labelled cRNA synthesis, hybridisation, washing, staining and scanning of Affymetrix Barley 1 GeneChip were performed following the standard protocol (Affymetrix Inc., Santa Clara, CA, USA). The Barley 1.0 Affymetrix microarray GeneChip array consists of 22,795 probe sets designed from an exemplar set of barley sequences derived from 350,000 high-quality expressed sequence tag (EST) contigs. Total RNA was used to prepare double strand cDNA. Labelled cRNA preparation and hybridisation on GeneChip and scanning was done following the standard Affymetrix procedures (http://media.affymetrix.com/support/downloads/manuals/3_ivt_express_kit_manual.pdf). Three replications of each sample were conducted to test the quality and reproducibility of the chip hybridisation. Each treatment had three replications.

### Microarray data analysis

Data analysis was conducted using Refiner and Analyst, two analytical tools in Affymetrix GeneChip Operating Software Version 1.4. The Refiner tool condenses and normalises the raw signal. The Analyst tool provides statistical analysis and data visualisation capability. Detection, signal condensation and normalisation were conducted using Robust Multichip Analysis (RMA) of the Refiner tool [20]. Further analysis of transcript abundance was conducted using the Analyst tool. The correlation of expression signals between replications for each genotype was 0–0.99 across all probe sets tested on the GeneChip. Each Barley1.0 GeneChip probe set was first tested for barley transcript specificity on the basis of quantitative and qualitative transcript abundance differences between barley genotype Betzes and wheat cultivar Chinese Spring (CS).

To detect barley transcripts in the two lines (Weisuobuzhi and Dong17), differences in the abundance of barley transcript signals in the two lines were tested against the background signal in CS with a *t*-test (*P*-value < 0.001). Only those showing significantly higher transcript levels both in Betzes and in the two lines compared to those in CS were subjected to qualitative analysis to detect barley-specific transcripts in the two lines (presence/absence test, *P* < 0.001 using MAS 5.0). All data from the Affymetrix scanner have been deposited at BarleyBase (http://www.barleybase.org) in the form of DAT, CEL, EXP, and CHP files. We considered a change of at least two folds as an indication of a significant change in gene expression for up- and down-regulation. To perform hierarchical clustering, the differentially regulated genes were clustered according to similarities in expression profiles. The following nine groups were used: Weisuobuzhi (W) up-regulated and Dong17 (D) down-regulated (Group I) or no change (Group II), W no change and D down-regulated (Group III), both up-regulated (Group IV), W down-regulated and D up-regulated (Group V), both down-regulated (Group VI), W down-regulated and D no change (Group VII), W no change and D up-regulated (Group VIII), and both no change (Group IX).

### qRT-PCR

The hydroponic experiment was carried once again using Weisuobuzhi and Dong17 under control and 5 μM Cd treatment with four replicates. Total RNA was isolated from leaves after 15 days of Cd treatment using the TRIzol reagent (Invitrogen, Karlsruhe, Germany). Residual DNA was removed using purifying columns. One microgram of each RNA sample was subsequently employed for cDNA synthesis with 0.5 μg of oligo (dT) 12–18 and 200 units of Superscript II (Invitrogen, Karlsruhe, Germany). cDNA samples used for GeneChip analysis and this experiment were assayed by quantitative real time PCR (qRT-PCR) in an iCycler iQ™ Real-time PCR Detection System (Bio-Rad, Hercules, CA, USA) using the SYBR Green PCR Master Mix (Applied Biosystems, Life Technologies, CA, USA). The PCR conditions consisted of denaturation at 95°C for 3 min, followed by 40 cycles of denaturation at 94°C for 1 min, annealing at 58°C for 30 s and extension at 72°C for 30 s, and continued extension at 72°C for 5 min. The barley *ACTIN* (accession no. AY145451) was used as a reference gene, which were selected from a number of candidates by Chen et al. [18, 19]. The primers are listed in Additional file 1: Table S1. Two independent experiments and six biological replications in total were conducted.

### Enzyme activity measurements

For the determination of GST (EC 2.5.1.18) and ATPase (EC 3.6.1.3) activities, plant tissue was homogenised in 8 ml 50 mM sodium phosphate buffer (PBS, pH 7.8,) using a pre-chilled mortar and pestle, then centrifuged at 10000 × g for 20 min at 4°C. The supernatant was used for enzyme assay. GST and ATPase activities were determined with an enzyme assay kit according to the manufacturers’ protocol (Jiancheng Bio Co., Nanjing, China). Chitinase (EC 3.2.1.14) were extracted and determined with an ELISA kit according to the manufacturers’ protocol (Becton, Dickinson and Company, FranklinLakes, NJ, USA).

### Determination of DNA damage

DNA damage was assayed according to Menke et al. [21] with minor modification. The experiment was performed in a darkroom with dim red light. About 150 mg leaf samples were sliced in 1 mL PBS buffer (160 mM NaCl, 8 mM Na_2_HPO_4_, 4 mM NaH_2_PO_4_, pH 7) containing 50 mM EDTA on ice with a fresh razor blade. The 30 μL suspension was then taken on regularly used microscopic slides (pre-coated with 1% of agarose in double distilled H_2_O and dried over night at room temperature) followed by addition of 30 μL 1% agarose solution (42°C). DNA damage was analysed by the comet assay according to the alkali-alkali (A/A) method as described by Menke et al. [21]. For comet assay, unwinding was done in high alkali for 5 min, and then electrophoresis for 10 min with 21 V, 300 mA in a chamber cooled on ice, followed by a short neutralisation of 3 min in 100 mM Tris–HCl (pH 7.5). To remove the starch grains, the slides were kept for 10 min in 1% Triton prior to dehydration in 70% (2 min) and 96% (5 min) ethanol and air-drying. The gels were then stained with 15 μL ethidium bromide (5 μg ml^-1^) and immediately used for evaluation. Images were taken using a fluorescence microscope (BX50WI, Olympus, Japan) equipped with a digital CCD camera (Olympus, Japan).

### Gas chromatography

Ethylene production in the leaves of barley seedling was measured as described by Chen et al. [22] with minor modification. In brief, leaf tissues (0.5 g fresh weight) were collected, immediately weighed, and then placed in sealed glass vials containing water-saturated filter paper. Samples were incubated for 1 h under illumination. One millilitre of gas was collected using a gas-tight syringe and injected into a gas chromatography (Focus GC, Thermo Fisher Scientific, MA, USA) equipped with a capillary column (CP-carboPLOT P7, Varian, CA, USA) and flame-ionisation detector for ethylene determination. Ethylene production was calculated on the basis of known ethylene standards and leaf fresh weight.

### Leaf element analysis

Leaf samples were dried at 80°C, digested with HNO_3_/HClO_4_ (4:1, v/v) for 3 h, and then diluted to 25 ml by adding de-ionized water. Calcium (Ca^2+^), sodium (Na^+^), and potassium (K^+^) concentrations were determined using an Inductively Coupled Plasma Optical Emission Spectrometer (ICP-OES, Optima 8000DV, PerkinElmer, USA).

### Statistical analysis

All data are the averages of three to six biological replicates in each experiment. Statistical analyses were performed with Data Processing System (DPS) statistical software. An ANOVA followed by a Duncan’s multiple range test (DMRT) were used to evaluate treatment effects at significance level of *P* < 0.05.

## Results

### Cd is highly accumulated in root tips of a Cd-tolerant genotype

**Figure 1:**
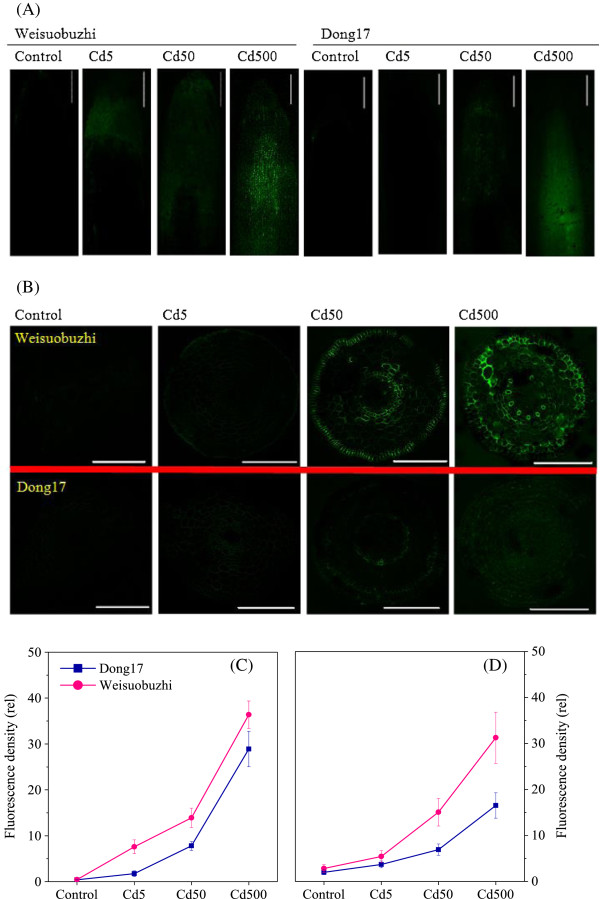
**Cellular localisation of Cd** ^**2+**^ **in barley roots.** Barley roots were exposed to 0, 5, 50 and 500 μM Cd for 15 days before staining with Leadamium™ Green AM. Representative micrographs show the binding of Cd^2+^ to Leadamium™ Green AM dye at longitudinal **(A)** and cross **(B)** section of root tips of barley genotypes Weisuobuzhi and Dong17. Scale bars =250 μm. Line graphs show relative Cd^2+^ fluorescence density from longitudinal **(C)** and cross **(D)** section of root tips. Data are means ± SD (n = 3).

Under Cd stress, the majority of Cd accumulated in root cell wall. In root tips of Weisuobuzhi treated with 5 μM Cd, preferential localisation of Cd was in root apex, and this effect was more pronounced with increasing Cd levels (Figure 1A and 1C). However, there were no noticeable and only very low levels of green fluorescence in the root tips of Dong17 under 5 and 50 μM Cd, respectively (Figure 1A and 1C). Higher fluorescence intensity was observed in 500 μM Cd treatment in Dong17, which was similar to that of Weisuobuzhi. The cross section fluorescence images revealed that after Cd stress, most Cd accumulated in the inner epidermis and endodermis, with only a small amount of Cd in the cortex. In response to 500 μM Cd, an intense green fluorescence was observed in the epidermal, cortical and stele cells of Weisuobuzhi but not in Dong17 (Figure 1B and 1D).

### Cd toxicity induces large scale changes in gene expression

The distinct cellular Cd accumulation pattern (Figure 1) and tolerance [18, 19] of the two genotypes has led us to explore the expression pattern of their Cd-induced genes. The microarray data showed that, compared with control, the gene expression profiles of the two genotypes changed significantly after exposing to 5 μM Cd for 15 d. Based on at least ±2.0-fold changes (*P* < 0.05), a total of 1,750 genes were differentially expressed between Cd-stressed and control plants. Of these genes, only 247 and 103 genes were up- and down-regulated in Weisuobuzhi, while there were 3.6- (898) and 6.9-fold (710) more genes up-regulated and down-regulated in Dong17, respectively (Additional file 2: Figure S1 and Additional file 3: Table S2).

**Figure 2:**
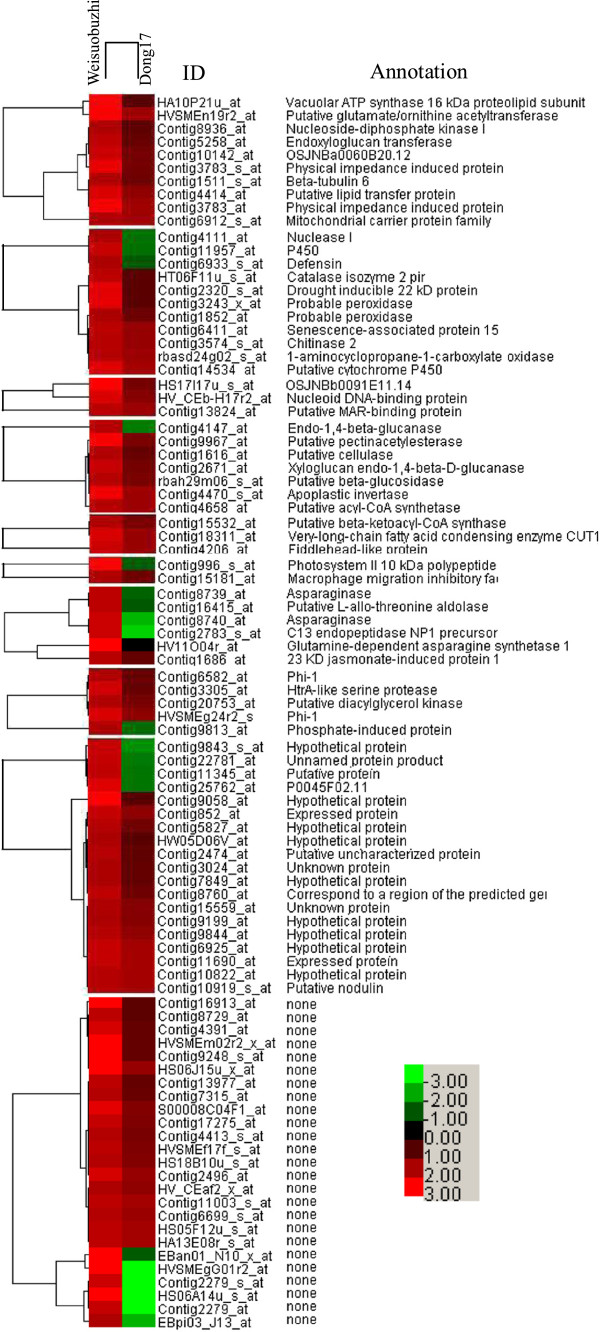
**Cd-induced differential gene expression in leaves of two barley genotypes.** Heat map visualises the expression of genes up-regulated in Weisuobuzhi and down-regulated and not changed in Dong17 (Cd *vs* control) after Cd exposure for 15 d. The contig IDs and annotations are listed on the right. Red, green and black indicate genes that increased, decreased and showed equal levels of expression, respectively, as compared to the control. The contig ID and annotation of each gene are listed on the right of the figure. The identity and accession numbers of genes are listed in Additional file 4: Table S3 and Additional file 5: Table S4.

Further comparing the transcriptome responses to Cd stress between the two genotypes, these 1,750 differentially regulated genes were classified into eight groups (Additional file 3: Table S2). The genes in Group I and Group II were highly induced by Cd only in Weisuobuzhi (Figure 2, Additional file 4: Table S3 and Additional file 5: Table S4). Genes in Group III showed no change in Weisuobuzhi and were down-regulated in Dong17 (Additional file 6: Table S5). Group IV represented 156 genes that showed up-regulated expression patterns in both genotypes (Additional file 7: Table S6). The genes in Groups V, VI and VII were down-regulated in Weisuobuzhi (Additional file 8: Table S7, Additional file 9: Table S8 and Additional file 10: Table S9). The genes in Group VIII were un-regulated in Weisuobuzhi but up-regulated in Dong17 (Additional file 11: Table S10).

**Figure 3:**
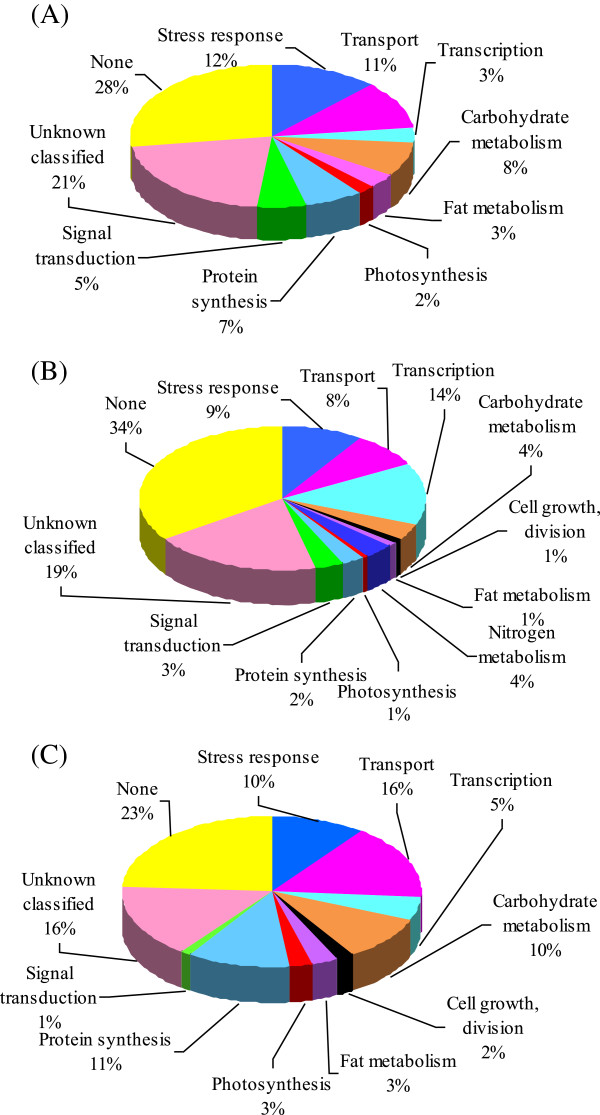
**Functional categorisation and differential expression of Cd stress-regulated genes in barley leaves.** Functional categorisation was performed according to the agriGO methods. Pie charts show the distribution of different functional genes after exposing the plants to 5 μM Cd for 15 days. **(A)** up-regulated in Weisuobuzhi and down-regulated and not changed in Dong17 (Groups I and II); **(B)** not changed in Weisuobuzhi and up-regulated in Dong17 (Group III); **(C)** up-regulated in both genotypes (Group IV).

Here, we focused on the genes in Groups I, II and III, which are more likely to play crucial roles for Cd tolerance in barley. Along with these differentially regulated genes related to stress and defence responses, there were also functional genes encoding a number of carbohydrate metabolism related proteins and several transcription factors, along with a diverse set of enzymes and transporters (Figure 3; Additional file 12: Figure S2, Additional file 3: Table S2, Additional file 4: Table S3, Additional file 5: Table S4, Additional file 6: Table S5, Additional file 7: Table S6, Additional file 8: Table S7, Additional file 9: Table S8, Additional file 10: Table S9 and Additional file 11: Table S10). Therefore, functional categorisation of these genes may provide clues to the understanding of physiological and molecular mechanisms involved in Cd stress response. The data pertinent to Cd tolerance were presented in the following sections (Additional file 4: Table S3, Additional file 5: Table S4 and Additional file 6: Table S5).

### Key genes for Cd tolerance are highly expressed only in the Cd-tolerant genotype

These genes were up-regulated in Cd-tolerant genotype Weisuobuzhi (W-*up*) and down- (D-*down*) or un-regulated (D-*none*) in Cd sensitive genotype Dong17 (Additional file 4: Table S3 to Additional file 5: Table S4, Figure 3A). Group I included 7 genes encoding key enzymes for nitrogen catabolism such as asparaginase and C13 endopeptidase NP1 precursor. The 84 genes in Group II were up-regulated after Cd treatment in Weisuobuzhi but showed no change in Dong17 (Additional file 5: Table S4). Of these, there were 6 signal transduction related genes (e.g. the gene encoding 23 KD jasmonate-induced protein 1), 11 stress and defence response related genes (such as the genes encoding catalase isozyme 2, peroxidase, and chitinase 2), 10 carbohydrate and fat metabolism related genes (e.g. the gene encoding acetyl-CoA synthetase). Additionally, the expression of 10 transporter genes (e.g. the genes encoding vacuolar ATP synthase, 16 kDa proteolipid subunit and putative lipid transfer protein) under Cd stress was up-regulated in Weisuobuzhi but showed no change in Dong17.

### Key genes for Cd-tolerance are down-regulated only in the Cd-sensitive genotype

The majority of the 692 genes in Group III were responsible for stress and defence response, transport, transcription and signal transduction (Additional file 6: Table S5, Figure 3B). For instance, the salicylic acid (SA), jasmonate (JA), ethylene (ET) and Ca^2+^ induced genes were all significantly down-regulated in Cd-sensitive genotype Dong17, but were not affected in Weisuobuzhi after 15 d of Cd exposure, with the exception of a 23 kDa JA-induced protein that was up-regulated in Weisuobuzhi (Additional file 5: Table S4 and Additional file 6: Table S5). There were 2 isoforms of GST showing differential expression in both genotypes (Additional file 6: Table S5). Genes encoding nitrate (NRaT) and nitrite (NRiT) transporters, as well as a gene encoding dehydrins (late embryogenesis abundant, LEA) were down-regulated in Dong17 but remained unchanged in Weisuobuzhi. Heat shock proteins (HSPs) were highly down-regulated under Cd stress in Dong17.

### qRT-PCR confirms expression pattern of the microarray

**Figure 4:**
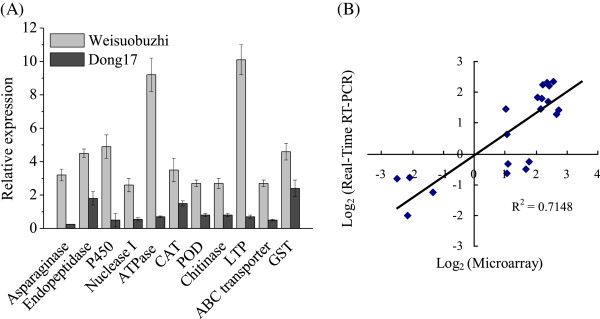
**Quantitative RT-PCR validations of microarray data of Cd-responsive key genes in barley leaves. (A)** The values represents the gene expression in 5 μM Cd over those in the control in Weisuobuzhi (grey bars) and Dong17 (black bars) (n = 6 biological replicates). *ACTIN* was used as the reference gene. **(B)** Data plotted are the mean ratio of gene expression in Cd treatment (5 μM Cd for 15 d) over those in the control in the two genotypes on a log2 scale (r^2^ = 0.7148; *P* < 0.001). CAT, catalase isozyme; POD, peroxidase; P450, cytochrome P450; LTP, lipid transfer protein and GST, glutathione transferase.

The expression data obtained from microarray analysis was further confirmed using qRT-PCR. The expression profile of eleven differentially expressed genes that were related to Cd tolerance in the two genotypes is shown in Figure 4. The expression patterns were similar, although the qRT-PCR-based relative expression of those genes did not exactly match the fold changes found in the microarray analysis.

### Biochemical and physiological validation of the roles of key genes in Cd tolerance

**Figure 5:**
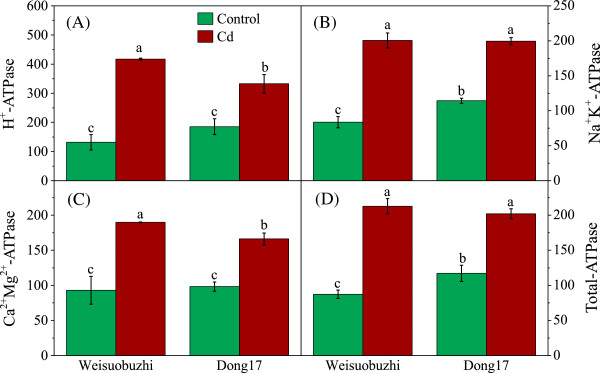
**Comparison of ATPase activity in leaves of two barley genotypes.** H^+^-ATPase **(A)**, Na^+^K^+^-ATPase **(B)**, Ca^2+^Mg^2+^-ATPase **(C)**, and total ATPase **(D)** of Weisuobuzhi and Dong17 were measured in the control and after 15 d of 5 μM Cd treatment. Data are means ± SD (n = 4). The value of total-ATPase activity is not the sum of all ATPase activities due to different methods used in the assay. Different lowercase letters in each graph indicate significant differences at *P* < 0.05.

**Figure 6:**
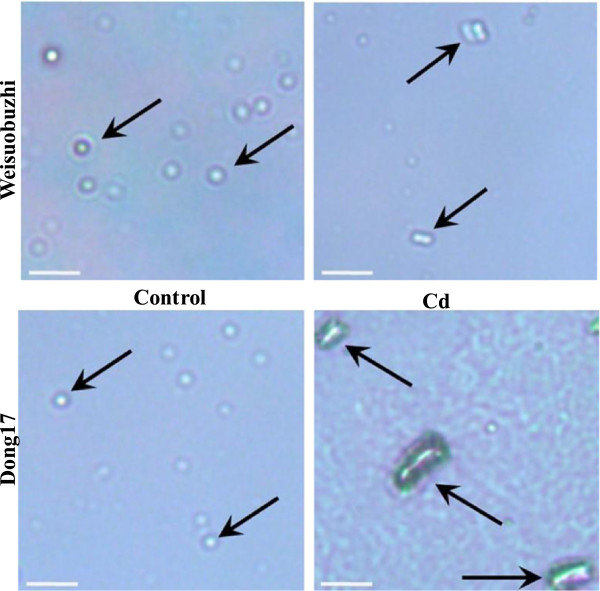
**Visualisation of DNA damage in leaves of two barley genotypes.** Representative images are shown for samples in the control and after 15 d of 5 μM Cd treatment (n = 3). Scale bars = 50 μm.

To test whether the higher levels of transcripts in Weisuobuzhi are linked to Cd tolerance in barley, we conducted a series of assays on enzyme activity (Figure 5, Table 1), DNA damage (Figure 6), ethylene emission (Table 1) and essential nutrients (Table 2). Overall, the microarray and qRT-PCR data were in good agreement with these biochemical and physiological results.

**Table 1:** **Effects of 15 d of Cd treatment on ethylene emission and the activities of glutathione S-transferases (GST) and chitinase in leaves of two barley genotypes**

Treatment	Ethylene emission (nL g ^-1^ FW h ^-1^)	GST (unit g ^-1^ FW)	Chitinase (unit g ^-1^ FW)
***Weisuobuzhi***			
Control	0.042c	95.2b	172.1c
5 μM Cd	1.136a	200.1a	218.1a
	(+35.6%)*	(+4.2%)	(+16.1)
***Dong17***			
Control	0.019c	210.8a	162.3c
5 μM Cd	0.838b	192.0a	187.8b

FW, fresh weight; Different lowercase letters indicate significant difference at *P* < 0.05 level (n = 4). *, relative increase (+) percentage calculated by 100 *(Weisuobuzhi-Dong17)/Dong17 under Cd stress.

**Table 2:** **Effects of 15 d of Cd treatment on Ca** ^**2+**^ **, Na** ^**+**^ **, and K** ^**+**^ **content in leaves of two barley genotypes**

Treatment	Ca ^2+^ (g kg ^-1^ DW)	Na ^+^ (g kg ^-1^ DW)	K ^+^ (g kg ^-1^ DW)	Na ^+^/K ^+^ (%)
***Weisuobuzhi***				
Control	6.30a	1.76b	89.4ab	1.97b
5 μM Cd	5.55b	1.23d	86.8b	1.42c
	(+18.3%)*	(-18.0%)	(+7.2%)	(-23.6%)
***Dong17***				
Control	6.16ab	2.08a	91.0a	2.29a
5 μM Cd	4.69c	1.50c	81.0c	1.86b

DW, dry weight; Different lowercase letters in each column indicate significant difference at *P* < 0.05 level (n = 4). *, relative increase (+)/reduction (-) percentage calculated by 100 *(Weisuobuzhi-Dong17)/Dong17 under Cd stress.

In comparison to the control, Cd stress resulted in significant increases in the activity of H^+^-, Na^+^K^+^-, Ca^2+^Mg^2+^- and total-ATPase of 217%, 140%, 104%, and 143% in Weisuobuzhi, but those numbers were only 80%, 74%, 69% and 72% for Dong17, respectively (Figure 5). Moreover, Cd stress caused a 110% increase in GST activity in Weisuobuzhi but no statistically significant change was observed in Dong17 (Table 1). The significant increase of chitinase activity under Cd stress was 1.7-fold higher in Weisuobuzhi than that in Dong17 (Table 1).

DNA appeared as densely condensed structured resembling a bead and no DNA damage was observed in both genotypes in the control (Figure 6). However, Cd stress induced a marked increase in DNA damage in the Cd-sensitive genotype Dong17. Moreover, Cd stress led to a significant increase in ethylene emission in both genotypes. However, the rate of ethylene emission was significant higher in Weisuobuzhi than that in Dong17 (Table 1).

Cd treatment resulted in a significant decrease of leaf Ca^2+^ concentration, which was 2.0-fold higher for Dong17 in contrast to Weisuobuzhi. Cd stress caused an 11.0% decrease of K^+^ content in Dong17, but no significant difference was observed in Weisuobuzhi. Surprisingly, Na^+^ content was also markedly reduced under Cd stress in both genotypes (Table 2).

## Discussion

### Comparative transcriptome analysis reveals key genes associated with Cd tolerance

**Figure 7:**
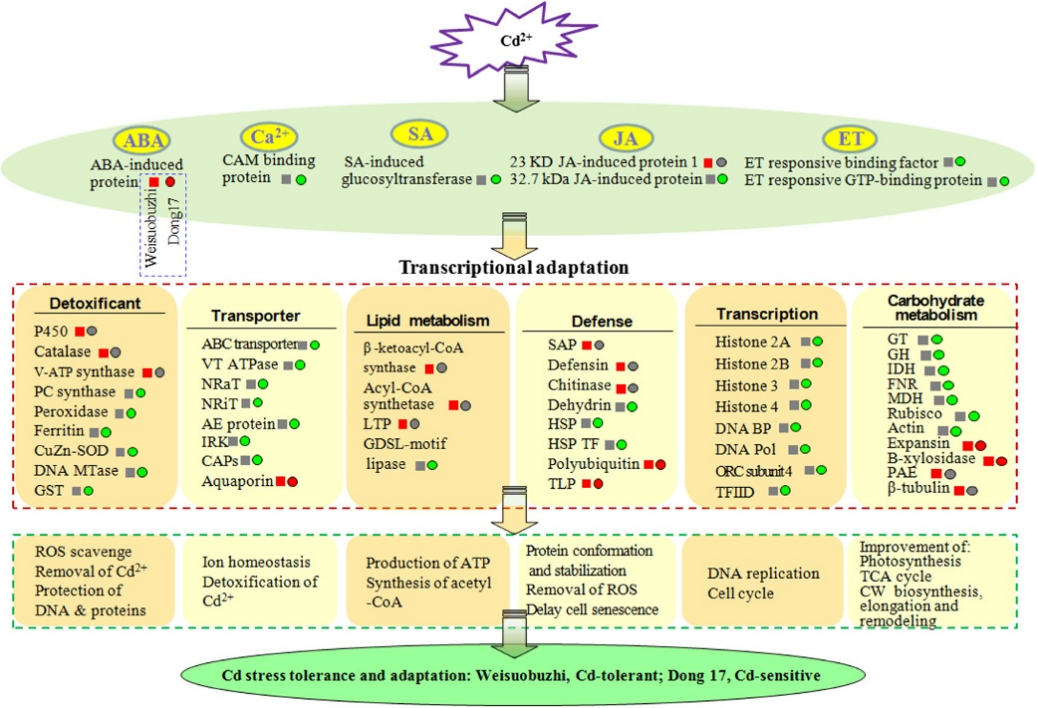
**Integrated schematic diagram of the mechanisms involved in Cd tolerance in barley.** Genes labelled with red, grey and green squares (Weisuobuzhi) circles (Dong17) are up-regulated, not changed and down-regulated by 5 μM Cd treatment, respectively. ABA, Abscisic acid; AE protein, Anion exchange protein; CAPs, Clathrin-associated protein; CAM, calmodulin; CAT; Catalase isozyme 2; CW, Cell wall; DNA BP, DNA binding protein; DNA MTase, DNA methyltransferase; DNA Pol, DNA polymerase; ET, Ethylene; FNR, Ferredoxin NADP(H) oxidoreductases; GH, Glycosyl hydrolase; GST, Glutathione transferase; GT, Glycosyl transferase; HSP, Heat shock protein; HSP TF, Heat shock transcription factor; IDH, Isocitrate dehydrogenase; IRK, Inwardly rectifying potassium channel; JA, jasmonate; LTP, Lipid transfer protein; MDH, Malate-dehydrogenase; NRaT, Nitrate transporter; NRiT, Nitrite transport protein; ORC subunit 4, Origin recognition complex subunit 4; PAE, Pectinacetylesterase; PC, phytochelatin; SA, Salicylate; SAP, Senescence-associated protein; TFIID, Transcription initiation Factor TFIID; TLP, Thaumatin-like proteins; V-ATP synthase, Vacuolar ATP synthase; VT ATPase, Vesicle transfer ATPase.

In barley, no molecular evaluation of Cd tolerance mechanisms has been fully explored so far. This study used large-scale transcript profiling to examine cellular processes affected by Cd stress in leaves of Cd-tolerant Weisuobuzhi and Cd-sensitive Dong17. A number of key genes have been shown to be induced or repressed differently in the two contrasting barley genotypes under Cd stress. Based on these identified Cd-responsive genes, we propose an integrated schematic diagram of the mechanisms involved in Cd tolerance and adaptation (Figure 7) and a specific model for compartmentalization (Additional file 13: Figure S3), which may provide novel clues towards the characterisation of molecular mechanisms underlying Cd tolerance in barley.

### Detoxification genes are largely responsible for Cd tolerance in barley

One aspect of Cd tolerance and adaptation is the ability to scavenge Cd-induced reactive oxygen species (ROS) in order to protect membranes and other macromolecules [3]. Antioxidant enzymes, such as catalase (CAT) and CuZn-superoxide dismutase (SOD), play key roles in scavenging ROS under heavy metal stress [23]. GST includes a family of potent detoxification enzymes, and is thought to contribute to the primary cellular defence against oxidative stress [24]. In this study, the expression levels of the genes encoding CAT2 and CuZn-SOD confirmed the higher enzyme activity in Weisuobuzhi in our previous publication [19]. Also, higher transcripts of GST found in both microarray and qRT-PCR were matched by a significantly elevated GST activity in Weisuobuzhi as compared to a small decrease in Dong17 (Table 1; Figure 4; Additional file 5: Table S4 and Additional file 6: Table S5). As a result, Dong17 accumulated more O_2_^**.**^, H_2_O_2_ and MDA than Weisuobuzhi [19].

Membrane bound enzyme ATPases provide energy and an H^+^ gradient for the co-transport of copper, cobalt, lead, and Cd with protons for the detoxification of these metal ions in plants [25, 26]. Cation antiporter activity driven by vacuolar ATPase-dependent proton motive force contributes significantly to the detoxification of Cd *via* vacuolar compartmentalisation in plants [27]. In this study, transcripts of V-ATPase were significantly elevated in Weisuobuzhi but showed no change in Dong17 (Additional file 5: Table S4). In addition, we validated the microarray data using biochemical analysis with significantly higher Cd-induced H^+^-, and Ca^2+^Mg^2+^-ATPase and much larger Cd-induced increase in Na^+^K^+^- and total-ATPase activities in Weisuobuzhi (Figure 5).

DNA methylation is an important modification of DNA that plays a key role in gene regulation, DNA replication and repair, and chromatin determination [28]. In the present study, Cd significantly inhibited DNA methyltransferase in Dong17 but showed no change in Weisuobuzhi, which represented a 2.6-fold higher relative expression of this gene in Weisuobuzhi (Additional file 6: Table S5). In addition, Cd stress induced a marked increase in DNA damage to the Cd-sensitive genotype Dong17 (Figure 6). Phytochelatins are widely accepted as a major agent of plant detoxification and tolerance to Cd stress [7] and a lack of PC synthase activity resulted in an increased sensitivity to Cd [29]. Our previous research has shown that genotypic differences in Cd tolerance were positively linked to the elevation of PCs in rice [30]. In this study, transcripts of PC synthase were significantly inhibited in Dong17, while no change was found in Weisuobuzhi (Additional file 6: Table S5). In summary, the results demonstrated that Cd-tolerant Weisuobuzhi is more capable to scavenge Cd-induced ROS by increasing the activity of antioxidant enzymes, to maintain DNA structural stability and protect the normal DNA methylation, and to sequester more Cd^2+^ to vacuole to reduce Cd toxicity.

### Membrane transport genes modulate ion homeostasis for Cd tolerance

Cd affects the distribution of nitrogen, but nitrogen could be recycled and be translocated as a Cd protection and storage strategy [31]. Li et al. [32] reported that nitrate transporter NRT1.8-regulated nitrate distribution plays an important role in Cd tolerance. In addition, inwardly rectifying K^+^ channels (IRK) contributes to cellular K^+^ homeostasis in higher plants [33]. In the present study, the genes encoding transporters for nitrogen (NRaT and NRiT) and potassium (IRK) were significantly decreased in Dong17 but remained unchanged in Weisuobuzhi (Additional file 6: Table S5). Cd stress also induced a significant decrease of K^+^ content in Dong17 but no change in Weisuobuzhi (Table 2), indicating a role for IRK in the reduced K^+^ uptake in Dong17. Moreover, ABC transporters are involved in the homeostasis of organic anions, heavy metals, xenobiotics and lipids [34], including vacuolar compartmentalisation of Cd [35]. The ABC transporters of *Arabidopsis*, AtMRP3 and AtATM3, have been shown to confer Cd resistance [35, 36]. AtPDR8 is a Cd extrusion pump conferring Cd and Pb resistance [34]. However, the genes encoding ABC transporters, in this study, were significantly inhibited in Dong17 while not affected in Weisuobuzhi (Additional file 6: Table S5). Therefore, a higher Cd accumulation in Weisuobuzhi (Figure 1) did not affect its overall Cd tolerance, but rather supported the hypothesis that more Cd is transported into the vacuoles of Weisuobuzhi, alleviating Cd toxicity to the cytoplasm.

As a result, these data enable us to generate a specific model on key components of vacuolar compartmentalisation for Cd tolerance (Additional file 13: Figure S3). Interestingly, ten key genes found to be down-regulated in the Cd-sensitive Dong17, were unchanged in Weisuobuzhi. All these highlighted that the high Cd accumulation in Weisuobuzhi (Figure 1; Chen et al. [1]) is linked with expression patterns of those specific genes involved in vacuolar compartmentalization, which is one of the most crucial strategies for Cd tolerance in plants.

### Hormonal and Ca^2+^ signal transduction related genes are important for Cd tolerance

Phytohormones such as SA, JA and ET play fundamental roles *via* signalling crosstalk in abiotic stresses in plants [37]. For instance, Cd stress triggered an accumulation of ET in bean [38]. It has also been suggested that ET signalling pathways affect the early phase of Cd stress response, and 1-aminocyclopropane-1-carboxylic acid oxidase (ACO) catalyses the last step of ET biosynthesis in Arabidopsis [14]. Transcripts of ACO was markedly increased in Weisuobuzhi but was no change in Dong17 (Additional file 5: Table S4). ET activates stress-responsive genes in the hormonal signalling cascade against Cd toxicity. However, transcripts of ET-induced proteins were all significantly decreased in Dong17 (Additional file 6: Table S5). Cd-induced ET emission was also lower in Dong17 compared to that in Weisuobuzhi, which was consistent with the transcripts of ACO (Table 1; Additional file 5: Table S4). These results may suggest that Weisuobuzhi is more likely to defend itself against Cd stress through ET signalling. Meanwhile, Ca^2+^-binding protein calmodulin (CAM) transduces second messenger signals into a wide range of cellular responses [39]. Accumulation of Cd^2+^ may compete with cellular Ca^2+^ for CAM binding sites [40]. Under Cd stress, there was significantly less leaf Ca^2+^ in Dong17 than that in Weisuobuzhi (Table 2). Our results showed that Cd induces more signal molecules and activate Cd responsive genes more rapidly in Cd-tolerant Weisuobuzhi than those in Dong17. This difference again contributes to the high Cd-tolerance of Weisuobuzhi.

### Carbohydrate metabolism related genes regulate cell wall structure for Cd tolerance

Cell wall, consisting of cellulose, hemicelluloses and pectin, which contain carboxyl, hydroxyl and aldehyde, can sequester a substantial amount of Cd under Cd stress [5]. For instance, Cosio et al. [41] found Cd binding in leaf cell wall could play a major role in Cd tolerance and hyperaccumulation in *Thlaspi caerulescens* and *Arabidopsis halleri*. Xiong et al. [42] found that 200 μM Cd markedly decreased the pectin and hemicellulose content in rice root cell wall. Glycosyl hydrolases (GH) are also essential for the modification of cell wall polysaccharides [43]. In the present study, GH and GT were found to be down-regulated in Dong17, but showed no change in Weisuobuzhi. Hence, Weisuobuzhi could maintain normal cell wall synthesis, remodelling and modification, and consequently have the capacity to accumulate much higher Cd at the cell wall (Figure 1), therefore reducing Cd toxicity to the cytoplasm.

### Defence and DNA replication related genes are critical for Cd tolerance

Chitinases are components of plant defences against high concentrations of heavy metals such as Cd and As [44]. Chitinase 2 genes showed significant up-regulation under Cd stress in Weisuobuzhi (Additional file 5: Table S4), which was confirmed by the elevated chitinase activity (Table 1). Moreover, Cd affects cell cycle progression, differentiation, DNA replication and repair [45]. Histones such as H2B may be able to repair heavy metal-induced DNA damage in plant cells [46, 47]. In the current study, transcripts of H2A, H2B, H3 and H4 were all significantly down-regulated in Dong17 but were not modified in Weisuobuzhi (Additional file 6: Table S5). Also, genes encoding DNA replication related proteins (e.g. DNA binding protein, polymerase and origin recognition complex subunit 4) were all significantly inhibited in Dong17 under Cd stress, while no change was found in Weisuobuzhi (Additional file 6: Table S5). Considering the significant Cd-induced DNA damage found in Dong 17 (Figure 6), we thus suggest that defence and DNA replication related proteins like Chitinases and histones could be key determinants for Cd tolerance in barley.

### Cd-responsive miRNAs showed a negative link to the expression of key genes in this study

miRNAs are a large family of small non-coding RNAs that negatively regulate mRNA at the post-transcriptional level [48]. Establishing a link between published miRNAs in the literature and our microarray data could provide a better understanding and validation to further investigate the key genes in Cd tolerance in plants. We identified 13 genes, showed significant negative correlation (r^2^ = 0.441; *P* < 0.001) between the microarray data (Additional file 14: Table S11) and different miRNA families [48–51]. The results demonstrated combination of microarray and miRNA analysis could narrow down the number of candidate genes conferring Cd tolerance from thousands to tens, providing a promising outlook for future functional analysis of these genes.

## Conclusions

The use of genome-wide transcriptome analysis highlights novel integrated molecular mechanisms associated with Cd-tolerance. Our results are potentially important for the characterisation of molecular mechanisms underlying Cd tolerance in barley. We demonstrated that Cd-tolerant Weisuobuzhi (1) is more capable to scavenge Cd-induced ROS; (2) is able to maintain ion homeostasis and sequester more Cd into the vacuoles *via* the ABC transporters and ATPase; (3) has higher efficiency in ET and Ca^2+^ signal transduction; (4) maintains normal cell wall function; and (5) expresses defence and DNA replication related proteins for Cd tolerance in barley. These distinct differences in gene expression profiles, biochemical and physiological functions between Cd-tolerant and -sensitive genotypes will provide critical information for extending our knowledge and guiding our future investigations into the candidate genes and proteins underlying Cd tolerance in barley.

## Electronic supplementary material

Additional file 1: Table S1: Name of genes and their primers used in quantitative RT-PCR. (PDF 66 KB)

Additional file 2: Figure S1: Leaf transcriptome profiles of Cd stress-responsive genes in barley leaves. (PDF 28 KB)

Additional file 3: Table S2: Summary of groups and numbers of differentially expressed genes between Cd-treated and control in leaves of two barley genotypes after exposure to 5 μM Cd for 15 d. (PDF 45 KB)

Additional file 4: Table S3: List of genes up-regulated in Weisuobuzhi and down-regulated in Dong17 after exposing the plants to 5 μM Cd for 15 d. (PDF 59 KB)

Additional file 5: Table S4: List of genes up-regulated in Weisuobuzhi and not changed in Dong17 after exposing the plants to 5 μM Cd for 15 d. (PDF 82 KB)

Additional file 6: Table S5: List of genes not changed in Weisuobuzhi and down-regulated in Dong17 after exposing the plants to 5 μM Cd for 15 d. (PDF 208 KB)

Additional file 7: Table S6: List of genes up-regulated in both Weisuobuzhi and Dong17 after exposing the plants to 5 μM Cd for 15 d. (PDF 96 KB)

Additional file 8: Table S7: List of genes down-regulated in Weisuobuzhi and up-regulated in Dong17 after exposing the plants to 5 μM Cd for 15 d. (PDF 70 KB)

Additional file 9: Table S8: List of genes down-regulated in both Weisuobuzhi and Dong17 after exposing the plants to 5 μM Cd for 15 d. (PDF 62 KB)

Additional file 10: Table S9: List of genes down-regulated in Weisuobuzhi and not changed in Dong17 after exposing the plants to 5 μM Cd for 15 d. (PDF 77 KB)

Additional file 11: Table S10: List of genes not changed in Weisuobuzhi and up-regulated Dong17 after exposing the plants to 5 μM Cd for 15 d. (PDF 185 KB)

Additional file 12: Figure S2: Functional categorisation and differential expression of Cd stress-regulated genes in barley leaves. (PDF 17 KB)

Additional file 13: Figure S3: Integrated schematic diagram of the mechanisms involved in vacuolar compartmentalization of Cd in barley leaves. (PDF 63 KB)

Additional file 14: Table S11: Links between the novel Cd-responsive genes in barley leaves from this study and Cd-responsive miRNAs and their putative targets from the literature. (PDF 14 KB)
