# Supplementary material for: Genome-wide transcriptome and functional analysis of two contrasting genotypes reveals key genes for cadmium tolerance in barley
Source: BMC Genomics. 2014 Jul 19;15(1):611. doi: 10.1186/1471-2164-15-611 (PMC4117959; doi:10.1186/1471-2164-15-611)
Supplement: Supplementary file 1 — Additional file 1: Table S1: Name of genes and their primers used in quantitative RT-PCR. (PDF 66 KB) [file 12864_2014_6304_MOESM1_ESM.pdf]

Additional File 1: Table S1 Name of genes and their primers used in quantitative RT-PCR.

Gene annotation	Forward primer（5’- 3’）	Reverse primer（5’- 3’）
Asparaginase	CACCGGCGAGGTGGCATACG	CGCGCACTTGCTCACTCCCA
C13 endopeptidase NP1 precursor	GAGTGAACCAGCGGGACGCC	CGCTCCTCGAACCTCCGCAC
P450	TGATGCTGGCCGTAATGTAC	ACGCAGAAGGAGTAGAGGAT
Nuclease I	ATCAAGCTGGAGCTGAGC	TCTTGCAGGTCATGTAGTGC
ATP synthase	ACACTCACAACGAATCACATG	AGCTCAGAGAATAGGCTCTTG
Catalase isozyme	GCTACGCTACTTCACTCAC	TCGTTGTCGTTCCACACC
Peroxidase	ACTTCCTTGCTGGTGCTCGTG	TGACGCCGCTCTTGATGGTG
Chitinase	TATTGCTAGGTTGGCATGATC	ATGGAACGCAACTCTCTCC
Lipid transfer protein	GAGAGGAGAGAACACGATGG	GTGTACTGGCACTTGTTGATC
ABC transporter family protein	AGTTAGCCAGGAGCCTACACTC	TAGCCGCGTTCTCAACGTC
Glutathione transferase	GATCTGCTTCTCTTCGAGTC	TGCTTCCTTCAGGTTGCC
Actin (Control)	TGGCTGACGGTGAGGACA	CGAGGGCGACCAACTATG
