# Supplementary material for: Genome-wide transcriptome and functional analysis of two contrasting genotypes reveals key genes for cadmium tolerance in barley
Source: BMC Genomics. 2014 Jul 19;15(1):611. doi: 10.1186/1471-2164-15-611 (PMC4117959; doi:10.1186/1471-2164-15-611)
Supplement: Supplementary file 2 — Additional file 2: Figure S1: Leaf transcriptome profiles of Cd stress-responsive genes in barley leaves. (PDF 28 KB) [file 12864_2014_6304_MOESM2_ESM.pdf]

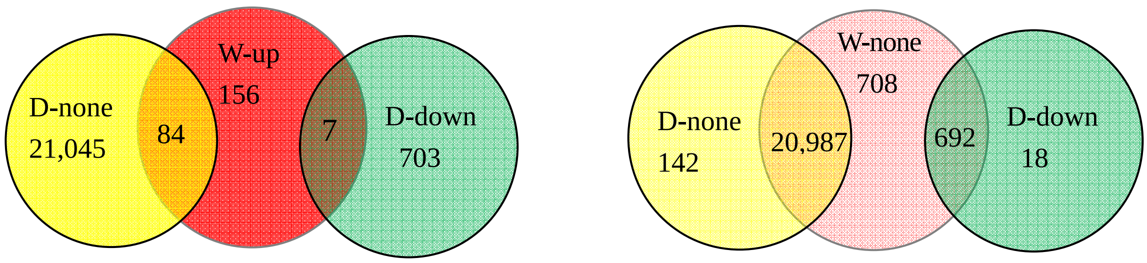

Additional File 1: Figure S1 Leaf transcriptome profiles of Cd stress-responsive genes in barley leaves. Venn diagrams show the number of genes regulated by Cd treatment (5 µM Cd treatment for 15 d) and overlap between the two genotypes: Weisuobuzhi (W) and Dong17 (D). Number of genes for which transcript abundance increased (up), decreased (down) by >2-fold and no change (none) are shown in comparison to the control.
