# Supplementary material for: Genome-wide transcriptome and functional analysis of two contrasting genotypes reveals key genes for cadmium tolerance in barley
Source: BMC Genomics. 2014 Jul 19;15(1):611. doi: 10.1186/1471-2164-15-611 (PMC4117959; doi:10.1186/1471-2164-15-611)
Supplement: Supplementary file 3 — Additional file 3: Table S2: Summary of groups and numbers of differentially expressed genes between Cd-treated and control in leaves of two barley genotypes after exposure to 5 μM Cd for 15 d. (PDF 45 KB) [file 12864_2014_6304_MOESM3_ESM.pdf]

Additional File 3: Table S2 Summary of groups and numbers of differentially expressed genes between Cd-treated and control in leaves of two barley genotypes after exposure to 5 μM Cd for 15 d.

Group	Cd *vs* Control		Known	Unknown	Total	%
	Weisuobuzhi	Dong 17				
I	Up	Down	2	5	7	0.031
II	Up	Non	64	20	84	0.369
III	Non	Down	451	241	692	3.043
IV	Up	Up	119	37	156	0.686
V	Down	Up	30	4	34	0.150
VI	Down	Down	10	1	11	0.048
VII	Down	Non	50	8	58	0.255
VIII	Non	Up	413	295	708	3.114
IX	Non	Non	15693	5294	20987	92.303
