# Supplementary material for: Genome-wide transcriptome and functional analysis of two contrasting genotypes reveals key genes for cadmium tolerance in barley
Source: BMC Genomics. 2014 Jul 19;15(1):611. doi: 10.1186/1471-2164-15-611 (PMC4117959; doi:10.1186/1471-2164-15-611)
Supplement: Supplementary file 4 — Additional file 4: Table S3: List of genes up-regulated in Weisuobuzhi and down-regulated in Dong17 after exposing the plants to 5 μM Cd for 15 d. (PDF 59 KB) [file 12864_2014_6304_MOESM4_ESM.pdf]

Additional File 4: Table S3 List of genes up-regulated in Weisuobuzhi and down-regulated in Dong17 after exposing the plants to 5 μM Cd for 15 d.

Annotation	Probe Set ID	Fold change* (Cd *vs* control)		Accession No	E-value
		Weisuobuzhi	Dong17		
**Protein synthesis**					
Asparaginase [*H. vulgare*]	Contig8740_at	2.39	-2.15	AAG28786.1	2e-71
C13 endopeptidase NP1 precursor [*Zea mays*]	Contig2783_s_at	2.42	-2.48	AAD04883.1	3e-53
**None**					
None	HVSMEg0002G01r2_at	20.63	-11.55	none	none
None	HS06A14u_s_at	4.15	-6.15	none	none
None	Contig2279_s_at	2.4	-4.41	none	none
None	Contig2279_at	2.32	-3.38	none	none
None	EBpi03_SQ001_J13_at	2.06	-2.12	none	none

* The fold change represents the mean ratio of gene expression in leaves of the two genotypes exposed to 5 μM Cd for 15 d over those in the control. Genes were considered up-regulated and down-regulated if the induction ratio was >2.0 and <-2.0, respectively.
