# Supplementary material for: Genome-wide transcriptome and functional analysis of two contrasting genotypes reveals key genes for cadmium tolerance in barley
Source: BMC Genomics. 2014 Jul 19;15(1):611. doi: 10.1186/1471-2164-15-611 (PMC4117959; doi:10.1186/1471-2164-15-611)
Supplement: Supplementary file 5 — Additional file 5: Table S4: List of genes up-regulated in Weisuobuzhi and not changed in Dong17 after exposing the plants to 5 μM Cd for 15 d. (PDF 82 KB) [file 12864_2014_6304_MOESM5_ESM.pdf]

Additional File 5: Table S4 List of genes up-regulated in Weisuobuzhi and not changed in Dong17 after exposing the plants to 5 μM Cd for 15 d.

Annotation	Probe Set ID	Fold change* (Cd *vs* Control)		Accession No	E-value
		W	D		
**Stress and defense response**					
P450 [Triticum aestivum]	Contig11957_at	2.36	-1.33	BAB87820.1	1e-60
Nuclease I [*H. vulgare*]	Contig4111_at	2.05	-1.32	BAA82696.1	e-160
Defensin [*T. aestivum*]	Contig6933_s_at	2.01	-1.06	BAC10287.1	4e-25
Drought inducible 22 kD protein [*Saccharum officinarum*]	Contig2320_s_at	2.67	1.06	BAB68268.1	7e-09
Catalase isozyme 2 pir [*H. vulgare*]	HT06F11u_s_at	2.2	1.06	P55308	1e-32
Probable peroxidase [*Spinacia oleracea*]	Contig3243_x_at	2.75	1.11	T09166	1e-79
Probable peroxidase [*H. vulgare*]	Contig1852_at	2.04	1.29	T04454	e-106
Senescence-associated protein 15 [*Hemerocallis sp.* (Daylily)]	Contig6411_at	2.2	1.78	AAC34858.1	2e-96
Chitinase 2 [*T. aestivum*]	Contig3574_s_at	2.14	1.79	BAB82472.1	2e-51
1-aminocyclopropane-1-carboxylate oxidase [*Phyllostachys edulis*]	rbasd24g02_s_at	2.17	1.86	BAB32502.1	2e-40
Putative cytochrome P450 [*Oryza sativa*]	Contig14534_at	2.64	1.94	AAL73064.1	1e-44
**Transport**					
Vacuolar ATP synthase 16 kDa proteolipid subunit [*Homo sapiens*]	HA10P21u_at	3.52	1.05	Q40635	2e-29
Nucleoside-diphosphate kinase I [*O. sativa*]	Contig8936_at	2.07	1.13	Q07661	1e-75
Endoxyloglucan transferase [*H. vulgare*]	Contig5258_at	2.26	1.22	CAA62847.1	e-168
Beta-tubulin 6 [*T. aestivum*]	Contig1511_s_at	2.1	1.32	AAD10493.1	e-123
OSJNBa0060B20.12 [*O. sativa*]	Contig10142_at	2.39	1.35	CAD39778.1	2e-95
Physical impedance induced protein [*Z. mays*]	Contig3783_s_at	2.78	1.4	AAC31615.1	2e-17
Putative lipid transfer protein [*O. sativa*]	Contig4414_at	2.58	1.69	AAN05565.1	3e-25
Physical impedance induced protein [*Z. mays*]	Contig3783_at	2.91	1.9	AAC31615.1	2e-17
Mitochondrial carrier protein family [*Arabidopsis thaliana*]	Contig6912_s_at	2.07	1.9	NP_568060.1	1e-27
Putative glutamate/ornithine acetyltransferase [*A. thaliana*]	HVSMEn0001P19r2_at	8.08	1.91	NP_565863.1	7e-06
**Transcription**					
OSJNBb0091E11.14 [*O. sativa*]	HS17I17u_s_at	3.09	1.39	CAD41545.1	0.031
Nucleoid DNA-binding protein [*O. sativa*]	HV_CEb0010H17r2_at	2.58	1.59	BAB63755.1	3e-12
Putative MAR-binding protein MFP1 [*O. sativa*]	Contig13824_at	2.22	1.85	BAB33019.1	3e-34
**Carbohydrate metabolism**					
Endo-1,4-beta-glucanase [*H. vulgare*]	Contig4147_at	2.13	-1.47	BAA94257.1	e-135
Putative cellulase [*O. sativa*]	Contig1616_at	2.09	1.22	AAK20055.1	2e-92
Xyloglucan endo-1,4-beta-D-glucanase [*H. vulgare*]	Contig2671_at	2.32	1.38	T06202	e-160
Putative pectinacetylesterase [*O. sativa*]	Contig9967_at	2.91	1.44	BAB90194.1	3e-48
Putative beta-glucosidase [*O. sativa*]	rbah29m06_s_at	2.13	1.48	BAB90397.1	6e-47
Apoplastic invertase [*O. sativa*]	Contig4470_s_at	2.82	1.83	AAD38399.1	1e-94
Putative acyl-CoA synthetase [*A. thaliana*]	Contig4658_at	2.15	1.87	NP_182246.1	8e-83
**Fat metabolism**					
Putative beta-ketoacyl-CoA synthase [*A. thaliana*]	Contig15532_at	2.11	1.45	NP_171918.1	5e-75
Very-long-chain fatty acid condensing enzyme CUT1 [*A. thaliana*]	Contig18311_at	2.57	1.78	AAM65060.1	3e-75
Fiddlehead-like protein [Gossypium hirsutum]	Contig4206_at	2.38	1.81	AAL67993.1	8e-85
**Photosynthesis**					
Photosystem II 10 kDa polypeptide [*H. vulgare*]	Contig996_s_at	8.89	-1.1	Q40070	3e-44
Macrophage migration inhibitory factor family [*A. thaliana*]	Contig15181_at	2.06	1.33	NP_200527.1	8e-37
**Protein synthesis**					
Asparaginase [*H. vulgare*]	Contig8739_at	2.24	-1.16	AAG28786.1	2e-99
Putative L-allo-threonine aldolase [*A. thaliana*]	Contig16415_at	2.2	-1.05	AAF63783.1	5e-71
Glutamine-dependent asparagine synthetase 1 [*H. vulgare*]	HV11O04r_at	3.05	0	AAK49456.1	5e-53
**Signal transduction**					
23 KD jasmonate-induced protein 1 [*H. vulgare*]	Contig1686_at	2.17	1.17	P32024	5e-29
Phi-1 [Nicotiana tabacum]	Contig6582_at	2.08	1.2	BAA33810.1	4e-59
HtrA-like serine protease [*Clostridium acetobutylicum*]	Contig3305_at	2.46	1.24	NP_349047.1	3e-07
Putative diacylglycerol kinase [*O. sativa*]	Contig20753_at	2.1	1.42	BAB92552.1	e-104
Phi-1 [*N. tabacum*]	HVSMEg0002E24r2_s_at	2.58	1.68	BAA33810.1	8e-04
Phosphate-induced protein [*Pennisetum ciliare*]	Contig9813_at	2.05	-1.28	AAK15505.1	5e-59
**Unknown classified**					
Expressed protein [*A. thaliana*]	Contig852_at	2.36	1.68	NP_567420.1	8e-40
Hypothetical protein [*O. sativa*]	Contig9843_s_at	2.28	-1.7	BAC15985.1	1e-34
Unnamed protein product [*O. sativa*]	Contig22781_at	2.32	-1.45	BAA96221.1	3e-05

Putative protein [*A. thaliana*]	Contig11345_at	2.27	-1.41	NP_567717.1	2e-24
P0045F02.11 [*O. sativa*]	Contig25762_at	2.64	-1.38	BAC16424.1	4e-22
Hypothetical protein [*O. sativa*]	HW05D06V_at	2.02	1.04	BAB67859.1	5e-18
Hypothetical protein [*O. sativa*]	Contig9058_at	3.55	1.05	BAB86120.1	2e-49
Putative uncharacterized protein [*O. sativa*]	Contig2474_at	2.15	1.15	BAB21293.1	e-128
Unknown [*H. sapiens*]	Contig3024_at	2.08	1.15	AAH29062.1	7e-84
Hypothetical protein [*O. sativa*]	Contig7849_at	2.06	1.19	BAB92157.1	e-102
Correspond to a region of the predicted gene. [*O. sativa*]	Contig8760_at	2.21	1.3	BAA90810.1	2e-71
Hypothetical protein [*O. sativa*]	Contig5827_at	2.1	1.34	BAC15948.1	1e-76
Unknown protein [*O. sativa*]	Contig15559_at	2.03	1.58	BAB86220.1	2e-67
Hypothetical protein [*A. thaliana*]	Contig9199_at	2	1.6	NP_171639.1	3e-17
Hypothetical protein [*O. sativa*]	Contig9844_at	2.15	1.7	BAC15985.1	7e-33
Hypothetical protein [*O. sativa*]	Contig6925_at	2.3	1.92	BAB16875.1	2e-58
Hypothetical protein [*Sorghum bicolor*]	Contig10822_at	2.16	1.93	AAL73975.1	2e-68
Putative nodulin [*O. sativa*]	Contig10919_s_at	2.15	1.96	BAB17350.1	5e-29
Expressed protein [*A. thaliana*]	Contig11690_at	2.34	1.96	NP_564433.1	7e-77
**None**					
none	EBan01_SQ002_N10_x_at	3.2	-1.08	none	none
none	Contig8729_at	2.24	1.07	none	none
none	Contig16913_at	4.07	1.11	none	none
none	Contig4391_at	2.56	1.15	none	none
none	HVSMEm0002P02r2_x_at	3.37	1.16	none	none
none	Contig13977_at	2.23	1.23	none	none
none	Contig9248_s_at	3.13	1.23	none	none
none	Contig7315_at	2.13	1.25	none	none
none	Contig4413_s_at	2.12	1.42	none	none
none	Contig17275_at	2.32	1.46	none	none
none	HVSMEf0023D17f_s_at	2.26	1.52	none	none
none	HS18B10u_s_at	2.13	1.53	none	none
none	S0000800242C04F1_at	2.7	1.57	none	none
none	HV_CEa0009O11f2_x_at	2.04	1.69	none	none
none	Contig2496_at	2.49	1.76	none	none
none	HS06J15u_x_at	4.8	1.78	none	none
none	Contig6699_s_at	2.11	1.83	none	none
none	Contig11003_s_at	2.19	1.91	none	none
none	HS05F12u_s_at	2.21	1.92	none	none
none	HA13E08r_s_at	2.19	1.97	none	none

* The fold change represents the mean ratio of gene expression in leaves of the two genotypes exposed to 5 μM Cd for 15 d over those in the control. Genes were considered up-regulated and down-regulated if the induction ratio was >2.0 and <-2.0, respectively.
