# Supplementary material for: Genome-wide transcriptome and functional analysis of two contrasting genotypes reveals key genes for cadmium tolerance in barley
Source: BMC Genomics. 2014 Jul 19;15(1):611. doi: 10.1186/1471-2164-15-611 (PMC4117959; doi:10.1186/1471-2164-15-611)
Supplement: Supplementary file 6 — Additional file 6: Table S5: List of genes not changed in Weisuobuzhi and down-regulated in Dong17 after exposing the plants to 5 μM Cd for 15 d. (PDF 208 KB) [file 12864_2014_6304_MOESM6_ESM.pdf]

Additional File 6: Table S5 List of genes not changed in Weisuobuzhi and down-regulated in Dong17 after exposing the plants to 5 μM Cd for 15 d.

Annotation	Probe Set ID	Fold change (Cd *vs* control)		AccessionNo	E-value
		WS	D		
**Stress and defense response**					
Peroxidase [*H. vulgare*]	Contig1868_s_at	-1.32	-16.39	CAA37464.1	2e-92
Sporozoite surface protein 2 [*Plasmodium yoelii*]	HC107A09_SK_at	1.12	-6.64	A60540	0.011
Resistance gene analog PIC23 [*O. sativa*]	AF146270_at	1.1	-6.53	AAF21363.1	8e-94
Autophagocytosis protein-like [*A. thaliana*]	Contig4358_at	1.21	-4.82	AAM66117.1	6e-10
TRI16 [Fusarium sporotrichioides]	HVSMEl0013I05r2_at	1.03	-4.65	AAG47841.1	5e-52
Ferritin [*O. sativa* (japonica)]	HV12A05u_s_at	-1.62	-4.26	AAM74943.1	4e-34
Ferritin 1, chloroplast precursor [*Z. mays*]	Contig2714_at	-1.49	-4.06	P29036	3e-71
Putative calmodulin-like protein [*O. sativa*]	Contig25034_at	1.06	-3.8	AAK52557.1	7e-13
9-cis-epoxycarotenoid dioxygenase [*A. thaliana*]	Contig4988_at	1.06	-3.79	NP_193652.1	3e-71
Similar to copper chaperone related protein [*O. sativa*]	Contig13380_at	-1.12	-3.62	BAC15988.1	1e-09
Heat shock protein-like [*O. sativa* (japonica)]	Contig12376_at	-1.88	-3.47	BAB84833.1	2e-53
Similar to A. thaliana chromosome 5, MQK4.27 [*O. sativa* (japonica)]	Contig11181_at	-1.16	-3.35	BAB62620.1	e-103
Putative NADPH:quinone oxidoreductase [*O. sativa* (japonica)]	Contig9968_at	-1.27	-3.32	BAB64708.1	3e-69
Putative peroxidase [*O. sativa* (japonica)]	Contig17906_at	-1.03	-3.02	BAC16194.1	9e-25
Inwardly rectifying potassium channel [*Z. mays*]	HV_CEb0016L15r2_at	1.04	-3.02	CAD18901.1	4e-29
Nucleolar autoantigen like protein [*A. thaliana*]	Contig21874_at	-1.04	-3.01	NP_191609.1	5e-11
Ferritin [*O. sativa* (japonica)]	Contig2716_s_at	-1.64	-2.97	AAM74942.1	3e-79
Hordoindoline-b [H. vulgare subsp. vulgare]	Contig231_x_at	-1.02	-2.96	CAB92291.2	5e-68
Ferredoxin-sulfite reductase precursor [*Z. mays*]	Contig4569_at	-1.51	-2.95	T01695	e-125
Putative heat shock protein [*O. sativa*]	Contig17190_at	-1.35	-2.74	AAL83988.1	2e-36
Dynein light chain, outer arm [*Chlamydomonas reinhardtii*]	EBro08_SQ011_H09_at	1.02	-2.73	Q39584	3e-07
Globulin-2 precursor [*Z. mays*]	Contig2027_x_at	1.08	-2.7	S15675	5e-20
(AB026439) Ntdin [*N. tabacum*]	Contig22898_at	-1.64	-2.65	BAA88985.2	7e-09
Probable peroxidase [*S. oleracea*]	Contig3241_at	1.99	-2.64	T09164	5e-47
Putative CuZn-superoxide dismutase [*Populus tremula x Populus tremuloides*]	HVSMEl0013E18r2_at	1.03	-2.58	CAC33846.2	2e-35
Putative glutathione-dependent formaldehyde dehydrogenase [*S. pombe*]	HV01H04w_at	-1.01	-2.54	NP_595070.1	2e-17
(AF181452) dehydrin; Dhn2 [*H. vulgare*]	Contig1721_at	-1.08	-2.48	AAF01690.1	6e-37
Blue copper-binding protein homolog [*T. aestivum*]	Contig3209_s_at	-1.08	-2.47	AAD10251.1	1e-39
Os01g0858350 protein [*O. sativa*]	HI05D22u_at	-1.3	-2.43	BAB63711.1	4e-45
Pathogen-induced protein WIR1A [*T. aestivum*]	Contig6520_at	1.03	-2.42	Q01482	3e-09
(AY013869) antigen 38 [*Trypanosoma cruzi*]	Contig20307_at	-1.23	-2.41	AAG46367.1	5e-07
Carbonyl reductase like protein [*O. sativa* (japonica)]	HVSMEa0014B08r2_at	-1.14	-2.4	BAC10339.1	2e-26
Resistance gene analog PIC25 [*H. vulgare*]	AF146272_s_at	1.04	-2.36	AAF21365.1	7e-84
Putative disease resistance gene [*O. sativa* (japonica)]	Contig15571_at	1.14	-2.33	AAN08649.1	7e-05
P0700A11.20 [*O. sativa* (japonica)]	Contig25831_at	-1.22	-2.31	BAB89919.1	0.032
Blue copper-binding protein homolog [*T. aestivum*]	Contig3211_at	-1.08	-2.26	AAD10251.1	5e-41
Probable enoyl-reductase (NADH2) [*O. sativa*]	Contig4009_at	1.07	-2.25	T03735	e-109
Putative Cf2/Cf5 disease resistance protein [*O. sativa* (japonica)]	Contig13517_at	-1.1	-2.24	BAB89966.1	7e-32
Small heat shock protein HSP17.8 [*T. aestivum*]	Contig3288_x_at	-1.23	-2.24	AAK51797.1	2e-64
Putative far-red impaired response protein [*O. sativa* (japonica)]	Contig14561_at	-1.29	-2.22	BAC15777.1	3e-19
Thionin precursor, leaf [*H. vulgare*]	Contig1583_at	1.29	-2.22	S22515	1e-43
(AL158821) dJ75H8.2 [*H. sapiens*]	Contig22572_at	1.1	-2.21	CAD23056.1	9e-12
Similar to HSPC049 protein [*H. sapiens*]	Contig23071_at	-1	-2.21	XP_050288.3	1e-10
Probable profilin PRO1 [*T. aestivum*]	HVSMEi0012J12r2_at	1.04	-2.21	P49232	2e-11
Oxidase [T. aestivum]	Contig5780_at	1.2	-2.2	AAG00450.1	3e-98
Putative gibberellin oxidase [*O. sativa* (japonica)]	Contig7692_s_at	-1.27	-2.19	AAM92286.1	5e-70
(AC082644) putative peroxidase [*O. sativa*]	Contig14609_at	1.1	-2.18	AAG46133.1	1e-95
Avr9/Cf-9 rapidly elicited protein 194 [*N. tabacum*]	Contig20048_at	1.02	-2.18	AAG43553.1	1e-08
Putative uncharacterized protein At4g33540 [*A. thaliana*]	HB19M13r_at	1.13	-2.18	NP_195080.2	5e-10
P0468H06.6 [*O. sativa* (japonica)]	HW09C07u_at	-1.02	-2.17	BAB55695.1	2e-14
(NM_106112) AtHVA22a [*A. thaliana*]	Contig11977_at	-1.51	-2.16	NP_177592.1	4e-60
(AF486634) ferredoxin precursor [*T. aestivum*]	Contig5299_at	-1.59	-2.15	AAL92109.1	3e-75
Cytochrome c pir||CCWT cytochrome c[*T. aestivum*]	HS05N09u_s_at	-1.22	-2.14	P00068	2e-11
Similar to chitinase (AF013581) [*O. sativa* (japonica)]	HVSMEf0020E19r2_at	-1.05	-2.14	BAA95846.1	2e-24
Putative bacterial blight resistance protein Xa1-like [*O. sativa*]	Contig14071_at	1.24	-2.12	AAK52518.1	1e-11

Bacterial-induced peroxidase precursor [*G. hirsutum*]	Contig3239_at	1.81	-2.12	AAD43561.1	5e-58
Germin-like protein [*H. vulgare*]	Contig3156_s_at	-1.15	-2.07	T05956	7e-36
Gamma-thionin [Petunia integrifolia]	EBes01_SQ004_M23_at	-1.22	-2.06	Q40901	0.001
Protein phosphatase 2C (PP2C) [*A. thaliana*]	Contig22065_at	-1.09	-2.05	NP_187748.1	6e-22
Similar to mRNA for zinc-finger protein [*O. sativa* (japonica)]	Contig10709_at	1.41	-2.03	BAA90806.1	5e-44
Alpha-N-acetylglucosaminidase [*N. tabacum*]	Contig20555_at	1.02	-2.03	CAA77084.1	3e-21
Expressed protein; protein id: At1g29690.1 [*A. thaliana*]	Contig13528_at	1.03	-2.02	NP_564333.1	5e-76
Probable protein phosphatase 2C 40 [*O. sativa* (japonica)]	HVSMEl0010B09r2_at	-1.18	-2.02	CAD40875.1	2e-53
Putative dynein light chain [*O.sativa* (japonica)]	Contig16592_at	1.22	-2	BAB61206.1	2e-41
**Transport**					
T03297 lipid transfer protein precursor [*O.sativa*]	Contig2041_at	-1.13	-8.64	Q42976	3e-33
Putative DNA cytosine methyltransferase Zmet3 [*Z. mays*]	EBes01_SQ002_J08_at	1.22	-8.34	AAF68437.1	1e-28
Putative transferase-like protein [*Musa acuminata*]	Contig22112_at	-1.03	-6.19	AAL73122.1	4e-44
OSJNBa0086B14.13 [*O. sativa* (japonica)]	EBro02_SQ001_G03_at	-1.16	-6.04	CAD40841.1	8e-35
Putativeo-methyltransferase ZRP4 [*O. sativa*]	Contig8812_x_at	-1.77	-4.57	AAL31649.1	1e-61
Contains a weak similarity to a farnesylated protein [*A. thaliana*]	Contig11454_at	-1.06	-4.44	AAF81309.1	8e-20
ABC transporter family protein [*A. thaliana*]	Contig5297_at	-1.09	-4.34	NP_176636.1	2e-57
Putative vesicle transfer ATPase [*A. thaliana*]	HVSMEg0007D18r2_at	1.36	-4.2	NP_192327.1	8e-10
Putative salicylate-induced glucosyltransferase [*O. sativa* (japonica)]	HM03J24u_at	1.02	-3.67	BAB63778.1	4e-08
putative protein; protein id: At5g15240.1 [*A. thaliana*]	HVSMEg0017A08r2_at	-1.51	-3.6	NP_197028.1	9e-06
Sodium-dicarboxylate cotransporter-like [*A. thaliana*]	rbaal41j07_at	1.09	-3.54	NP_199567.1	3e-32
Glycosyl transferase, putative [*A. thaliana*]	Contig11018_at	1.7	-3.38	NP_189150.1	e-117
Apocytochrome b (aa 1-498) [*T. aestivum*]	rbah59d21_at	1.2	-3.21	CAA26207.1	1e-18
O-diphenol-O-methyl transferase, putative At3g53140.1 [*A. thaliana*]	Contig14539_at	1	-2.92	NP_190882.1	1e-40
Clathrin-associated protein, putative [*A. thaliana*]	Contig9665_at	-1.25	-2.91	NP_176052.2	1e-52
Putative thioredoxin-like 1 [*A. thaliana*]	Contig5236_at	1	-2.9	NP_172333.1	3e-67
Glycosyl transferase, putative [*A. thaliana*]	HV_CEa0015H07r2_s_at	-1.05	-2.88	NP_189150.1	1e-19
Herbicide safener binding protein 1 [*Z. mays*]	Contig5613_at	-1.03	-2.85	T01354	5e-45
(AE003845) CG32000-PA [*Drosophila melanogaster*]	HVSMEi0002M06r2_at	1.2	-2.82	AAN06554.1	1e-05
Putative uncharacterized protein F28M20.210 [*A. thaliana*]	Contig12258_at	-1.04	-2.71	NP_567879.1	2e-67
Putative anion exchange protein [*Schistosoma mansoni*]	Contig19634_at	-1.3	-2.58	NP_182238.2	1e-46
ADP-glucose pyrophosphorylase [*Cicer arietinum*]	HVSMEi0002I05r2_at	-1.11	-2.56	AAK27718.1	3e-22
(AF140606) nitrate transporter [*O. sativa*]	Contig23379_at	1.2	-2.53	AAF07875.1	6e-91
Glutathione transferase F4 [*T. aestivum*]	Contig6238_s_at	-1.32	-2.52	CAD29477.1	2e-98
F18O14.34 [*A. thaliana*]	HS17M23u_at	1.5	-2.48	AAF79435.1	4e-22
Similar to tRNA isopentenyltransferase [*O. sativa* (japonica)]	Contig15042_at	-1.25	-2.43	BAB85325.1	2e-65
T03297 lipid transfer protein precursor [*O. sativa*]	Contig2041_x_at	1.11	-2.39	Q42976	3e-33
Nitrite transport protein, chloroplast [*Cucumis sativus*]	Contig21251_at	1.11	-2.39	T10255	1e-29
Integral membrane protein, putative [*A. thaliana*]	Contig20064_at	1.23	-2.38	NP_189291.1	3e-22
Catechol O-methyltransferase [*H. vulgare*]	HVSMEl0007E24r2_at	1.07	-2.31	S52015	2e-28
Rev interacting protein-like [*O. sativa* (japonica)]	Contig21528_at	1.26	-2.3	BAB89130.1	1e-14
Putative heme A farnesyltransferase [*O. sativa* (japonica)]	AJ222781_at	1.05	-2.28	BAB90120.1	3e-19
Putative DNA methyltransferase [*O. sativa* (japonica)]	HF22L15r_s_at	-1.11	-2.26	BAB93271.1	1e-07
Similarto hexose carrierprotein [*O. sativa* (japonica)]	HVSMEa0008H21r2_at	1.99	-2.25	BAA83554.1	3e-23
Hypothetical protein A_IG002N01.8 [*A. thaliana*]	Contig17032_at	1.03	-2.24	T01721	7e-29
(AL606632) OSJNBa0042L16.10 [*O. sativa* (japonica)]	Contig18750_at	-1.15	-2.24	CAD41012.1	2e-23
Ceramide glucosyltransferase [*Magnaporthe grisea*]	HT07G22u_at	1.26	-2.24	AAK73019.1	9e-05
7 kDa lipid transfer protein [*H. vulgare*]	Contig150_at	-1.27	-2.23	T04395	3e-40
Putative membrane protein [*A. thaliana*]	Contig25699_at	-1.06	-2.21	NP_178286.1	3e-06
Putative glucosyltransferase [*C. arietinum*]	HF22I06r_at	-1.29	-2.16	CAB88664.1	2e-10
Putative anthocyanin 5-O-glucosyltransferase [*O. sativa* (japonica)]	Contig10670_at	1.03	-2.14	BAB07962.1	2e-57
At1g47530/F16N3_20 [*A. thaliana*]	HVSMEn0015M01r2_at	1.13	-2.14	AAL24258.1	3e-08
Putative glucosyltransferase [*A. thaliana*]	Contig22412_at	-1	-2.13	NP_181234.1	2e-32
Putative farnesyltranstransferase [O. sativa (japonica)]	HV_CEb0002K12r2_x_at	-1.46	-2.09	BAB89459.1	1e-14
Sodium-dicarboxylate cotransporter-like [*A. thaliana*]	Contig17624_at	1.09	-2.08	NP_199567.1	4e-69
ABC transporter family protein [*A. thaliana*]	Contig5296_at	1.01	-2.08	NP_176636.1	e-121
Coatomer delta subunit [O. sativa]	Contig8209_at	-1.06	-2.07	P49661	1e-06
Uroporphyrin-III C-methyltransferase [*O. sativa* (japonica)]	Contig10779_at	-1.93	-2.06	BAB89483.1	6e-98
Putative tonoplast membrane integral protein [*O. sativa* (japonica)]	HVSMEf0019H18r2_at	-1.33	-2.06	BAB63833.1	3e-32
Glutathione transferase F3 [*T. aestivum*]	HW09A20u_at	-1.42	-2.02	CAD29476.1	1e-09
Beta 1,2-xylosyltransferase [*A. thaliana*]	Contig18221_at	-1.19	-2.02	AAF77064.1	9e-80
Putative sugar transporter [*O. sativa*]	HY05O16u_at	-1.07	-2.02	AAG46115.1	2e-07
Acetylglucosaminyl-phosphatidylinositol[*O. sativa* (japonica)]	Contig8470_s_at	1.06	-2.1		e-114

**Transcription**					
Proliferating cell nuclear antigen II [*N. tabacum*]	HVSMEm0021F22r2_at	-1.03	-7.8	AAD19905.1	5e-18
Histone H2A.2 [*T. aestivum*]	Contig869_x_at	-1.01	-6.12	S53518	1e-46
CBF-like protein [*S. cereale*]	HVSMEn0019L21f_at	-1.23	-4.24	AAL35760.1	5e-37
Contains similarity to RNA adenosine deaminase gene [*A. thaliana*]	Contig8667_at	-1.46	-4.05	AAF78409.1	2e-13
Histone H2A [Petroselinum crispum]	Contig414_x_at	1.13	-4.04	P19177	4e-43
Putative choline kinase P0489A01.8 [*O. sativa* (japonica)]	Contig11743_at	-1.02	-3.97	BAA99517.1	4e-98
Putative receptor protein kinase [*O. sativa* (japonica)]	Contig18956_at	-1.3	-3.88	BAC06203.1	1e-15
Similar to nucleoid DNA-binding-like protein [*O. sativa* (japonica)]	Contig13984_at	1	-3.41	BAC15479.1	3e-96
Putative protein kinase Xa21 [*O. sativa* (japonica)]	Contig19845_at	-1.18	-3.35	BAC10827.1	1e-45
Histone H4 [*T. aestivum*]	Contig122_at	-1.49	-3.34	HSWT41	5e-42
Putative RNA-binding protein [*O. sativa* (japonica)]	Contig5789_at	-1.09	-3.32	AAM92828.1	6e-71
Membrane related protein CP5, putative [*A. thaliana*]	Contig3339_at	-1.07	-3.31	AAM91533.1	1e-82
RNase S-like protein [*H. vulgare*]	Contig5058_x_at	1.27	-3.31	AAM80567.1	2e-53
Putative ethylene responsive element binding factor [*O. sativa*]	Contig17873_at	-1.06	-3.3	CAC39060.1	5e-29
Myb family transcription factor [*A. thaliana*]	Contig8132_s_at	-1.36	-3.24	NP_190199.1	5e-46
Histone H3 (clone pH3c-1) [*A. thaliana*]	Contig338_s_at	-1.17	-3.21	P02300	7e-70
DNA-binding protein 2 [*N. tabacum*]	Contig17247_at	1.11	-3.2	AAD16139.1	9e-05
AT3g52500/F22O6_120 [*A. thaliana*]	Contig26235_at	1.22	-3.14	AAL14384.1	7e-04
Putative ubiquitin-conjugating protein [*O. sativa*]	Contig8462_at	1.32	-3.13	AAK26123.1	1e-56
CiFT protein [*Citrus unshiu*]	Contig17914_at	-1.55	-3.03	BAA77836.1	4e-57
Histone H2A.3 [*T. aestivum*]	HI04A06u_at	1.02	-3.03	S53520	7e-16
Putative polyprotein [*O. sativa*]	HV_CEa0010G01r2_at	1.17	-2.95	AAL31045.1	1e-22
Pirin-like protein [Lycopersicon esculentum]	HV03G03u_at	-1.49	-2.95	Q9SEE4	3e-37
Putative basic protein [*O. sativa*]	Contig7990_at	-1.12	-2.93	AAK38502.1	4e-36
Similar to nonhistone chromosomal protein [*M. musculus*]	HVSMEc0003G09f_x_at	-1.73	-2.93	XP_140242.1	4
At2g17400 [*A. thaliana*]	Contig12950_at	-1.36	-2.76	AAK96550.1	7e-55
Similar to late embryogenesis abundant proteins[*A. thaliana*]	Contig5269_x_at	-1.06	-2.75	NP_181073.1	4e-04
Putative dna repair protein with XPA domain; yeast rad14 homolog [*S. pombe*]	HVSMEm0023B10 r2_at	1.38	-2.74	NP_595222.1	2e-10
rRNA 2'-O-methyltransferase fibrillarin 1 [*A. thaliana*]	Contig3454_at	1.14	-2.72	AAF00542.1	1e-33
Putative ubiquitin-specific protein [O. sativa (japonica)]	Contig4602_at	1.15	-2.68	BAB90685.1	e-113
Tubulin beta-1 chain [*T. aestivum*]	Contig1508_s_at	-1.07	-2.67	Q9ZRB2	e-121
Histone H2B pir [*T. aestivum*]	Contig1136_at	-1.45	-2.65	P27807	8e-46
Putative calmodulin-binding protein [*A. thaliana*]	Contig14291_at	1.11	-2.63	T04234	3e-18
DNA polymerase alpha catalytic subunit [*O. sativa*]	Contig12726_at	1.07	-2.62	O48653	e-108
CBF1-like protein BCBF1 [*H. vulgare*]	Contig15617_at	-1.02	-2.6	AAK01088.1	7e-96
Origin recognition complex subunit 4 [*Z. mays*]	HC111D12_T3_at	-1.4	-2.6	AAL10455.1	1e-10
Related-to-ubiquitin (AtRUB1), putative [*A. thaliana*]	S0000200014F04F1_at	-1.14	-2.6	NP_564379.1	2e-16
Putative purine permease [*O. sativa* (japonica)]	Contig15320_at	1.3	-2.57	BAB90042.1	2e-44
Histone H3 pir [*A. thaliana*]	Contig657_s_at	1.15	-2.55	P02300	5e-69
Alanyl-tRNA synthetase [*Thermus thermophilus*]	HC109H12_T3_at	-1.02	-2.53	P74941	6e-08
Heat shock transcription factor [*Glycine max*]	Contig18148_at	-1.32	-2.51	S59541	1e-14
Putative rice retrotransposon retrofit gag/pol polyprotein [*O. sativa* (japonica)]	Contig26013_at	-1.32	-2.49	BAB90546.1	2e-50
AP2-containing protein [*T. aestivum*]	Contig9961_at	-1.06	-2.48	AAL01124.1	8e-98
Probable small nuclear ribonucleoprotein D2 [*A. thaliana*]	Contig4837_at	-1.07	-2.46	T00420	4e-45
Putative receptor protein kinase [*S. bicolor*]	Contig18966_at	1.02	-2.45	AAL68842.1	4e-10
Putative RNA apurinic site specific lyase[*O. sativa* (japonica)]	Contig19093_s_at	-1.05	-2.45	BAC16431.1	3e-07
DNA topoisomerase 6 subunit A [*A. thaliana*]	Contig9947_at	1.09	-2.45	NP_195902.1	6e-95
Putative transcription binding factor [*O. sativa* (japonica)]	Contig7380_at	1.28	-2.44	AAM93459.1	8e-30
Genomic DNA, chromosome 5, TAC clone: K9I9 [*A. thaliana*]	Contig12198_at	-1.18	-2.42	NP_201549.1	6e-04
Lysyl-tRNA synthetase [*A. thaliana*]	Contig19986_at	-1.07	-2.41	NP_187777.1	7e-85
Receptor-like kinase ARK1AS [*T. aestivum*]	Contig4997_s_at	1.1	-2.41	AAD43962.1	4e-90
SNF1 like protein kinase [*A. thaliana*]	Contig14822_at	-1.28	-2.4	NP_193194.1	2e-16
SnRK1-type protein kinase [*H. vulgare*]	Contig6240_at	1.22	-2.4	CAA07813.1	e-100
Similar to putative DNA-binding protein RAV2[*O. sativa* (japonica)]	HS16C04u_at	1.1	-2.34	BAA85426.1	1e-10
Myb family transcription factor [*A. thaliana*]	Contig8132_at	-1.05	-2.29	NP_190199.1	5e-46
Histone H2B.2 pir [*T. aestivum*]	Contig1161_at	-1.54	-2.28	P05621	8e-64
Putative DNA polymerase alpha subunit [*A. thaliana*]	Contig15791_at	1.25	-2.28	NP_176930.1	3e-09
Probable aspartate kinase / homoserine dehydrogenase [*O. sativa*]	Contig8678_at	-1.12	-2.28	T03589	e-107
ADP-ribosylation factor [*A. thaliana*]	HW02M24u_at	1.11	-2.28	NP_188935.1	2e-12
Protein kinase-like protein [*O. sativa*]	Contig6395_at	-1.11	-2.26	AAG13605.1	6e-30

DNA topoisomerase II [*P. sativum*]	Contig10468_at	-1.02	-2.24	O24308	4e-24
Histone H2B153 [*T. aestivum*]	Contig1164_at	-1.62	-2.24	S56687	3e-53
Putative uncharacterized protein At5g38600[*A. thaliana*]	Contig18250_at	1.05	-2.24	NP_568558.1	4e-48
P0019E03.6 [*O. sativa* (japonica)]	EBro08_SQ007_N06_at	1.03	-2.23	BAC01248.1	5e-21
Putative protein; protein id: At5g56470.1 [*A. thaliana*]	HVSMEf0019G08r2_at	-1.09	-2.23	NP_200458.1	2e-12
Putative RNA-binding like protein [*O. sativa* (japonica)]	HV04H12r_at	-1.1	-2.21	BAB63863.1	3e-05
Histone H2A [*C. arietinum*]	Contig119_at	-1.66	-2.2	O65759	5e-41
Putative protein kinase [*O. sativa* (japonica)]	HU12B19u_at	-1.16	-2.2	AAK27806.1	1e-43
Origin recognition complex subunit 4 [*Z. mays*]	HVSMEc0013C11r2_at	-1.14	-2.2	AAL10455.1	9e-41
(AC087599) putative gag-pol polyprotein [O. sativa]	HVSMEb0012L22f_at	-1.02	-2.19	AAL79695.1	2e-48
Histone H3 [*T. aestivum*]	HA28D21r_at	-1.06	-2.18	HSPM3	1e-45
Contains similarity to transcription regulator [*A. thaliana*]	HV_CEa0014K16r2_at	1.22	-2.16	BAB09481.1	2e-10
Putative reverse transcriptase [*O. sativa* (japonica)]	Contig14933_s_at	1.02	-2.14	AAM22009.1	2e-10
Histone H4 (TH091) [*T. aestivum*]	Contig30_s_at	-1.53	-2.14	HSWT41	8e-40
LHY protein [Phaseolus vulgaris]	HV_CEa0006F14r2_x_at	-1.23	-2.14	CAD12767.2	8e-08
Putative promoter-binding factor-like protein [*O. sativa* (japonica)]	Contig10705_at	1.45	-2.13	BAB85251.1	2e-35
Putative SPATULA [O. sativa] [*O. sativa* (japonica)]	Contig15975_at	-1.31	-2.12	AAK98706.1	4e-32
Histone H2B.2 [*T. aestivum*]	Contig1154_s_at	-1.25	-2.11	P05621	1e-57
Nucleolin like protein CiRGG1 [*Ciona intestinalis*]	Contig14256_at	-1.2	-2.11	BAA86200.1	3e-04
Similar to *H. sapiens* mRNA for KIAA0039. [*O. sativa* (japonica)]	Contig19021_at	-1.01	-2.1	BAA92952.1	8e-28
Similar to tRNA-splicing endonuclease positive effector[*A. thaliana*]	Contig20164_at	-1.09	-2.09	AAG09081.1	1e-04
Putative protein; protein id: At5g50200.1 [*A. thaliana*]	Contig4953_at	-1.07	-2.09	NP_199831.1	1e-29
Putative protein; protein id: At3g47610.1 [*A. thaliana*]	Contig15980_at	-1.11	-2.08	NP_190345.1	2e-69
Putative retrotransposon polyprotein [*O. sativa* (japonica)]	Contig15103_at	-1.01	-2.07	BAB89058.1	7e-42
Nucleoid DNA-binding - like protein[*A. thaliana*]	Contig9236_at	-1.14	-2.07	NP_191008.1	6e-17
Putative maize transposon MuDR mudrA-like protein [*O. sativa*]	Contig8251_at	-1.07	-2.05	AAK00423.2	5e-99
Transposase-like protein [*O. sativa* (japonica)]	Contig20121_at	1.09	-2.04	BAC20878.1	3e-15
Expressed protein; protein id: At2g44310.1[*A. thaliana*]	Contig7016_s_at	-1.03	-2.04	NP_566015.1	1e-29
Actin 2 [Anemia phyllitidis]	HT12B11u_at	-1.06	-2.04	AAC64127.1	9e-33
T31J12.4 [*A. thaliana*]	Contig10224_at	-1.04	-2.02	AAD18097.1	6e-26
Histone H2B-8 [*T. aestivum*]	Contig1169_x_at	-1.41	-2.02	S56685	9e-50
Similar to *H. sapiens* splicing factor [*O. sativa* (japonica)]	Contig3729_at	-1.06	-2.02	BAA78744.1	6e-44
Transcription initiation factor TFIID subunit 1-A [*A. thaliana*]	Contig16462_at	-1.15	-2.01	AAF25977.1	1e-36
RNaseP-associated protein-like [*O. sativa* (japonica)]	Contig12461_at	1.02	-2	BAC16391.1	1e-62
DEAD/DEAH box helicase carpel factory-related [*A. thaliana*]	Contig15447_at	-1.01	-2	NP_566199.2	2e-56
Similar to Ubiquitin-conjugating enzyme E2-17 KD [*A. thaliana*]	Contig3793_at	-1.22	-2	AAC83026.1	6e-12
At5g11350/F2I11_240 [*A. thaliana*]	Contig10824_at	-1.08	-2.45	AAL47464.1	2e-34
**Carbohydrate metabolism**					
NADP-specific isocitrate dehydrogenase [*O. sativa*]	rbasd27j06_at	-1.22	-5.59	AAD37809.1	7e-52
Putative epoxide hydrolase [*O. sativa* (japonica)]	Contig15599_at	1.06	-3.53	AAL58264.1	1e-40
Saccharopin dehydrogenase-like protein [*H. vulgare*]	Contig7064_s_at	1.28	-3.5	CAD48130.1	8e-59
Glucan endo-1,3-beta-glucosidase GIV [*H. vulgare*]	Contig1641_at	1.19	-3.33	Q02437	3e-84
High pI alpha-glucosidase [*H. vulgare*]	Contig7938_at	-1.05	-3.25	AAF76254.1	e-114
Beta-glucosidase homolog F8K4.3 [*A. thaliana*]	Contig2864_at	1.54	-3.11	T02128	5e-42
Putative mannan endo-1,4-beta-mannosidase [*O. sativa* (japonica)]	HY04F23u_s_at	-1.01	-2.48	BAB91747.1	0.011
Ribulose bisphosphate carboxylase [*T. aestivum*]	rbaal24m19_at	-1.84	-2.39	CAA25058.1	6e-04
Cinnamyl alcohol dehydrogenase [*L. perenne*]	Contig4260_at	-1.38	-2.34	AAL99535.1	4e-89
Aldehyde oxidase [*Z. mays*]	Contig4920_s_at	-1.22	-2.34	T01698	e-119
Basic blue protein [Medicago sativa subsp. x varia]	Contig16272_at	-1.17	-2.33	CAB65280.1	7e-22
UDP-glucose 4-epimerase [*O. sativa* (japonica)]	HS05O02u_at	1.02	-2.32	BAC02925.1	1e-17
Putative hydrolase [*A. thaliana*]	Contig20832_s_at	1.3	-2.31	AAM65281.1	1e-26
Alcohol dehydrogenase [*H. vulgare*]	Contig278_at	1.23	-2.24	AAK49116.1	e-119
Putative PAP-specific phosphatase [*O. sativa*]	Contig20457_at	-1.06	-2.2	AAK98703.1	2e-24
12-oxophytodienoic acid reductase [*O. sativa*]	Contig19759_at	1.05	-2.17	BAC20139.1	7e-35
Glycosyl hydrolase family 85 [*A. thaliana*]	Contig25899_at	-1.06	-2.14	NP_196165.1	2e-15
Glucan endo-1,3-beta-D-glucosidase[*H. vulgare*]	HVSMEg0017E11r2_x_at	-1.51	-2.13	JC1438	1e-38
Alcohol dehydrogenase-like protein [*O. sativa* (japonica)]	Contig20626_at	-1.14	-2.12	BAC10092.1	4e-13
Aconitate hydratase, cytoplasmic[*Cucurbita maxima*]	Contig2866_s_at	-1.16	-2.08	P49608	e-117
NADPH-dependent HC-toxin reductase [*H. vulgare*]	Contig11146_at	-1.98	-2.04	T06197	5e-98
Endo-1,4-beta-glucanase [*L. esculentum*]	Contig13215_at	-1.27	-2.04	T07612	4e-85
Putative NAD-malate dehydrogenase [*O. sativa* (japonica)]	HVSMEg0003A16r2_s_at	-1.35	-2.03	BAB68116.1	1e-19
Beta-glucosidase isozyme 2 precursor [*O. sativa* (japonica)]	Contig11769_at	-1.06	-2.01	AAL14713.1	3e-80
Glycosyl hydrolase family 5/cellulase [*A. thaliana*]	Contig19876_at	1.04	-2.01	NP_194561.1	2e-12

**Cell growth, division**					
Coiled-coil protein; putative spindle pole body by similarity to yeast nuf2 [*S. pombe*]	HT08F01u_at	-1.08	-3	NP_594532.1	2e-07
putative cell differentiation protein [*O. sativa*]	HB12G06r_at	1.11	-2.52	AAK72897.1	7e-52
Genetic modifier [*Z. mays*]	Contig17881_at	1.44	-2.45	AAG17894.1	1e-69
Centromere/kinetochore protein zw10 homolog [*A. thaliana*]	Contig19980_at	-1.2	-2.34	O48626	2e-29
Auxin response factor 2 [*O. sativa* (japonica)]	HV_CEb0003E19r2_at	1.1	-2.25	BAB92903.1	8e-19
(U19134) SABRE [*A. thaliana*]	Contig22689_at	-1.17	-2.06	AAC49734.1	1e-54
Auxin response factor 7b [*O. sativa*]	Contig14766_s_at	-1.15	-2	BAB85917.1	4e-66
**Fat metabolism**					
Lipoxygenase 2 [*H. vulgare*]	HVSMEf0009L21r2_s_at	-1.36	-2.46	T05945	6e-44
Outer membrane lipoprotein-like [*A. thaliana*]	Contig10258_at	-1.06	-2.43	AAM62904.1	3e-05
Glutamic acid-rich protein [*Plasmodium falciparum*]	Contig24609_at	1.13	-2.39	AAG14291.1	5e-05
GDSL-motif lipase/hydrolase-like protein [*O. sativa* (japonica)]	Contig19958_at	1.19	-2.19	BAB21153.1	3e-25
Sulfolipid synthase [*A. thaliana*]	Contig16651_at	1.13	-2.04	AAM18913.1	e-109
**Nitrogen metabolism**					
AT3g03380/T21P5_20 [*A. thaliana*]	Contig7623_at	1.07	-2.96	AAL84951.1	1e-98
Putative sarcosine oxidase [*O. sativa* (japonica)]	Contig17178_at	-1.09	-2.77	BAB90345.1	5e-45
Proline-rich protein 2 [*O. sativa* (japonica)]	Contig970_at	-1.18	-2.64	AAK63895.1	4e-13
Putative proline-rich protein [*O. sativa*]	Contig4725_at	-1.63	-2.63	AAK91881.1	4e-44
Putative amidase [*O. sativa* (japonica)]	Contig21273_at	1.11	-2.48	AAL75736.1	4e-47
Saccharopine dehydrogenase [*Candida albicans*]	EBro08_SQ002_M03_at	1.03	-2.28	P43065	1e-24
L-allo-threonine aldolase [*A. thaliana*]	HVSMEg0009L14r2_at	-1.01	-2.28	NP_566228.1	9e-28
Legumain-like protease [*Z. mays*]	HVSMEk0005A23r2_at	-1.06	-2.1	CAB64545.1	4e-36
Fruit bromelain [*Ananas comosus*]	Contig23868_at	-1.15	-2.07	T10518	4e-12
Chloroplast protein-translocon-like protein [*O. sativa*]	HVSMEb0011P07r2_at	1.09	-2.01	AAK50116.1	2e-20
Spermidine synthase 1 [*O. sativa* (japonica)]	Contig4624_at	-1.05	-7.12	Q9SMB1	e-125
Putative aminotransferase [*A. thaliana*]	rbags11h24_s_at	-1.06	-5.3	NP_187498.1	4e-10
Hordein B H. vulgare var. distichum [*H. vulgare*]	Contig811_x_at	1.07	-4.47	1103203A	4e-14
Putative subtilisin-like protease [*O. sativa* (japonica)]	Contig18723_at	1.18	-4.27	BAB89803.1	5e-27
C13 endopeptidase NP1 precursor [*H. vulgare*]	Contig3371_at	1.7	-3.85	AAD04882.1	e-101
Peptidyl-prolyl cis-trans isomerase [*S. pombe*]	Contig26213_at	-1.12	-3.82	NP_595257.1	4e-26
B3-hordein (clone pB7) [*H. vulgare*]	Contig549_x_at	-1.19	-3.73	S07975	1e-48
Keratin associated protein 5-4 [*M. musculus*]	EBro03_SQ008_I11_at	-1.11	-3.26	NP_056624.1	0.017
Keratin associated protein 1.1 [*H. sapiens*]	Contig13708_s_at	-1.08	-2.55	NP_112229.1	0.06
B3-hordein (clone pB7) [*H. vulgare*]	Contig301_x_at	-1.06	-2.54	S07975	9e-45
B3-hordein (clone pB7) [*H. vulgare*]	Contig523_x_at	-1.27	-2.41	S07975	2e-50
Alpha-amylase/trypsin inhibitor CMb [*H. vulgare*]	Contig18_s_at	1.02	-2.31	P32936	9e-87
Aromatic-L-amino-acid decarboxylase [*Catharanthus roseus*]	Contig23667_at	1.18	-2.31	P17770	3e-15
Gamma-hordein 3[*H. vulgare*]	Contig209_s_at	-1.08	-2.11	T05718	2e-70
B3-hordein (clone pB7) [*H. vulgare*]	Contig765_x_at	1.03	-2	S07975	9e-44
Putative serine/threonine phosphatase type 2c [*Sporobolus stapfianus*]	Contig10323_at	-1.6	-2.28	CAB61839.1	5e-56
**Photosynthesis**					
Photosystem I P700 chlorophyll A [*Z. mays*]	HVSMEc0016D02f_at	-1.36	-7.33	P04966	7e-52
Photosystem I P700 apoprotein A1 [*T. aestivum*]	HVSMEc0016J13f_at	-1.25	-7.16	NP_114259.1	2e-17
Photosystem IP700 apoprotein A1 [*T. aestivum*]	HV_CEa0013J19f_at	-1.27	-3.48	NP_114259.1	8e-75
Ferredoxin-NADP(H) oxidoreductase [*T. aestivum*]	Contig2275_at	1.14	-4.01	CAD30024.2	9e-36
**Protein synthesis**					
60S acidic ribosomal protein-like protein [*Wuchereria bancrofti*]	EBro08_SQ003_O09_at	1.2	-3.04	AAG31479.1	1e-10
Ribosomal protein L28-like [*O. sativa* (japonica)]	HVSMEg0001I10f_at	1.38	-2.84	BAB86520.1	5e-04
60S ribosomal protein L17 [*D. melanogaster*]	Contig21048_at	-1.04	-2.81	NP_572346.1	2e-29
Ribosomal protein S14 [*T. aestivum*]	HVSMEc0009D09f_at	1.01	-2.81	NP_114257.1	2e-38
Mitochondrial ribosomal protein S32 [*D. melanogaster*]	Contig26222_at	1.25	-2.43	NP_523673.1	1e-12
Translation elongation factor eEF-1 beta chain [*Bombyx mori*]	Contig23900_at	1.1	-2.33	P29522	6e-50
Putative basic protein [*O. sativa*]	Contig195_at	-1.1	-2.3	AAK38502.1	3e-25
Ribosomal protein L28-like [*O. sativa* (japonica)]	EBro08_SQ012_A23_at	-1.11	-2.22	BAB86520.1	2e-15
HMG-CoA synthase [*Brassica juncea*]	Contig9135_at	-1.24	-2.2	AAF69804.1	e-107
Phytochelatin synthetase-like protein [*Z. mays*]	rbah36c02_s_at	1.15	-2.14	AAF24189.1	4e-82
Sucrose-phosphate synthase [*H. vulgare*]	HVSMEc0008G05r2_at	1.09	-2.12	AAF75266.1	5e-31
Long-chain acyl-CoA synthetase [*A. thaliana*]	Contig6771_at	1.11	-2.1	BAB40450.1	e-104
Putative snRNP protein [*S. bicolor*]	HV03I05u_at	1.1	-2.09	AAM94328.1	3e-12

50S ribosomal protein L33 [*Mesorhizobium loti*]	HC01D11_T3_at	1.35	-2.05	NP_102563.1	5e-10
40S ribosomal protein S13, cytosolic [*Z. mays*]	HVSMEa0011C02r2_x_at	-1.08	-2.01	Q05761	5e-15
(AC087181) putative snRNP protein [*O. sativa*]	HT08E21u_at	1.38	-2	AAK38497.1	0.002
**Signal transduction**					
Ethylene-responsive small GTP-binding protein [*L. esculentum*]	Contig3337_at	1.06	-3.04	AAD46405.1	4e-94
19A protein [*H.sapiens*]	EBro02_SQ006_D14_s_at	-1.39	-2.91	CAB81950.2	0.6
Contains similarity to phosphoesterase [*A. thaliana*]	Contig17557_at	-1.05	-2.9	NP_200628.1	8e-41
Putative membrane protein family member [*Caenorhabditis elegans*]	HVSMEc0004C11f_x_at	-1.3	-2.82	NP_507189.1	0.3
Putative kinase [*O. sativa* (japonica)]	HA11C22r_at	-1.04	-2.74	AAL58279.1	1e-11
Putative protein kinase [*S. bicolor*]	Contig10013_at	-1.97	-2.64	AAM47583.1	e-114
S-locus receptor-like kinase RLK11 [*O. sativa*]	Contig25306_at	1.07	-2.62	AAM90696.1	1e-76
Putative serine threonine kinase [*O. sativa* (japonica)]	Contig26011_at	1.08	-2.58	BAC05563.1	1e-31
Similar to Probable microsomal signal peptidase [*A. thaliana*]	Contig5094_at	-1.28	-2.5	NP_680778.1	7e-22
Putative myosin heavy chain [*O. sativa* (japonica)]	Contig20373_at	1.06	-2.4	AAL83688.1	2e-84
Histidine-containing phosphotransfer protein-like [*A. thaliana*]	Contig19622_at	-1.21	-2.38	BAB01275.1	4e-31
RAB5A protein [*O. sativa*]	Contig6921_at	-1.37	-2.36	CAC19792.1	3e-83
Putative 32.7 kDa jasmonate-induced protein [*H. vulgare*]	Contig7887_at	-1.22	-2.22	T04375	e-168
Putative receptor protein kinase-like protein [*O. sativa* (japonica)]	HVSMEm0005L12r2_at	1.01	-2.17	BAB63567.1	4e-20
Putative GTP-binding protein, ERG [*A. thaliana*]	Contig15500_at	1	-2.16	NP_174383.1	1e-79
Kinesin-like protein [*A. thaliana*]	Contig24103_at	1.09	-2.13	AAM97018.1	6e-18
Protein kinase-like [*A. thaliana*]	Contig7153_at	-1.03	-2.12	NP_198447.1	5e-34
Putative protein kinase [*O. sativa* (japonica)]	Contig10039_at	-1.04	-2.07	BAB92217.1	e-101
Serine/threonine kinase-like protein [*O. sativa* (japonica)]	rbaal11f18_at	-1.46	-2.07	BAC20671.1	7e-56
Myosin heavy chain-like; protein id: At5g55860.1 [*A. thaliana*]	Contig12526_at	-1.03	-2.05	NP_200397.1	4e-43
Small Ran-related GTP-binding protein [*T. aestivum*]	Contig1812_at	-1.28	-2.04	AAL30396.1	e-120
GTP-binding protein RAB1[*Petunia x hybrida*]	Contig2536_at	-1.07	-2.04	S72515	8e-22
Expressed protein; protein id: At2g46090.1 [*A. thaliana*]	Contig15531_at	-1.03	-2.01	NP_566064.1	1e-30
**Unknown classified**					
Unknown protein; protein id: At2g42760.1 [*A. thaliana*]	Contig15187_at	1.07	-7.91	NP_181804.1	8e-07
Hypothetical protein [*P. yoelii yoelii*]	Contig464_at	1.09	-5.46	EAA16547.1	9e-09
Unknown protein [*O. sativa*]	HM03H03u_at	-1.42	-5.23	AAK91879.1	1e-25
Hypothetical protein [*Cytophaga hutchinsonii*]	EBro08_SQ009_E20_at	-1.08	-5.12	ZP_00119259 .	7e-38
ORF133; ORF within trnI intron [*O. sativa* (japonica)]	HVSMEa0020P01f_at	-1	-4.86	NP_039436.1	8e-19
Putative protein; protein id: At5g18460.1 [*A. thaliana*]	HVSMEl0002A04r2_at	-1.03	-4.38	NP_197347.1	8e-23
Hypothetical protein [*O. sativa* (japonica)]	HS03K03u_at	-1.03	-4.2	BAB92927.1	1e-34
Unknown protein [*O. sativa* (japonica)]	Contig24782_at	1.15	-4.08	BAB90754.1	e-107
Hypothetical protein [Oenothera elata subsp. hookeri]	ChlorContig11_s_at	-1.38	-4.02	NP_084748.1	3e-13
Hypothetical protein [*O. sativa*]	Contig4816_x_at	-1.19	-3.99	AAK98749.1	2e-25
B12D protein [*Ipomoea batatas*]	HV_CEa0011J09r2_at	-1.16	-3.86	AAD22104.1	6e-16
Unknown protein [*A. thaliana*]	Contig10426_x_at	1.16	-3.84	NP_188597.1	2e-93
Putative protein; protein id: At4g28000.1 [*A. thaliana*]	HVSMEa0014K13r2_at	1.08	-3.56	NP_194529.1	3e-19
Putative protein [*A. thaliana*]	Contig5859_at	1.02	-3.46	NP_566883.1	5e-19
Hypothetical protein [*P. yoelii yoelii*]	Contig464_x_at	-1.17	-3.34	EAA16547.1	9e-09
Putative uncharacterized protein [*O. sativa* (japonica)]	HVSMEb0002E18r2_at	1.32	-3.22	BAC45101.1	6e-06
Putative protein; protein id: At4g11090.1 [*A. thaliana*]	Contig13805_at	-1.1	-3.16	NP_192847.1	8e-31
Conserved hypothetical protein [*O. sativa*]	HS16I24u_at	1.08	-3.16	AAK51570.1	2e-17
putative protein; protein id: At5g48520.1 [*A. thaliana*]	HVSMEg0005J17r2_at	-1.18	-3.12	NP_199663.1	0.012
Expressed protein; protein id: At3g15470.1[*A. thaliana*]	Contig15774_at	-1.17	-3.11	NP_566515.1	2e-23
Hypothetical protein [*O. sativa* (japonica)]	Contig7736_at	-1.2	-3.09	BAB62552.1	2e-51
Putative protein [*A. thaliana*]	Contig15193_at	-1.11	-3.08	NP_568458.1	6e-18
Hypothetical protein [*O. sativa*]	Contig3364_at	-1.29	-2.95	AAM08574.1	6e-32
F21F23.21 protein [*A. thaliana*]	Contig22133_at	1.13	-2.89	AAF81304.1	5e-32
Hypothetical protein [*O. sativa* (japonica)]	Contig11242_at	1.06	-2.85	BAC00744.1	7e-62
oj991113_30.13 [*O. sativa* (japonica)]	Contig11974_at	-1.11	-2.85	CAD41331.1	e-118
F1B16.1 protein [*A. thaliana*]	HU05L05u_s_at	1.07	-2.85	NP_177679.1	2e-06
Hypothetical protein [*P. yoelii yoelii*]	HVSMEc0009K16f_s_at	-1.25	-2.85	EAA16547.1	0.1
Unnamed protein product [*M. musculus*]	MitoContig11_at	-1.23	-2.85	BAC26016.1	0.6
Zwh19.1 [*O. sativa* (indica)]	Contig6484_at	-1.36	-2.84	CAB55403.1	2e-62
Unknown protein [*O. sativa* (japonica)]	Contig13555_at	1.06	-2.82	BAB92456.1	1e-59
Hypothetical protein [*O. sativa* (japonica)]	Contig17974_at	1.07	-2.81	BAB68066.1	8e-43
Hypothetical protein [*A. thaliana*]	HVSMEa0019H10r2_at	1.04	-2.81	NP_178398.1	4e-67

Putative protein; protein id: At4g33550.1 [*A. thaliana*]	Contig1614_s_at	-1.38	-2.79	NP_195081.1	7e-07
AgCP4709 [Anopheles gambiae str. PEST]	HM11L09r_at	-1.06	-2.76	EAA15143.1	8e-05
Putative caltractin [*O. sativa* (japonica)]	Contig14172_at	-1.12	-2.72	BAC10116.1	2e-05
Hhypothetical protein [*Oenothera elata*]	HVSMEc0005H22f_x_at	-1.24	-2.69	NP_084748.1	4e-13
Hhypothetical protein [O.enothera elata]	ChlorContig11_x_at	-1.3	-2.68	NP_084748.1	3e-13
Hhypothetical protein [*O. sativa* (japonica)]	Contig847_at	1.19	-2.66	AAN05005.1	5e-07
Pprobable integral membrane protein[*P. falciparum*]	HV09J08u_s_at	-1.07	-2.65	E71602	0.025
Unknown protein [*A. thaliana*]	Contig12229_at	-1.2	-2.62	AAL87254.1	2e-09
Hypothetical protein [*O. sativa* (japonica)]	HS09P03u_s_at	-1.25	-2.62	BAA99518.1	3e-22
Zhb0001.1 [*O. sativa* (indica)]	Contig18368_at	-1.05	-2.59	CAB55409.1	1e-53
OSJNBa0059D20.15 [*O. sativa* (japonica)]	Contig15751_at	-1.19	-2.58	CAD39760.1	3e-85
Hypothetical protein [*A. thaliana*]	Contig8975_at	-1.11	-2.57	NP_179383.1	e-102
P0674H09.13 [*O. sativa* (japonica)]	Contig21050_at	1.14	-2.54	BAC06891.1	1e-32
Hypothetical protein [*O. sativa* (japonica)]	Contig22709_at	-1.16	-2.54	BAB61209.1	2e-05
Hypothetical protein [*O. sativa* (japonica)]	Contig25764_at	1.11	-2.54	BAC06228.1	9e-81
Putativeprotein;proteinid:At5g10080.1[*A. thaliana*]	Contig15913_at	-1.26	-2.52	NP_196570.1	7e-21
Expressed protein [*A. thaliana*]	Contig17314_at	-1.66	-2.52	NP_567276.1	7e-91
Hypothetical protein [*O. elata*]	HVSMEc0011H17f_x_at	-1.21	-2.52	NP_084748.1	1e-13
Unknown protein [*A. thaliana*]	Contig25688_at	-1.04	-2.51	BAB02559.1	2e-44
Putative protein [*A. thaliana*]	Contig14701_at	-1.06	-2.5	NP_196075.1	7e-25
F21M12.18 gene product [*A. thaliana*]	Contig16782_at	-1.02	-2.5	AAB60734.1	3e-08
Expressed protein [*A. thaliana*]	Contig9532_at	-1.17	-2.43	NP_565146.1	5e-47
F10B6.18 [*A. thaliana*]	Contig12017_at	1.09	-2.41	AAF79223.1	7e-39
Putative protein; protein id: At4g23790.1 [*A. thaliana*]	Contig16114_at	1.02	-2.39	NP_194110.1	3e-24
Hypothetical protein [*O. sativa* (japonica)]	Contig14678_at	1.03	-2.38	AAN62783.1	5e-21
AT5g18530/T28N17_10 [*A. thaliana*]	Contig22743_at	1.01	-2.38	AAL36064.1	5e-36
Unnamed protein product [*H. sapiens*]	HD10K20r_at	-1.2	-2.38	BAC04906.1	6e-05
Putative protein [*A. thaliana*]	HK03H05r_s_at	-1.21	-2.38	NP_195798.1	4e-22
Hypothetical protein [*A. thaliana*]	Contig16470_at	-1.15	-2.37	NP_176909.1	4e-50
Corresponds to a region of the predicted gene[*A. thaliana*]	Contig13874_at	1.11	-2.36	BAA81762.1	4e-33
Unknown protein [*A. thaliana*]	Contig15058_at	1.26	-2.36	NP_199712.1	5e-30
Expressed protein [*A. thaliana*]	Contig16664_at	-1.15	-2.36	NP_565058.1	2e-70
Hypothetical protein [*O. sativa* (japonica)]	Contig23909_at	1.1	-2.36	BAC03362.1	2e-18
Hypothetical protein [*O. sativa* (japonica)]	HA12B04u_at	-1.13	-2.33	BAB86070.1	2e-45
B1103C09.5 [*O. sativa* (japonica)]	Contig11329_at	1.02	-2.32	BAB91815.1	2e-30
AgCP4592 [A. gambiae str. PEST]	Contig26188_at	-1.21	-2.32	EAA14931.1	2e-36
Putative protein [*A. thaliana*]	Contig5857_s_at	1.1	-2.32	NP_566883.1	2e-64
Putative protein [*A. thaliana*]	Contig6165_at	1.28	-2.32	NP_194519.1	5e-18
hypothetical protein [*A. thaliana*]	Contig7467_at	-1.11	-2.32	NP_187305.1	9e-57
Hypothetical protein [*A. thaliana*]	Contig26435_at	-1.23	-2.3	NP_180174.1	1e-46
Putative protein [*A. thaliana*]	HX04E02u_at	-1.09	-2.3	NP_567930.1	2e-19
Putative protein; protein id: At5g12920.1 [*A. thaliana*]	Contig6877_s_at	-1.03	-2.29	NP_196796.1	9e-59
Putative protein; protein id: At4g21670.1 [*A. thaliana*]	Contig14797_at	1.03	-2.27	NP_193898.1	2e-14
Hypothetical protein [*A. thaliana*]	Contig14724_at	1.25	-2.26	NP_194848.1	5e-41
Hypothetical protein [*O. sativa* (japonica)]	Contig23511_at	-1.12	-2.26	BAC16035.1	2e-50
Unnamed protein product [*M. musculus*]	EBma08_SQ002_M18_at	-1.51	-2.26	BAC27870.1	0.5
Hypothetical protein [*O. sativa* (japonica)]	Contig17164_at	-1.37	-2.25	BAB84388.1	1e-99
Unknown protein [*A. thaliana*]	Contig17265_at	-1.7	-2.25	NP_173694.1	1e-88
Putative protein; protein id: At4g29850.1 [*A. thaliana*]	Contig24918_at	-1.16	-2.25	NP_194714.1	2e-21
Wax synthase-like protein [*O. sativa* (japonica)]	Contig22367_at	1.23	-2.24	BAB89209.1	7e-13
Unknown protein [*O. sativa* (japonica)]	Contig3265_at	-1.09	-2.24	BAB86162.1	9e-08
Hypothetical protein [*O. sativa* (japonica)]	HV05C09u_at	-1.09	-2.24	BAB63529.1	2e-18
Unknown protein [*O. sativa* (japonica)]	Contig10905_at	-1.38	-2.23	AAK71559.1	4e-25
OSJNBa0006B20.5 [*O. sativa* (japonica)]	HX03D06u_at	-1.06	-2.23	CAD40814.1	3e-09
Unknow protein F2G19.25 [*A. thaliana*]	Contig10563_at	1.08	-2.2	BAB78681.1	2e-50
Unknown protein [*A. thaliana*]	Contig15414_at	1.03	-2.2	NP_180229.2	2e-51
Hypothetical protein [*O. elata*]	HVSMEc0005O17f_x_at	-1.19	-2.19	NP_084748.1	2e-13
Putative protein [*A. thaliana*]	Contig19225_at	1.3	-2.18	NP_199082.1	5e-25
Unnamed protein product [*O. sativa* (japonica)]	Contig14223_at	-1.04	-2.16	BAB03418.1	6e-59
Putative protein [*A. thaliana*]	Contig7503_at	1.03	-2.16	AAM97033.1	5e-57
Unknown protein [*O. sativa* (japonica)]	Contig9509_at	1.06	-2.16	BAB90287.1	8e-80
Hypothetical protein [*O. sativa*]	Contig10352_s_at	1.05	-2.15	AAK98733.1	e-107
Corresponds to a region of the predicted [*A. thaliana*]	HM09B02r_x_at	1.06	-2.15	BAA81762.1	2e-26

OSJNBa0067K08.21 [*O. sativa* (japonica)]	HZ01B01u_at	-1.04	-2.15	CAD41257.1	7e-26
Hypothetical protein [*O. sativa* (japonica)]	Contig13806_at	-1.56	-2.14	BAB63562.1	6e-91
F18O14.5 [*A. thaliana*]	Contig17801_at	-1.16	-2.13	AAF79454.1	3e-16
Putative protein [*A. thaliana*]	Contig18434_at	-1.17	-2.13	NP_191337.1	1e-56
Unnamed protein product [*O. sativa* (japonica)]	Contig2446_x_at	-1.21	-2.13	BAA87836.1	1e-09
Hypothetical protein [*O. sativa* (japonica)]	Contig9465_at	-1.12	-2.13	BAC20075.1	1e-48
Putative protein [*A. thaliana*]	Contig10463_at	-1.03	-2.12	NP_566873.1	6e-19
Putative protein [*A. thaliana*]	Contig10592_at	-1.04	-2.12	NP_197990.1	9e-73
Hypothetical protein [*O. sativa* (japonica)]	Contig13237_at	-1.08	-2.12	BAB39914.1	1e-92
Unnamed protein product [*O. sativa* (japonica)]	Contig2446_at	-1.04	-2.12	BAA87836.1	1e-09
Putative protein; protein id: At5g12920.1 [*A. thaliana*]	Contig6876_at	-1.19	-2.12	NP_196796.1	1e-21
Hypothetical protein [*O. sativa* (japonica)]	Contig13064_at	1.04	-2.11	BAB90072.1	3e-76
Unknown protein [*A. thaliana*]	Contig26285_at	-1.17	-2.11	NP_173104.1	1e-32
At1g80210/F18B13_28 [*A. thaliana*]	Contig6298_at	-1.03	-2.11	AAM10390.1	9e-63
Putative protein; protein id: At3g60850.1 [*A. thaliana*]	HX09G24r_at	1.12	-2.11	NP_191644.1	0.002
Hypothetical protein [*A. thaliana*]	Contig16504_at	-1.51	-2.1	AAD25929.1	5e-15
Unknown [Aegilops tauschii]	Contig8003_at	-1.02	-2.1	AAM69851.1	3e-22
Unknown protein [*A. thaliana*]	HVSMEb0001J13r2_at	-1.24	-2.1	AAM13860.1	4e-11
Hypothetical protein [*O. sativa* (japonica)]	Contig13039_x_at	-1.02	-2.09	BAB40040.1	4e-21
Hypothetical protein [*O. sativa* (japonica)]	Contig8656_at	-1.18	-2.09	BAB92174.1	9e-98
Hypothetical protein [*O. sativa* (japonica)]	Contig20118_at	-1.09	-2.08	BAB21185.1	e-101
T10O24.24 [*A. thaliana*]	Contig14914_at	-1.05	-2.06	AAD39584.1	4e-32
OSJNBa0059D20.4 [*O. sativa* (japonica)]	Contig20914_at	-1.17	-2.06	CAD39749.1	1e-14
P0028G04.3 [*O. sativa* (japonica)]	Contig9514_at	1.1	-2.06	BAB93427.1	2e-60
Putative protein [*A. thaliana*]	Contig9113_at	-1.33	-2.04	NP_568256.1	2e-95
Unknown protein [*A. thaliana*]	Contig13723_at	-1.35	-2.03	NP_179158.1	3e-23
Unknown [A. thaliana]	Contig10651_at	1.03	-2.02	AAM61120.1	6e-18
Hypothetical protein [*O. sativa* (japonica)]	Contig9465_s_at	-1.26	-2.02	BAC20075.1	1e-48
Unknown protein [*A. thaliana*]	Contig15350_at	1.07	-2.01	NP_201396.2	2e-42
Early nodulin 75 precursor-like protein [*O. sativa* (japonica)]	Contig14279_at	-1.21	-2	BAC21402.1	7e-06
Putative protein [*A. thaliana*]	Contig20973_at	1.04	-2	NP_193695.1	1e-34
Hypothetical protein [*O. sativa* (japonica)]	HVSMEg0005B12r2_at	-1.26	-2	BAB40147.1	5e-31
AgCP8133 [A. gambiae str. PEST]	EBro02_SQ007_I24_at	-1.3	-3.95	EAA13878.1	1e-15
Serine protease-like protein [*O. sativa* (japonica)]	Contig18045_at	-1.03	-2.47	BAC10341.1	9e-57
(AL662945) oj000126_13.14 [*O. sativa* (japonica)]	rbags22c23_at	-1.09	-2.02	CAD40592.1	5e-39
**None**					
none	HT09I05u_x_at	-1.97	-19.84	none	none
none	HVSMEn0013N19f_s_at	1.23	-15.46	none	none
none	HV12B15u_at	1.11	-12.08	none	none
none	Contig20565_at	-1.65	-10.16	none	none
none	S0000800242F04F1_at	-1.08	-8.74	none	none
none	Contig18391_x_at	1.05	-8.24	none	none
none	HVSMEg0005I15r2_at	-1.81	-8.21	none	none
none	HV_CEa0012H15r2_at	-1.42	-7.41	none	none
none	S0001000018H09F1_at	-1.05	-7.21	none	none
none	EBes01_SQ003_N19_at	1.21	-6.69	none	none
none	HVSMEn0025E24r2_at	-1.11	-5.81	none	none
none	EBed02_SQ002_E13_x_at	1.38	-5.62	none	none
none	HS06C04u_at	1.04	-5.6	none	none
none	HVSMEn0018J02f_x_at	1.07	-5.29	none	none
none	HV_CEa0002I08r2_at	-1.15	-5.18	none	none
none	HF14A04r_at	1.07	-5.09	none	none
none	Contig9493_at	1.19	-4.73	none	none
none	Contig7914_at	-1.35	-4.6	none	none
none	Contig22076_at	1.07	-4.52	none	none
none	Contig8690_at	1.02	-4.45	none	none
none	HD13L20r_s_at	1.26	-4.39	none	none
none	HV_CEb0001N19r2_at	-1.1	-4.36	none	none
none	Contig5871_at	-1.17	-4.27	none	none
none	Contig23664_at	1.19	-4.04	none	none
none	HY06O21u_x_at	-1.09	-4.02	none	none
none	HV10O20u_x_at	1.08	-4.01	none	none

none	S0000200066G12F1_at	-1.15	-4	none	none
none	Contig16808_at	1.06	-3.98	none	none
none	EBpi01_SQ004_J13_at	1.07	-3.95	none	none
none	rbags11l14_at	-1.31	-3.93	none	none
none	Contig12720_at	-1.32	-3.92	none	none
none	HVSMEc0001D14f_x_at	-1.31	-3.91	none	none
none	Contig17996_s_at	-1.2	-3.88	none	none
none	HX02B15u_s_at	-1.65	-3.76	none	none
none	Contig23847_at	1.14	-3.64	none	none
none	HK04J03r_at	-1.27	-3.64	none	none
none	Contig1497_s_at	-1.22	-3.63	none	none
none	HVSMEn0016F16r2_at	-1.06	-3.59	none	none
none	Contig22399_at	-1.13	-3.57	none	none
none	HW02E10u_at	-1.06	-3.5	none	none
none	EBem10_SQ004_H02_at	-1.03	-3.48	none	none
none	HV10A20u_at	-1.47	-3.48	none	none
none	HVSMEb0012O04f_at	-1.47	-3.47	none	none
none	HZ65P03r_at	-1	-3.47	none	none
none	HW04K16u_at	-1.65	-3.46	none	none
none	HVSMEn0016A20r2_s_at	1.9	-3.45	none	none
none	HVSMEk0023G08r2_at	1.21	-3.42	none	none
none	Mla_5pri-UTR_intron2_s_at	-1.05	-3.33	none	none
none	HT10O07u_at	-1.02	-3.32	none	none
none	S0001000055C23F1_at	1.01	-3.32	none	none
none	HS07G02u_x_at	1.07	-3.29	none	none
none	Contig25774_at	1.04	-3.27	none	none
none	HT07J03u_at	1.03	-3.27	none	none
none	HW04D20u_at	-1.25	-3.25	none	none
none	Contig21437_at	1.06	-3.24	none	none
none	EBro08_SQ003_I19_s_at	-1.25	-3.23	none	none
none	HA15K23r_s_at	-1.22	-3.21	none	none
none	EBem04_SQ002_B11_x_at	-1.1	-3.16	none	none
none	Contig16984_at	-1.2	-3.12	none	none
none	Contig26110_at	-1.32	-3.12	none	none
none	HV09H13u_at	-1.12	-3.12	none	none
none	HVSMEk0006O10r2_at	-1.17	-3.12	none	none
none	Contig12877_at	-1.08	-3.1	none	none
none	Contig20098_at	1.02	-3.06	none	none
none	HVSMEh0081A14f_x_at	-1.66	-3.06	none	none
none	HV09A21u_at	-1.11	-3.05	none	none
none	Contig18195_at	-1.09	-3.04	none	none
none	Contig14643_at	1.02	-3.03	none	none
none	Contig18972_at	-1.09	-3.03	none	none
none	Contig24062_at	1.14	-3.03	none	none
none	HM04I24r_at	-1.14	-3.03	none	none
none	EBro08_SQ009_K19_at	-1.35	-3.02	none	none
none	HVSMEg0011P04r2_at	-1.07	-2.96	none	none
none	Contig25460_at	-1.26	-2.94	none	none
none	HF01J02w_at	-1.02	-2.94	none	none
none	HVSMEb0014L17f_at	-1.05	-2.94	none	none
none	Contig20649_at	1.17	-2.93	none	none
none	EBem10_SQ001_I24_s_at	-1.18	-2.93	none	none
none	HV01N13w_at	1.03	-2.91	none	none
none	HF08O15r_at	1.34	-2.88	none	none
none	HVSMEf0008K18r2_at	-1.11	-2.86	none	none
none	HT09J04u_x_at	1.92	-2.85	none	none
none	HVSMEb0007H18r2_at	-1.17	-2.83	none	none
none	HA09l22r_at	1.12	-2.81	none	none
none	HD08L09r_at	-1.17	-2.81	none	none
none	HV11I05u_x_at	-1.25	-2.81	none	none
none	Contig14848_at	1.32	-2.8	none	none
none	Contig20711_at	1.28	-2.78	none	none
none	Contig19960_s_at	-1.44	-2.77	none	none

none	Contig20699_at	-1.14	-2.76	none	none
none	HV_CEa0004H12r2_at	-1.05	-2.76	none	none
none	HY08N07u_at	-1.03	-2.75	none	none
none	Contig2580_5_at	-1	-2.72	none	none
none	Contig3483_s_at	1.28	-2.71	none	none
none	HD04F02u_at	1.19	-2.67	none	none
none	Contig16098_at	1	-2.65	none	none
none	EBro07_SQ003_A11_at	1.06	-2.65	none	none
none	HT08E01u_s_at	-1.09	-2.65	none	none
none	Contig14625_at	-1.05	-2.64	none	none
none	Contig17112_at	1.05	-2.64	none	none
none	EBro02_SQ006_H19_at	-1.12	-2.61	none	none
none	HVSMEl0023K24r2_at	-1.03	-2.61	none	none
none	Contig12289_at	-1.2	-2.59	none	none
none	Contig20120_at	1.1	-2.59	none	none
none	HVSMEa0017O08r2_at	1.01	-2.59	none	none
none	HT11E04u_x_at	-1.18	-2.57	none	none
none	HV09A21u_x_at	-1.16	-2.57	none	none
none	HV12N12u_at	1.29	-2.57	none	none
none	AF509747.1_at	-1.05	-2.56	none	none
none	Contig12584_s_at	-1.01	-2.56	none	none
none	Contig12251_at	1.04	-2.55	none	none
none	HVSMEf0020N07r2_at	1.23	-2.55	none	none
none	HS08I15u_at	-1.05	-2.54	none	none
none	EBma01_SQ005_B17_at	-1.42	-2.53	none	none
none	EBma08_SQ003_L11_at	-1.15	-2.53	none	none
none	Contig8304_at	1.01	-2.51	none	none
none	Contig15674_at	-1.12	-2.5	none	none
none	rbaal9p16_at	-1.05	-2.5	none	none
none	EBpi01_SQ004_G05_at	-1.29	-2.49	none	none
none	Contig17409_at	-1.02	-2.48	none	none
none	Contig25839_at	-1.13	-2.48	none	none
none	HA22L21r_at	1.08	-2.47	none	none
none	rbags24b14_at	1.11	-2.47	none	none
none	Contig25817_at	1.1	-2.46	none	none
none	Contig17523_at	-1.08	-2.45	none	none
none	HVSMEf0009F05r2_at	1.2	-2.45	none	none
none	Contig14299_at	1.09	-2.44	none	none
none	Contig9030_at	1.02	-2.44	none	none
none	Contig5987_at	-1.05	-2.43	none	none
none	EBed02_SQ002_L15_x_at	-1	-2.43	none	none
none	HA18M18r_x_at	-1.03	-2.43	none	none
none	rbah15o14_at	1.03	-2.43	none	none
none	EBem04_SQ002_C12_at	-1.25	-2.42	none	none
none	HVSMEf0013H13r2_at	-1.04	-2.42	none	none
none	Contig12297_at	1.07	-2.41	none	none
none	Contig15338_at	-1.06	-2.4	none	none
none	Contig22828_at	1.08	-2.4	none	none
none	Contig11309_s_at	-1.15	-2.39	none	none
none	Contig17464_at	-1.22	-2.39	none	none
none	Contig23234_at	1.04	-2.39	none	none
none	HD03N13u_at	1.15	-2.39	none	none
none	HM02H07u_at	1.05	-2.39	none	none
none	HVSMEf0014C12r2_s_at	1.41	-2.39	none	none
none	EBro02_SQ005_L23_at	1.2	-2.38	none	none
none	HVSMEm0022A07r2_at	-1.02	-2.38	none	none
none	Contig17419_at	1.09	-2.37	none	none
none	Contig24512_at	-1.29	-2.37	none	none
none	Contig2476_at	1.36	-2.37	none	none
none	Contig9703_at	1.08	-2.37	none	none
none	Contig23743_at	1.05	-2.36	none	none
none	HW01A08u_at	1.08	-2.36	none	none
none	Contig15420_at	1.05	-2.35	none	none

none	Contig24189_at	-1.16	-2.35	none	none
none	rbaal36e18_at	-1.12	-2.35	none	none
none	Contig12318_at	-1.16	-2.34	none	none
none	Contig21644_s_at	1.11	-2.33	none	none
none	HU14C15u_x_at	1.01	-2.33	none	none
none	Contig25244_at	-1.08	-2.3	none	none
none	EBro07_SQ003_D14_at	-1.17	-2.3	none	none
none	Contig22907_at	1.17	-2.28	none	none
none	EBma05_SQ003_N16_at	1.17	-2.28	none	none
none	HB30E18r_at	1.13	-2.28	none	none
none	HT07O20u_at	1.04	-2.28	none	none
none	HU10I09u_at	-1.1	-2.28	none	none
none	HVSMEg0015H12r2_s_at	-1.09	-2.28	none	none
none	HVSMEi0003G04r2_x_at	-1.11	-2.27	none	none
none	HM04D17r_at	1.3	-2.26	none	none
none	HVSMEf0019P24r2_at	-1.12	-2.26	none	none
none	Contig12978_at	1.22	-2.25	none	none
none	HD13A24r_at	1.23	-2.25	none	none
none	HK04E07r_at	1.18	-2.25	none	none
none	HVSMEm0023D15r2_at	-1.09	-2.25	none	none
none	Contig23720_at	-1.09	-2.24	none	none
none	HV12C23u_x_at	1.1	-2.22	none	none
none	Contig20050_at	1.22	-2.21	none	none
none	HVSMEi0009E02r2_at	-1.13	-2.21	none	none
none	HVSMEk0016B20r2_at	-1.17	-2.21	none	none
none	Contig13137_at	-1.15	-2.2	none	none
none	Contig21089_at	1.39	-2.2	none	none
none	EBem09_SQ001_C09_at	1.11	-2.2	none	none
none	EBem10_SQ002_A13_at	-1.15	-2.2	none	none
none	HT08E06u_x_at	-1.16	-2.2	none	none
none	Contig23166_at	-1.16	-2.19	none	none
none	Contig25027_at	1.05	-2.19	none	none
none	HV06B11u_at	-1.12	-2.19	none	none
none	HVSMEf0015C12f_at	-1.38	-2.19	none	none
none	Contig14233_at	1.07	-2.18	none	none
none	Contig18391_at	1.1	-2.18	none	none
none	Contig3940_at	-1.04	-2.18	none	none
none	EBem04_SQ003_J01_at	1.17	-2.18	none	none
none	S0000800054H06F1_x_at	1.03	-2.18	none	none
none	Contig8295_at	-1.05	-2.16	none	none
none	EBed07_SQ003_F20_at	1.18	-2.15	none	none
none	EBem05_SQ001_K06_at	-1.07	-2.14	none	none
none	HT09A05u_at	1.09	-2.14	none	none
none	HVSMEg0015E17r2_at	1.14	-2.14	none	none
none	S0000800031B05F1_at	-1.24	-2.14	none	none
none	Contig11838_at	1.17	-2.13	none	none
none	Contig19116_at	-1.12	-2.13	none	none
none	Contig22588_at	-1.11	-2.13	none	none
none	Contig3063_s_at	-1.15	-2.13	none	none
none	HA04i24r_at	-1.03	-2.13	none	none
none	HU10I18u_at	-1.83	-2.13	none	none
none	Contig4029_x_at	-1.17	-2.12	none	none
none	Contig23309_at	-1.04	-2.11	none	none
none	EBro08_SQ009_P22_at	1.08	-2.11	none	none
none	Contig21047_at	1.19	-2.1	none	none
none	EBem05_SQ002_E11_at	-1.16	-2.09	none	none
none	HVSMEn0014N12r2_x_at	1.32	-2.09	none	none
none	Contig17318_at	1.11	-2.08	none	none
none	Contig18320_at	-1.22	-2.08	none	none
none	EBem08_SQ003_N21_at	-1.16	-2.08	none	none
none	HV08M06u_at	-1.04	-2.08	none	none
none	Contig13941_at	-1.16	-2.07	none	none
none	EBpi01_SQ003_I06_at	1.03	-2.07	none	none

none	Contig20531_at	-1.05	-2.06	none	none
none	HV05O23u_at	-1.03	-2.06	none	none
none	HW06K22u_s_at	1.08	-2.06	none	none
none	HZ01L15u_at	-1.06	-2.05	none	none
none	Contig18206_at	1.14	-2.04	none	none
none	EBro08_SQ003_G24_at	-1.13	-2.04	none	none
none	EBro08_SQ009_G15_at	1.16	-2.04	none	none
none	HVSMEb0009N20r2_at	-1.02	-2.04	none	none
none	HVSMEl0006A12r2_s_at	1.19	-2.04	none	none
none	Contig24294_at	1.03	-2.03	none	none
none	Contig24342_at	1.16	-2.03	none	none
none	Contig8526_at	1.13	-2.03	none	none
none	Contig9024_s_at	1.11	-2.03	none	none
none	HT12A14r_at	0	-2.03	none	none
none	HVSMEm0005B12r2_at	-1.02	-2.03	none	none
none	Contig14383_at	1.04	-2.02	none	none
none	EBem09_SQ007_D05_x_at	-1.04	-2.02	none	none
none	HB21D19r_at	-1.01	-2.02	none	none
none	rbags20f22_at	-1.04	-2.02	none	none
none	Contig12979_s_at	1.28	-2.01	none	none
none	HZ56N02r_x_at	1.08	-2.01	none	none
none	Contig11527_at	-1.19	-2	none	none
none	Contig11899_at	-1.35	-2	none	none
none	Contig18076_at	-1.01	-2	none	none
none	EBed02_SQ002_O17_at	-1.04	-2	none	none
none	EBem05_SQ002_B05_at	-1.07	-2	none	none

* The fold change represents the mean ratio of gene expression in leaves of the two genotypes exposed to 5 μM Cd for 15 d over those in the control. Genes were considered up-regulated and down-regulated if the induction ratio was >2.0 and <-2.0, respectively.
