# Supplementary material for: Genome-wide transcriptome and functional analysis of two contrasting genotypes reveals key genes for cadmium tolerance in barley
Source: BMC Genomics. 2014 Jul 19;15(1):611. doi: 10.1186/1471-2164-15-611 (PMC4117959; doi:10.1186/1471-2164-15-611)
Supplement: Supplementary file 7 — Additional file 7: Table S6: List of genes up-regulated in both Weisuobuzhi and Dong17 after exposing the plants to 5 μM Cd for 15 d. (PDF 96 KB) [file 12864_2014_6304_MOESM7_ESM.pdf]

Additional File 7: Table S6 List of genes up-regulated in both Weisuobuzhi and Dong17 after exposing the plants to 5 μM Cd for 15 d.

Annotation	Probe Set ID	Fold change* (Cd *vs* control)		Accession No	e-value
		WS	D		
**Stress and defense response**					
Thaumatin-like protein TLP7 [*H. vulgare*]	rbaak13h13_s_at	2.68	2.01	AAK55325.1	5e-38
Gamma-thionin precursor [*H. vulgare*]	Contig381_x_at	2.06	2.1	S65779	2e-32
1-aminocyclopropane-1-carboxylate oxidase [*P. edulis*]	EBro03_SQ004_E10_at	2.31	2.13	BAB32502.1	5e-13
Methyljasmonate-inducible lipoxygenase 2 [*H. vulgare*]	Contig2306_s_at	2.93	2.39	T06190	5e-95
P450 [T. aestivum]	Contig15561_s_at	2.69	2.51	BAB87820.1	3e-63
Glutathione transferase [*H. vulgare subsp.*]	Contig20831_at	2.23	2.65	AAL73394.1	5e-60
Contains ESTs C74501, AU094804 [*O. sativa* (japonica)]	Contig10481_at	4.18	2.67	BAB90402.1	5e-47
Abscisic acid- and stress-induced protein [*O. sativa*]	Contig8961_at	3.75	3.16	T02663	9e-17
Cysteine protease component of protease-inhibitor complex [*Z. mays*]	Contig2554_at	2.44	3.46	BAA88898.1	2e-31
Probable chitinase [*H. vulgare*]	Contig25195_at	2.47	4.14	T04484	3e-53
Probable cysteine proteinase [*H. vulgare*]	Contig2988_s_at	5.54	4.15	T05920	9e-52
Barwin homolog wheatwin2 precursor [*T. aestivum*]	Contig2546_at	2.65	4.26	T06486	3e-64
18,9 KDa ABA-induced protein [*H. vulgare subsp.*]	Contig6276_s_at	4.01	4.91	T04417	5e-80
Putative class III chitinase [*O. sativa*]	Contig5024_at	5.8	7.12	AAM08776.1	4e-79
Avr9/Cf-9 rapidly elicited protein 65 [*N.tabacum*]	Contig19890_at	6.13	8.34	AAG43557.1	e-04
polyubiquitin (UBQ4) [*A. thaliana*]	Contig1058_x_at	3.31	23.47	NP_568397.1	1e-47
**Transport**					
Lipid transfer protein Cw(21) [*H. vulgare*]	rbasd16a13_s_at	2.94	2.95	S28872	9e-09
Nonspecific lipid-transfer protein 4.3 precursor [*H. vulgare subsp.*]	Contig959_x_at	2.09	2.03	Q42842	3e-39
Glu-tRNA(Gln) amidotransferase subunit A [*A. thaliana*]	Contig7597_at	2.07	2.13	AAG29095.1	9e-95
Lipid transfer protein [*A. thaliana*]	rbags1f20_x_at	2.01	2.21	2115353A	5e-07
Copper chaperone homolog CCH [*O. sativa*]	Contig6788_at	3.07	2.27	T50779	6e-29
fructosyltransferase [*Lolium perenne*]	rbah48h06_s_at	2.35	2.37	AAL92880.1	6e-84
Glycoprotein specific UDP-glucuronyltransferase [*A. thaliana*]	Contig10435_at	2.6	2.51	AAD45998.1	5e-10
Probable phospholipid transfer protein precursor [*H. vulgare*]	Contig871_s_at	2.96	2.59	T04407	7e-39
F23N19.15 similar to lipid transfer protein [*A. thaliana*]	Contig6504_s_at	2.29	2.73	AAF19544.1	1e-37
Putative serine carboxypeptidase OSJNBa0046L02.7 [*O. sativa*]	HK04D23r_at	2.59	2.73	AAL73563.1	3e-14
Putative uncharacterized protein At3g62660 [*A. thaliana*]	HVSMEc0009L17r2_at	4.27	2.99	NP_191825.2	7e-29
Putative flavonol glucosyltransferase [*O. sativa* (japonica)]	Contig11602_at	2.24	3.05	BAB68083.1	2e-51
Mitochondrial carrier protein family [*A. thaliana*]	Contig6913_x_at	2.36	3.13	NP_568060.1	7e-96
Aquaporin 2 [*Samanea saman*]	Contig19393_at	2.16	3.21	AAC17529.1	7e-57
Nonspecific lipid-transfer protein 4.1 precursor [*H. vulgare*]	Contig972_x_at	3.47	3.6	Q43767	2e-41
Herbicide safener binding protein 1 [*Z. mays*]	Contig4910_at	16.08	4.11	T01354	4e-62
Probable lipid transfer protein [*H. vulgare*]	Contig6042_at	4.17	4.25	T06199	3e-41
Putative protein; protein id: At5g14910.1 [*A. thaliana*]	Contig10306_s_at	2.78	4.74	NP_568306.1	3e-23
Trigger factor-like protein; protein id: At5g55220.1, [*A. thaliana*]	Contig7069_at	2.27	4.85	NP_200333.2	2e-79
Putative protein; protein id: At5g14910.1 [*A. thaliana*]	Contig10306_at	2.96	5.17	NP_568306.1	3e-23
Herbicide safener binding protein 1 [*Z. mays*]	Contig4911_at	12.6	8.32	T01354	1e-60
Putative seven transmembrane protein [*O. sativa* (japonica)]	HVSMEb0006O01r2_at	2.14	44.8	BAB92639.1	2e-06
Putative fatty acid condensing enzyme CUT1 [O. sativa (japonica)]	HVSMEb0010E16r2_s_at	2.46	2.3	BAB91850.1	1e-25
THA4 [*Z. mays*]	Contig18499_at	2.04	2.5	AAD31522.1	5e-18
**Transcription**					
At1g02150/T7I23.8 [*A. thaliana*]	Contig6600_at	2.11	2.12	AAM19786.1	2e-50
Probable RNA-binding protein cp33 precursor [*H. vulgare subsp.*]	Contig7834_at	2.28	2.48	T05730	e-153
Contains ESTs D47958 [*O. sativa* (japonica)]	Contig6169_at	2.37	2.59	BAB78620.1	2e-35
Putative nascent polypeptide associated complex alpha chain [*O. sativa* (japonica)]	Contig11109_at	3.15	4.03	AAM52321.1	2e-46
F22G5.34 [*A. thaliana*]	Contig9938_at	2.17	4.14	AAF79563.1	5e-78
Transducin-like enhancer protein 4 [*Mus musculus*]	Contig13990_at	2.06	5.19	Q62441	0.029
Contains ESTs D47958 [*O. sativa* (japonica)]	Contig6170_s_at	3.12	2.81	BAB78620.1	4e-30
Putative mitotic control protein dis3 [*O. sativa* (japonica)]	HV_CEb0018G22r2_at	2	3.75	AAN17392.1	2e-31
**Carbohydrate metabolism**					
Putative purple acid phosphatase [*O. sativa (japonica)*]	Contig26476_at	2.83	2.06	BAC07354.1	6e-20
Fatty acyl coA reductase [*T. aestivum*]	Contig10274_at	2.52	2.18	CAD30694.1	1e-59
Probable vacuolar hydrolase, beta-fructosidase [*T. aestivum*]	Contig2490_at	3.7	2.31	T06338	e-131
Beta-D-xylosidase [*H. vulgare*]	Contig13674_at	2.78	2.34	AAK38482.1	1e-80
Putative arabinogalactan-like protein [*O. sativa (japonica)*]	Contig6782_at	3.03	2.36	BAB84493.1	3e-23

Putative NAD dependent epimerase [*A. thaliana*]	Contig16221_at	2.71	2.38	NP_189024.1	8e-55
Endo-1,4-beta-glucanase Cel1 [*H. vulgare subsp.*]	HVSMEn0019D12r2_s_at	3.25	2.52	BAA94257.1	2e-10
RuBisCO large subunit-binding protein subunit beta, chloroplastic [*Secale cereale*]	Contig807_at	2.79	2.75	Q43831	4e-98
Glucan endo-1,3-beta-glucosidase [*H. vulgare subsp.*]	Contig1632_at	2.31	2.8	AAA32960.1	e-169
Putative xylanase inhibitor protein [*O. sativa* (japonica)]	Contig5996_s_at	3.42	3.06	BAC10141.1	1e-30
Xyloglucan endo-1,4-beta-D-glucanase [*H. vulgare subsp.*]	HVSMEb0004L16r2_at	4.63	3.54	T06201	1e-64
Xyloglucan endo-1,4-beta-D-glucanase [*H. vulgare subsp.*]	Contig2670_x_at	7.42	3.67	T06201	e-154
Putative acid phosphatase [*H. vulgare subsp.*]	Contig2433_s_at	2.48	4.73	CAB71336.1	e-112
Apyrase GS52 [*Glycine soja*]	Contig3332_at	3.88	6.52	AAG32960.1	4e-55
RuBisCO subunit binding-protein alpha subunit, chloroplast precursor [*S. cereale*]	rbags36a18_s_at	6.46	11	P08823	5e-08
**Cell growth, division**					
Porphobilinogen deaminase [*T. aestivum*]	Contig5956_at	2.1	2.16	AAL12220.1	e-122
Expansin [Festuca pratensis]	Contig3675_at	4.48	2.4	CAC06433.1	e-92
23 kDa jasmonate-induced protein 1 [*H. vulgare*]	Contig1679_s_at	4.52	3.23	P32024	8e-93
**Fat metabolism**					
Similar to latex allergen from Hevea brasiliensis [*A. thaliana*]	Contig4196_at	2.82	2.85	AAM63157.1	2e-53
GDSL-motif lipase/hydrolase-like protein [*A. thaliana*]	Contig15_s_at	3.4	3.05	NP_200316.1	3e-15
Allene oxide synthase [*H. vulgare subsp.*]	Contig3096_s_at	3.48	3.46	CAB86384.1	e-121
Similar to lipases [*A. thaliana*]	Contig6611_at	4.26	10.74	AAF63138.1	e-71
**Photosynthesis**					
Coproporphyrinogen III oxidase, chloroplast precursor [*H. vulgare*]	Contig5401_s_at	2.35	2.36	Q42840	e-110
Putative protoporphyrinogen IX oxidase [O. sativa (japonica)]	Contig7919_at	2.23	3.12	BAB39998.1	e-115
Chloroplast Cpn21 protein [*A. thaliana*]	Contig3840_at	2.57	3.65	NP_197572.1	5e-87
Chlorophyll a/b-binding protein WCAB precursor [*T. aestivum*]	Contig949_at	2.1	11.44	AAB18209.1	e-125
**Protein synthesis**					
Strictosidine synthase-related protein [*A. thaliana*]	Contig9052_at	2.31	2.04	NP_563818.1	1e-83
Chloroplast 50S ribosomal protein L2 [*H. vulgare*]	ChlorContig17_s_at	2.09	2.75	P41096	e-129
50S ribosomal protein L24, chloroplast precursor [*A. thaliana*]	Contig6148_at	2.01	2.86	NP_200271.1	3e-55
50S ribosomal protein L12-1, chloroplast precursor [*S. cereale*]	Contig8125_at	2.04	3.24	Q06030	7e-46
30S ribosomal protein S17, chloroplast precursor [*O. sativa*]	Contig4490_s_at	2.08	3.41	Q9ZST1	6e-45
50S ribosomal protein L5 [*O. sativa*]	Contig5775_at	2.23	3.49	AAC64970.1	e-106
Ribosomal protein L29 [*Z. mays*]	Contig9437_at	2.88	3.61	AAD50383.1	2e-50
Plastid ribosomal protein L11 [*O. sativa* (japonica)]	Contig8084_at	2.57	3.72	BAB21483.1	6e-77
RuBisCO subunit binding-protein beta subunit [*S. cereale*]	Contig807_s_at	2.61	3.9	Q43831	4e-98
Putative ribonucleoprotein [*O. sativa*]	Contig5988_at	3.81	4	AAL82527.1	6e-76
Ribosomal protein L12.1 precursor, chloroplast [*S. cereale*]	Contig12793_at	2.38	4.03	S30199	2e-53
Expressed protein; protein id: At4g29060.1, [*A. thaliana*]	Contig3659_s_at	2.67	4.13	NP_567820.1	2e-54
Expressed protein; protein id: At4g29060.1, [*A. thaliana*]	Contig3659_at	2.37	4.56	NP_567820.1	2e-54
Putative ribosomal protein L18 [*O. sativa*]	Contig5585_s_at	2.6	4.58	AAL79739.1	5e-62
Plastid-specific ribosomal protein 2 precursor [*S. oleracea*]	Contig9436_at	2.89	4.83	AAF64167.1	3e-50
Putative elongation factor P [*A. thaliana*]	Contig17155_at	2.84	5.99	NP_566333.1	5e-74
Adenosine diphosphate glucose pyrophosphatase [*H. vulgare subsp.*]	Contig2769_s_at	15.54	42.64	CAC32847.1	4e-72
**Signal transduction**					
33 kDa secretory protein [*O. sativa*]	Contig20580_at	2.04	2.09	AAC36744.1	4e-40
Putative leucine-rich repeat transmembrane protein kinase [*A. thaliana*]	Contig13334_at	2.92	2.15	NP_192248.1	3e-63
Unknown classified					
(AY088630) unknown [*A. thaliana*]	Contig18643_at	2	2.39	AAM66952.1	3e-15
Hypothetical protein [*O. sativa* (japonica)]	Contig17412_at	2.14	2.42	BAB89418.1	5e-33
Putative protein [*A. thaliana*]	Contig5364_at	2.01	2.42	NP_194537.1	2e-45
Hypothetical protein [*Anabaena* sp. (strain PCC 7120)]	Contig6063_s_at	2.28	2.42	NP_487053.1	9e-34
Putative protein [*A. thaliana*]	Contig5475_s_at	2.65	2.43	NP_568160.1	1e-23
Hypothetical protein [*O. sativa*]	Contig504_x_at	2.03	2.48	AAG46123.1	5e-38
Unnamed protein product [*O. sativa* (japonica)]	Contig228_s_at	2.55	2.64	BAA96147.1	9e-67
Hypothetical protein [*O. sativa* (japonica)]	Contig10379_at	2.67	2.78	BAB16875.1	8e-07
Expressed T31J12.3 protein [A. thaliana]	Contig6804_at	4.79	2.83	NP_563841.1	9e-37
Expressed protein [*A. thaliana*]	Contig18991_at	2.61	3.55	NP_680449.1	4e-17
Hypothetical protein [*O. sativa* (japonica)]	Contig7338_at	2.05	3.56	AAL58119.1	1e-43
Putative protein [*A. thaliana*]	Contig20033_at	2.8	3.65	NP_198202.1	2e-19
Unknown protein [*O. sativa*]	Contig13271_at	3.01	3.81	AAL58188.1	e-103
Expressed protein [*A. thaliana*]	Contig5487_s_at	4.65	3.87	NP_564683.1	1e-19
P0520B06.12 [*O. sativa* (japonica)]	Contig7279_at	2.98	4.42	BAB92188.1	2e-86
Expressed protein [*A. thaliana*]	HVSMEm0006I15r2_at	2.03	5.04	NP_566739.1	2e-27
P0520B06.12 [*O. sativa* (japonica)]	HVSMEg0001H24r2_s_at	2.41	5.24	BAB92188.1	6e-13

OSJNBa0086B14.7 [*O. sativa* (japonica)]	Contig11993_at	2.08	5.27	CAD40835.1	4e-12
Expressed protein [*A. thaliana*]	HVSMEg0010M12r2_at	2.33	7.54	NP_565093.1	3e-37
Putative protein [*A. thaliana*]	Contig5704_at	3.63	8.38	NP_190439.1	7e-44
unknown protein [*O. sativa* (japonica)]	Contig8346_at	3.9	10.16	BAC10351.1	e-113
OSJNBb0066J23.1 [*O. sativa* (japonica)]	Contig16214_at	14.32	10.93	CAD40597.1	3e-52
Unnamed protein product [*O. sativa* (japonica)]	Contig6075_at	22.66	63.82	BAA94780.1	2e-76
Putative protein [*A. thaliana*]	Contig10346_at	2.22	2.58	NP_190483.1	2e-43
**None**					
none	HR01A18u_x_at	2.97	2.11	none	none
none	Contig5365_s_at	2.11	2.14	none	none
none	HK06M02r_at	2.94	2.14	none	none
none	HO10P07S_at	2.04	2.15	none	none
none	HVSMEb0002K02r2_s_at	3.96	2.17	none	none
none	HE01D12u_at	2.9	2.28	none	none
none	HA10M12u_s_at	2.02	2.32	none	none
none	EBro07_SQ002_G23_s_at	2.11	2.44	none	none
none	Contig6477_s_at	2.24	2.5	none	none
none	Contig6701_s_at	2.79	2.5	none	none
none	Contig2894_s_at	2.2	2.53	none	none
none	rbaal10d03_x_at	2.36	2.58	none	none
none	HV08L20u_x_at	3.26	2.59	none	none
none	HVSMEa0011H12r2_x_at	2.18	2.85	none	none
none	HVSMEm0003O11r2_at	2.26	2.93	none	none
none	Contig5807_s_at	7.22	3.11	none	none
none	Contig4031_x_at	2.16	3.13	none	none
none	HV_CEa0015F23r2_at	2.5	3.47	none	none
none	HW09O19u_x_at	2.64	3.51	none	none
none	HV_CEb0009D09r2_at	3.09	3.55	none	none
none	Contig12100_at	2.33	3.73	none	none
none	HT09C05u_at	2.42	3.77	none	none
none	HVSMEl0006H21r2_at	2.25	3.82	none	none
none	Contig16541_at	3	3.85	none	none
none	Contig25667_s_at	2.85	3.9	none	none
none	Contig2499_s_at	8.34	4.6	none	none
none	HVSMEa0019B22r2_x_at	2.85	4.63	none	none
none	Contig12421_at	7.5	5.32	none	none
none	Contig3122_at	2.02	5.49	none	none
none	Contig18518_at	3.58	5.7	none	none
none	basd23g06_s_at	3.09	7.17	none	none
none	Contig3082_s_at	5.49	10.09	none	none
none	Contig13115_at	3.31	11.72	none	none
none	Contig19291_at	6.38	16.45	none	none
none	HS06L16u_x_at	7.26	19.37	none	none
none	HVSMEm0001F11r2_x_at	6.15	21.16	none	none
none	HVSMEa0019B22f_x_at	3.92	52.91	none	none

* The fold change represents the mean ratio of gene expression in leaves of the two genotypes exposed to 5 μM Cd for 15 d over those in the control. Genes were considered up-regulated and down-regulated if the induction ratio was >2.0 and <-2.0, respectively.
