# Supplementary material for: Genome-wide transcriptome and functional analysis of two contrasting genotypes reveals key genes for cadmium tolerance in barley
Source: BMC Genomics. 2014 Jul 19;15(1):611. doi: 10.1186/1471-2164-15-611 (PMC4117959; doi:10.1186/1471-2164-15-611)
Supplement: Supplementary file 8 — Additional file 8: Table S7: List of genes down-regulated in Weisuobuzhi and up-regulated in Dong17 after exposing the plants to 5 μM Cd for 15 d. (PDF 70 KB) [file 12864_2014_6304_MOESM8_ESM.pdf]

Additional File 8: Table S7 List of genes down-regulated in Weisuobuzhi and up-regulated in Dong17 after exposing the plants to 5 μM Cd for 15 d.

Annotation	Probe Set ID	Fold change* (Cd *vs* control)		Accession No	E-value
		W	D		
**Stress and defense response**					
Peroxidase [*T. aestivum*]	HVSMEm0005P05r2_at	-3.51	7.02	S14611	2e-23
Pathogenesis related protein-1 [*Z. mays*]	Contig12046_at	-3.87	6.9	T02054	5e-57
Pathogenesis-related protein 4 [*H. vulgare*]	Contig2550_x_at	-3.11	4.95	T06169	8e-77
Putative calreticulin [*O. sativa* (japonica)]	rbags16i08_x_at	-2.06	4.26	AC06263.1	7e-11
Pathogenesis-related protein 1a [*H. vulgare subsp.*]	Contig2214_s_at	-5.47	4.09	S37166	5e-81
Permatin homolog PR5 [*H. vulgare subsp.*]	Contig2787_s_at	-4.62	3.76	T05973	e-133
Pathogenesis-related protein PRB1-3 precursor[*H. vulgare*]	Contig2212_s_at	-6.6	3.75	P35793	8e-88
Pathogenesis-related protein 1 precursor [*H. vulgare*]	Contig2210_at	-5.42	3.65	Q05968	2e-80
Pathogenesis-related protein 1a precursor [*H. vulgare*]	Contig4056_s_at	-5.08	3.47	P32937	7e-46
Thaumatin-like protein TLP7 [*H. vulgare*]	Contig2789_at	-6.27	3.44	AAK55325.1	e-117
Pathogenesis-related protein 1a [*H. vulgare*]	Contig2209_at	-4.24	3.26	S37166	5e-81
Pathogenesis-related protein 4 [*H. vulgare*]	Contig639_at	-8.85	2.87	T06171	4e-12
Thaumatin-like protein TLP8 [*H. vulgare*]	EBem10_SQ002_I10_s_at	-3.14	2.68	AAK55326.1	8e-04
Pathogen-induced protein WIR1A [*T. aestivum*]	Contig5974_s_at	-3.19	2.51	Q01482	1e-24
Pathogenesis-related protein PRB1-2 precursor [*H. vulgare*]	Contig2211_at	-3.89	2.35	P35792	1e-80
WIR1 protein [*T. aestivum*]	Contig9917_at	-2.01	2.19	S55368	1e-04
**Transcription**					
Myb-related protein [*H. vulgare*]	Contig3667_s_at	-3.57	3.77	T06179	e-164
**Carbohydrate metabolism**					
Glucan endo-1,3-beta-D-glucosidase [*H. vulgare*]	Contig1637_s_at	-7.24	8.54	D38664	e-162
Glucan endo-1,3-beta-D-glucosidase [*H. vulgare*]	Contig1637_at	-7.83	7.94	D38664	e-162
Glucan endo-1,3-beta-D-glucosidase [*H. vulgare*]	HVSMEm0003C15r2_s_at	-6.67	7.65	A31800	2e-48
Beta-1,3-glucanase precursor [*T. aestivum*]	Contig13350_at	-4.85	6.69	AAD28734.1	7e-65
**Signal transduction**					
SERK2 protein [*Z. mays*]	Contig3635_s_at	-5.54	2.2	CAC37639.1	3e-59
SERK2 protein [*Z. mays*]	Contig3636_at	-5.73	2.06	CAC37639.1	8e-60
secretory protein [*T. aestivum*]	Contig358_at	-3.62	2.04	AAD46133.1	e-100
**Unknown classified**					
Hypothetical protein pBH6-17 [*H. vulgare*]	Contig590_s_at	-3.64	3	T06205	4e-80
Hypothetical protein pBH6-12 [*H. vulgare*]	Contig634_at	-3.07	2.33	T06204	e-120
Putative protein [*A. thaliana*]	Contig10152_at	-2.1	2.29	NP_193034.1	3e-59
Hypothetical protein [*O. sativa*]	Contig4815_at	-2.64	2.02	AAK98749.1	3e-20
OSJNBa0052O21.28 [*O. sativa* (japonica)]	Contig13248_at	-2.25	2.01	CAD40043.1	1e-82
Hypothetical protein pBH6-17 [*H. vulgare*]	Contig590_at	-2.78	2	T06205	4e-80
**None**					
none	HW06E20u_x_at	-7.38	5.31	none	none
none	Contig11773_at	-3.28	4.43	none	none
none	HP01B09w_at	-2.9	2.42	none	none
none	Contig16886_at	-6.62	2.24	none	none

* The fold change represents the mean ratio of gene expression in leaves of the two genotypes exposed to 5 μM Cd for 15 d over those in the control. Genes were considered up-regulated and down-regulated if the induction ratio was >2.0 and <-2.0, respectively.
