# Supplementary material for: Genome-wide transcriptome and functional analysis of two contrasting genotypes reveals key genes for cadmium tolerance in barley
Source: BMC Genomics. 2014 Jul 19;15(1):611. doi: 10.1186/1471-2164-15-611 (PMC4117959; doi:10.1186/1471-2164-15-611)
Supplement: Supplementary file 9 — Additional file 9: Table S8: List of genes down-regulated in both Weisuobuzhi and Dong17 after exposing the plants to 5 μM Cd for 15 d. (PDF 62 KB) [file 12864_2014_6304_MOESM9_ESM.pdf]

Additional File 9: Table S8 List of genes down-regulated in both Weisuobuzhi and Dong17 after exposure to 5 μM Cd for 15 d.

Annotation	Probe Set ID	Fold change* (Cd *vs* control)		Accession No	E-value
		W	D		
**Stress and defense response**					
Glutathione-S-transferase Cla47 [*T. aestivum*]	Contig9764_at	-2.17	-2.03	AAL47687.1	7e-55
Pathogenesis-related protein PR-10a [*O. sativa*]	Contig4402_s_at	-4	-2.07	AAF85972.1	6e-29
Superoxide dismutase (Cu-Zn) [*T. aestivum*]	Contig3197_at	-3.31	-2.77	T06800	9e-83
Oxylase-like protein [*A. thaliana*]	Contig5345_s_at	-2.19	-5.52	NP_566623.1	9e-71
**Transcription**	Contig1169_at	-2.82	-2.36	S56685	9e-50
Histone H2B-8 [*T. aestivum*]	Contig23505_at	-2.04	-3.43	NP_113612.1	0.005
Chromosome 20 open reading frame 55 [*H. sapiens*]	Contig18878_at	-2.11	-2.01	NP_177928.1	3e-18
Carbohydrate metabolism					
Phosphoglycerate mutase [*A. thaliana*]					
**Unknown classified**					
Unnamed protein product [*H. sapiens*]	MitoContig10_x_at	-2.71	-3.12	BAB71593.1	0.6
Unknown protein [*O. sativa* (japonica)]	Contig10642_at	-2.04	-3.42	BAC10843.1	8e-40
**None**					
none	Contig23342_at	-2.57	-2.22	none	none

* The fold change represents the mean ratio of gene expression in leaves of the two genotypes exposed to 5 μM Cd for 15 d over those in the control. Genes were considered up-regulated and down-regulated if the induction ratio was >2.0 and <-2.0, respectively.
