# Supplementary material for: Genome-wide transcriptome and functional analysis of two contrasting genotypes reveals key genes for cadmium tolerance in barley
Source: BMC Genomics. 2014 Jul 19;15(1):611. doi: 10.1186/1471-2164-15-611 (PMC4117959; doi:10.1186/1471-2164-15-611)
Supplement: Supplementary file 10 — Additional file 10: Table S9: List of genes down-regulated in Weisuobuzhi and not changed in Dong17 after exposing the plants to 5 μM Cd for 15 d. (PDF 77 KB) [file 12864_2014_6304_MOESM10_ESM.pdf]

Additional File 10: Table S9 List of genes down-regulated in Weisuobuzhi and not changed in Dong17 after exposure to 5 μM Cd for 15 d.

Annotation	Probe Set ID	Fold change* (Cd *vs* control)		Accession No	E-value
		W	D		
**Stress and defense response**					
Multidrug resistance protein 1 homolog [*T. aestivum*]	Contig21298_at	-3.28	1.96	BAB85651.1	6e-90
Proteinase inhibitor-related protein bsi1 precursor [*H. vulgare*]	HD07M22r_s_at	-4.35	1.91	S53102	2e-29
Oxalate oxidase [*H. vulgare*]	Contig3017_at	-3.7	1.84	CAA74595.1	e-126
Barwin homolog wheatwin2 precursor [*T. aestivum*]	HT07J20u_x_at	-2.55	1.7	T06486	2e-06
Pathogen-induced protein WIR1A [*T. aestivum*]	Contig23878_x_at	-2.01	1.62	Q01482	0.001
P450 [T. aestivum]	Contig26116_at	-2.64	1.6	BAB87820.1	5e-43
Alternative oxidase [*O. sativa* (japonica)]	Contig15936_at	-2.66	1.59	BAA28772.1	6e-21
Osmotin-like protein [*O. sativa* (japonica)]	Contig9094_at	-2.02	1.54	BAB67891.1	2e-63
Formate dehydrogenase (FDH) [*H. vulgare*]	HVSMEa0019P15r2_at	-4.1	1.52	Q9ZRI8	1e-41
Oxalate oxidase 2 [*H. vulgare*]	Contig3018_at	-3.22	1.48	P45851	e-109
Pathogenesis-related protein 4 [*H. vulgare*]	Contig15099_s_at	-2.84	1.46	T06171	3e-06
Pathogenesis-related protein PR-10a [*O. sativa*]	Contig4405_x_at	-2.28	1.44	AAF85972.1	6e-36
Putative glutathione S-transferase [*O. sativa* (japonica)]	Contig14304_at	-2.49	1.26	AAM12330.1	9e-70
Probable oxalate oxidase [*O. sativa*]	Contig10860_at	-2.15	1.21	T02923	2e-41
Embryo-abundant protein EMB [*P. sativum*]	Contig22839_at	-2.09	1.21	AAM19356.1	2e-15
Putative glutathione S-transferase [*O. sativa* (japonica)]	Contig21026_at	-2.2	1.08	BAB39941.1	1e-66
Probenazole-induced protein [*O. sativa* (japonica)]	HD08F14r_x_at	-5.04	1.07	T02973	1e-19
Pathogen-related protein [*H. vulgare*]	Contig5607_s_at	-2.17	1.07	P16273	e-128
Alternative oxidase AOX3 precursor [*Z. mays*]	Contig5887_at	-2.6	1	AAL27797.1	3e-43
ESTs correspond to a region of the predicted gene [*O. sativa* (japonica)]	Contig3568_at	-2.41	-1.03	BAA95828.1	7e-86
Putative glutathione S-transferase [*O. sativa* (japonica)]	HV_CEb0004O15r2_s_at	-3.16	-1.08	AAM12330.1	7e-08
DNAJ protein - like [*A. thaliana*]	Contig11141_at	-2.16	-1.09	NP_195923.1	1e-37
Glutathione S-transferase GST 24 [*Z. mays*]	Contig15264_at	-2.41	-1.34	AAG34832.2	8e-72
ESTs correspond to a region of the predicted gene [O. sativa (japonica)]	Contig3563_at	-2.06	-1.34	BAA95828.1	2e-47
Pathogenesis-related protein PR-10a [*O. sativa*]	Contig4406_x_at	-2.95	-1.43	AAF85972.1	2e-38
safener-induced In2.1-like protein [*T. aestivum*]	Contig6546_at	-2.99	-1.55	T06480	e-101
Zinc-induced protein-like [O. sativa (japonica)]	Contig14507_at	-2.02	-1.56	BAB90336.1	2e-12
**Transport**					
Phosphate/phosphoenolpyruvate translocator, plastid [*N. tabacum*]	Contig24832_at	-2.05	-1.73	T03836	5e-32
**Transcription**					
Histone H2B.2 [*T. aestivum*]	Contig1179_at	-2.38	1.88	P05621	5e-44
histone H2A.9 [*T. aestivum*]	Contig286_s_at	-2.45	-1.29	S53519	7e-51
**Carbohydrate metabolism**					
Putative cyanate hydratase [*O. sativa*]	Contig13114_at	-4.65	-1.19	AAG21913.1	7e-08
Nitrogen metabolism					
Putative indole-3-glycerol phosphate synthase [*A. thaliana*]	Contig6407_at	-2.86	1.79	AAM64536.1	3e-75
Putative indole-3-glycerol phosphate synthase [*A. thaliana*]	Contig6407_s_at	-3.99	1.63	AAM64536.1	3e-75
Putative tryptophan synthase alpha [*Z. mays*]	Contig5542_at	-3.57	1.36	AAG42689.1	1e-65
Anthranilate synthase alpha 2 subunit [*O. sativa* (japonica)]	HY07P02u_at	-2.69	-1.05	BAA82095.1	1e-67
Putative phosphoglycerate dehydrogenase [*O. sativa* (indica)]	Contig5494_at	-2.96	-1.93	CAC09348.1	3e-91
**Signal transduction**					
Putative protein kinase [*A. thaliana*]	Contig9408_at	-3.19	1.35	NP_566689.1	5e-45
Putative protein kinase Xa21 [*O. sativa* (japonica)]	Contig11866_at	-2.46	1.02	BAC10827.1	2e-54
Putative leucine rich repeat containing protein kinase [*O. sativa* (japonica)]	Contig24926_at	-2.96	-1.17	BAC10698.1	4e-13
**Unknown classified**					
Hypothetical protein [*S. pombe*]	HVSMEl0005L18r2_at	-2.11	1.52	NP_596583.1	5e-22
Unknown protein [*O. sativa*]	Contig12084_at	-2.66	1.5	AAF34415.1	9e-06
Unknow protein AT4g17280/dl4675c [*A. thaliana*]	HV_CEb0020C01r2_at	-2.53	1.28	AAL57706.1	9e-06
Putative protein [*A. thaliana*]	HVSMEa0011L14r2_s_at	-2.08	1.03	NP_193723.1	7e-48
Expressed protein [*A. thaliana*]	Contig15773_at	-2.26	-1.17	NP_565890.1	3e-06
OSJNBb0072N21.4 [*O. sativa* (japonica)]	HVSMEb0010O13f2_at	-4.82	-1.27	CAD39838.1	7e-30
B1131B07.13 [*O. sativa* (japonica)]	Contig10168_at	-2.75	-1.35	BAB93351.1	1e-23
Unknown protein [*O. sativa*]	Contig13994_s_at	-2.42	-1.39	AAG16855.1	5e-12
Putative protein [*A. thaliana*]	Contig7415_at	-3.07	-1.42	NP_193723.1	e-103
OsNAC4 protein [*O. sativa*]	Contig3362_at	-2.06	-1.51	BAA89798.1	5e-78
Unknown protein [*A. thaliana*]	Contig9868_at	-2.08	-1.63	AAM97103.1	5e-51

**None**					
none	HD04G07u_s_at	-3.43	1.91	none	none
none	Contig1159_s_at	-2.01	1.55	none	none
none	Contig1185_at	-2.45	1.53	none	none
none	S0000200070D03F1_at	-4.79	1.39	none	none
none	Contig13632_at	-2.34	1.23	none	none
none	HK03J12r_at	-2.33	1.19	none	none
none	Contig17960_at	-2.36	1.11	none	none
none	HK05D22r_x_at	-2.29	-1.14	none	none

* The fold change represents the mean ratio of gene expression in leaves of the two genotypes exposed to 5 μM Cd for 15 d over those in the control. Genes were considered up-regulated and down-regulated if the induction ratio was >2.0 and <-2.0, respectively.
