# Supplementary material for: Genome-wide transcriptome and functional analysis of two contrasting genotypes reveals key genes for cadmium tolerance in barley
Source: BMC Genomics. 2014 Jul 19;15(1):611. doi: 10.1186/1471-2164-15-611 (PMC4117959; doi:10.1186/1471-2164-15-611)
Supplement: Supplementary file 11 — Additional file 11: Table S10: List of genes not changed in Weisuobuzhi and up-regulated Dong17 after exposing the plants to 5 μM Cd for 15 d. (PDF 185 KB) [file 12864_2014_6304_MOESM11_ESM.pdf]

Additional File 11: Table S10 List of genes not changed in Weisuobuzhi and up-regulated Dong17 after exposing the plants to 5 μM Cd for 15 d.

Annotation	Probe Set ID	Fold change* (Cd *vs* control)		Accession No	E-value
		W	D		
**Stress and defense response**					
Chitinasecht2b precursor [*H. vulgare*]	Contig2992_s_at	-1.61	2.01	S48848	e-133
Putative metalloproteinase [*O. sativa* (japonica)]	Contig25510_at	-1.1	2.02	AAK55453.1	1e-13
Allene oxide synthase [*H. vulgare*]	Contig3097_at	-1.46	2.02	CAB86383.1	e-138
Putative cytochrome P-450 like protein [*O. sativa*]	Contig17320_at	-1.05	2.04	AAK92612.1	2e-53
Senescence-associated protein (SAG29) [*A. thaliana*]	Contig13823_at	1.2	2.05	AAM65389.1	5e-58
Thaumatin-like protein [*T. aestivum*]	Contig2792_s_at	1.9	2.05	AAM15877.1	e-101
Class III chitinase [*O. sativa*]	Contig5023_at	-1.97	2.06	AAG02504.1	2e-96
Alpha-hordothionin precursor [*H. vulgare*]	HB29O17r_x_at	1.22	2.06	JA0087	3e-07
Similar to Prunus armeniaca ethylene-forming-enzyme-like dioxygenase [*O. sativa* (japonica)]	HVSMEa0016D02r2_at	-1.01	2.07	BAA95829.1	9e-16
HSP80-2 [T. aestivum]	Contig1204_s_at	-1.01	2.08	CAA67191	e-137
Cytochrome b5 reductase [*Z. mays*]	Contig3972_at	1.8	2.09	AAD17694.1	e-107
Wheat aluminum induced protein wali 3[*T. aestivum*]	Contig4750_at	-1.17	2.1	JQ2360	1e-43
Chitinase cht2a precursor [*H. vulgare*]	Contig2990_at	-1.62	2.12	S48847	e-134
Fatty acid hydroperoxide lyase [*H. vulgare*]	Contig12786_at	-1.7	2.13	CAC82980.1	3e-20
Late embryogenesis abundant protein B19.1A [*H. vulgare*]	Contig1830_s_at	1.12	2.15	Q05190	7e-38
Oxalate oxidase [*T. aestivum*]	Contig1518_at	-1.37	2.17	AAF34811.1	e-117
Wheat aluminum induced protein wali 5 [*T. aestivum*]	Contig2243_s_at	-1.91	2.19	JQ2361	8e-40
Pathogenesis-related protein 4 [*H. vulgare*]	Contig6576_s_at	-1.69	2.2	T06169	1e-50
Senescence-associated protein 5 [*Hemerocallis hybrid cultivar*]	HA28N02r_at	1.27	2.2	AAC34855.1	6e-22
Putative Cytochrome P450 [*O. sativa*]	Contig1522_at	-1.85	2.21	AAM08560.1	2e-50
FKBP-type peptidyl-prolyl cis-trans isomerase; [*A. thaliana*]	Contig12695_at	-1.01	2.22	NP_181884.1	4e-58
AT4g39710/T19P19_100 [*A. thaliana*]	Contig21540_at	1.09	2.23	AAK97696.1	7e-05
Low-molecular-weight heat shock protein [*Cuscuta japonica*]	Contig15445_at	1.49	2.24	BAA33062.1	3e-25
Peroxidase [*O. sativa*]	Contig2115_at	1.18	2.25	AAC49819.1	1e-79
FKBP-type peptidyl-prolyl cis-trans isomerase [*A. thaliana*]	Contig19209_at	1.05	2.26	NP_173504.1	7e-39
Cytochrome b-c1 complex subunit 8 [*S. pombe*]	EBro08_SQ005_L04_at	1	2.26	NP_594714.1	e-18
Phenylalanine ammonia-lyase [*H. vulgare*]	Contig1800_s_at	1.02	2.29	T05966	e-120
Peroxidase precursor, pathogen-induced [*H. vulgare*]	Contig2118_at	-1.74	2.29	T06172	e-154
P450 [T. aestivum]	HVSMEb0020I08f_at	1.53	2.32	BAB87820.1	4e-26
(AJ299254) putative DNAJ protein [*N. tabacum*]	S0001100180A07F1_at	-1	2.32	CAC12824.1	2e-20
Peroxidase, putative [*A. thaliana*]	HV_CEa0004N03r2_at	1.19	2.33	NP_181373.1	0.005
Fetal Alzheimer antigen [*H. sapiens*]	rbags24a10_at	-1.02	2.34	NP_004450.1	2e-38
(AF250937) germin E [*H. vulgare*]	AF250937_s_at	-1.5	2.35	AAG00429.1	e-104
Phenylalanine ammonia-lyase [*H. vulgare*]	Contig1805_s_at	1.15	2.35	T05966	e-107
(AF387866) peroxidase [*T. aestivum*]	rbah13p07_s_at	-1.18	2.36	AAM76682.1	2e-24
Putative subtilisin-like protease [*O. sativa* (japonica)]	Contig20186_at	1.05	2.38	BAB91889.1	1e-23
Peptidyl-prolyl isomerase protein [*A. thaliana*]	Contig4537_s_at	1.54	2.38	NP_196816.1	4e-71
CI2E [H. vulgare]	AF427791_CDS-25_at	-1.24	2.39	AAM22827.1	2e-34
Putative cytochrome P450 [*O. sativa* (japonica)]	Contig18990_at	-1.71	2.4	BAB39252.1	2e-40
Chymotrypsin inhibitor 2 [*H. vulgare*]	Contig223_s_at	1.07	2.47	S18818	1e-39
Putative bromelain-like thiol protaease [*O. sativa* (japonica)]	EBro08_SQ006_H08_at	1.02	2.48	BAB63883.1	3e-18
Putative heat shock protein [*A. thaliana*]	Contig4554_at	1.89	2.49	AAM65596.1	3e-46
Putative disease resistance gene [*O. sativa* (japonica)]	Contig22732_at	-1.03	2.5	AAN08649.1	1e-44
Pathogenesis-related group 5 protein [*A. thaliana*]	Contig10686_s_at	1.09	2.52	NP_173261.1	4e-57
CI2E [H. vulgare]	HP01E21w_at	1.68	2.52	AAM22827.1	2e-14
Similar to Babesia aldo-keto reductase [*A. thaliana*]	Contig9187_at	1.24	2.54	AAB70433.1	5e-94
Bundle sheath defective protein 2 [*Z. mays*]	Contig9809_at	1.35	2.54	AAD28599.1	3e-39
Inorganic pyrophosphatase [*A. thaliana*]	HV_CEa0012H06f_at	-1.09	2.56	NP_196527.1	6e-14
Peptidylprolyl isomerase [*A. thaliana*]	Contig14849_at	1.35	2.59	NP_567750.1	2e-38
Probable b-keto acyl reductase [*H. vulgare*]	Contig7485_at	-1.07	2.61	T06214	3e-34
Alpha-hordothionin [*H. vulgare*]	S0001100036G10F1_x_at	1.24	2.61	JA0087	7e-39
Putative peroxidase [*O. sativa*]	Contig7080_at	-1.04	2.62	AAG46142.1	7e-25
Expressed protein [*A. thaliana*]	Contig4700_at	1.17	2.63	NP_567209.1	2e-62
Formate dehydrogenase (FDH) [*H. vulgare*]	HA10H23u_x_at	1.42	2.64	Q9ZRI8	1e-11
Putative thionin [*H. vulgare*]	HVSMEb0010B05r2_x_at	-1.36	2.68	CAD48489.1	3e-26

Similar to Babesia aldo-keto reductase [*A. thaliana*]	HVSMEm0020M20r2_x_at	1.22	2.69	AAB70433.1	4e-05
Germin A [*H. vulgare*]	Contig3151_at	-1.26	2.7	AAG00425.1	e-118
Chalcone isomerase [*H. vulgare*]	Contig9047_at	1.76	2.7	AAM13449.1	8e-84
Chalcone isomerase [*H. vulgare*]	Contig9048_s_at	1.52	2.72	AAM13449.1	4e-14
P450 [T. aestivum]	Contig4271_at	1.2	2.73	BAB87820.1	6e-63
Putative quinone oxidoreductase [*O. sativa*]	HV_CEa0006E02r2_at	-1.15	2.74	AAK98702.1	1e-48
NBS-LRR-like protein [*H. vulgare*]	Contig21758_at	-1.93	2.76	AAL07811.1	1e-47
Putative protein [*A. thaliana*]	Contig12028_at	1.41	2.77	NP_197695.1	2e-55
Peptidyl-prolyl isomerase protein [*A. thaliana*]	Contig4537_at	1.18	2.85	NP_196816.1	4e-71
Probable phenylalanine ammonia-lyase [*H. vulgare*]	HVSMEm0015M15r2_s_at	1.41	2.87	T05968	3e-13
ABA-inducible protein PHV A1[*H. vulgare*]	HT11O04r_at	-1.34	2.93	P14928	1e-16
Similar to Babesia aldo-keto reductase [*A. thaliana*]	HVSMEm0020M20r2_s_at	1.17	3	AAB70433.1	4e-05
Glutaredoxin [*A. thaliana*]	EBpi03_SQ001_N23_at	-1.4	3.02	NP_197550.1	0.037
Disease resistance response protein-related [*A. thaliana*]	Contig10245_at	-1.56	3.04	NP_176113.1	9e-40
Putative proteinase inhibitor [*H. vulgare*]	Contig50_x_at	1.14	3.05	CAB71340.2	6e-27
Putative flavanone 3-hydroxylase [*O. sativa* (japonica)]	Contig11212_at	1.78	3.07	AAL58118.1	1e-72
Putative peroxidase [*O. sativa* (japonica)]	Contig11509_at	1.61	3.09	BAC10867.1	3e-73
Thionin precursor, leaf [*H. vulgare*]	Contig1568_x_at	1.04	3.11	S22515	2e-65
Thionin [*H. vulgare*]	Contig1576_at	1.1	3.18	AAB21531.1	4e-61
Putative Cf2/Cf5 disease resistance protein [*O. sativa* (japonica)]	EBpi01_SQ002_L03_at	1.96	3.18	BAB89966.1	8e-08
Thionin precursor, leaf [*H. vulgare*]	Contig1582_x_at	1.53	3.32	S22515	4e-69
Zinc finger, C3HC4 type [*C. elegans*]	HVSMEm0012P04r2_at	-1.1	3.35	NP_495976.1	0.014
Putative peroxidase [*O. sativa*]	Contig7082_at	-1.23	3.43	AAG46125.1	8e-46
Tobacco mosaic virus helicase domain-binding protein [*N. tabacum*]	HV_CEb0010F15r2_at	1.45	3.48	AAL25088.1	3e-41
Thionin Osthi1 [*O. sativa* (japonica)]	Contig7008_at	1.21	3.51	BAB93111.1	3e-09
Zinc finger A20 and AN1 domain-containing stress-associated protein 2 [*O. sativa* (japonica)]	Contig1785_at	-1.44	3.68	BAB68048.1	7e-34
Metallothionein [Porteresia coarctata]	Contig1383_at	-1.06	4	AAF68995.1	2e-14
Kalata B2 precursor [*Oldenlandia affinis*]	Contig9249_at	1.48	4.64	P58454	0.009
Thionin [*H. vulgare*]	Contig1579_s_at	1.09	4.97	AAB21531.1	2e-67
P450 [T. aestivum]	Contig15560_at	1.24	5.66	BAB87820.1	6e-58
Putative proteinase inhibitor [*H. vulgare*]	Contig34_s_at	1.52	5.83	CAB71340.2	1e-33
Subtilisin-chymotrypsin inhibitor 2 [*H. vulgare*]	Contig3383_at	-1.85	6.57	T06181	4e-20
Thionin precursor, leaf [*H. vulgare*]	Contig1580_x_at	1.67	7.75	S22515	6e-71
Thionin [*H. vulgare*]	Contig1570_s_at	1.99	10.99	AAB21531.1	1e-76
Thionin [*H. vulgare*]	HVSMEl0005O09r2_at	1.08	12.12	AAB21529.1	5e-22
Prohibitin [Z. mays]	Contig3129_at	-1.11	13.24	AAF68387.1	e-105
Ice recrystallisation inhibition protein [*L. perenne*]	Contig7221_at	-1.03	26.23	CAB87814.1	8e-33
Branched-chain alpha-keto acid decarboxylase [*A. thaliana*]	Contig1965_at	-1.06	2.41	NP_175947.1	7e-39
Putative proteinase inhibitor [*H. vulgare*]	EBro08_SQ005_A14_at	1.09	2.46	CAB71340.2	4e-33
**Transport**					
Na^{+}-dependent inorganic phosphate cotransporter [*A. thaliana*]	HVSMEn0024A10f_at	-1.03	2	NP_199250.1	5e-08
Secretory carrier membrane protein [*A. thaliana*]	HW06D22u_at	-1.09	2.02	NP_176320.1	8e-34
Phosphoribosylanthranilate transferase [*A. thaliana*]	Contig11654_at	-1.15	2.03	NP_173675.1	4e-88
Major facilitator superfamily antiporter [*O. sativa* (japonica)]	HVSMEm0015A09r2_x_at	-1.14	2.08	AAN33181.1	2e-29
Putative N-hydroxycinnamoyl/benzoyl transferase [*O. sativa* (indica)]	Contig6770_at	1.36	2.09	CAC09504.1	5e-37
Peptide transporter, putative [*A. thaliana*]	Contig12317_at	1.19	2.1	NP_177024.1	2e-49
H.cysteine S-methyltransferase-3 [*Z. mays*]	HVSMEl0014E16r2_at	-1.36	2.15	AAG22539.1	3e-16
Auxin-regulated protein [*A. thaliana*]	Contig17921_at	1.54	2.17	NP_568128.1	3e-66
OSJNBa0072F16.15 [*O. sativa* (japonica)]	HV05C10u_at	-1.07	2.17	CAD40991.1	1e-40
IDI-7 [Podospora anserina]	Contig25878_at	1.08	2.26	AAN41258.1	4e-39
Contains similarity to ATFP3 [*A. thaliana*]	Contig3057_s_at	1.65	2.26	BAB08818.1	2e-44
OSJNBa0072F16.15 [*O. sativa* (japonica)]	HV05C10u_s_at	1.64	2.28	CAD40991.1	1e-40
Nonspecific lipid-transfer protein [*Malus domestica*]	Contig12237_at	-1.89	2.31	Q9M5X7	2e-09
ATP sulfurylase, putative [*A. thaliana*]	Contig14398_s_at	1.5	2.33	AAM63185.1	3e-12
Lipid transfer protein 7a2b [*H. vulgare*]	Contig698_at	1.95	2.34	T05950	8e-55
Similar to transitin [*M.musculus*]	Contig24850_at	1.3	2.48	XP_142961.1	1e-07
Cellulose synthase-3 [*Z. mays*]	Contig4451_s_at	1.6	2.48	AAF89963.1	e-124
Putative glutathione synthetase [*Z. mays*]	Contig21604_at	-1.21	2.6	CAC83006.1	1e-42
N-acetylglucosaminyl transferase [*O. sativa*]	Contig19664_s_at	-1.1	2.64	CAD30022.1	5e-29
Major facilitator superfamily antiporter [*O. sativa* (japonica)]	Contig9634_at	1.46	2.65	AAN33182.1	4e-65

Putative transketolase [*O. sativa* (japonica)]	HVSMEa0011L19r2_x_at	1.17	2.71	BAB19388.1	3e-17
Mitochondrial carrier protein family[*A. thaliana*]	HVSMEl0014H01r2_at	1.1	2.8	NP_568060.1	1e-17
Uracil phosphoribosyltransferase-like protein[*A. thaliana*]	Contig19273_at	1.85	2.81	NP_190958.1	9e-26
Putative chloroplast chaperonin [*O. sativa*]	HVSMEa0004A18r2_at	1.73	2.83	AAL79700.1	9e-28
Putative vacuolar proton-ATPase subunit 1 [*O. sativa* (japonica)]	HS08H14u_at	-1.03	2.88	BAB92928.1	8e-14
RAB6, member RAS oncogene family [*M.musculus*]	Contig22053_at	-1.12	3.01	NP_077249.1	3e-77
Reticulon-like protein B2 [*A. thaliana*]	HS06C20u_at	1.33	3.01	NP_192861.1	1e-27
P-rich protein Nt-SubC29 [*N. tabacum*]	Contig3778_x_at	1.19	3.16	BAB16431.1	5e-14
Glu-tRNA(Gln) amidotransferase subunit C [*A. thaliana*]	Contig16053_at	1.79	3.4	AAG29097.1	3e-24
Maebl [P. yoelii yoelii]	HV09L12u_at	-1.4	3.45	EAA21236.1	0.07
Putative protein; protein id: At5g48660.1 [*A. thaliana*]	Contig8877_at	1.04	3.46	NP_199677.1	3e-06
Glu-tRNA(Gln) amidotransferase subunit C [*A. thaliana*]	HK01E08r_s_at	1.71	3.49	AAG29097.1	2e-04
Copper homeostasis factor [*A. thaliana*]	HVSMEf0019G16r2_at	1.01	3.62	NP_564870.1	4e-14
Putative microtubule-severing protein subunit [*O. sativa* (japonica)]	HVSMEm0008I05r2_at	-1.22	4.06	AAM92815.1	4e-04
Probable potassium channel protein [*H. vulgare*]	Y09748_at	-1.16	4.23	T05935	5e-37
Putative auxin-responsive protein IAA2 [*O. sativa*]	HVSMEb0011L05r2_at	-1.04	4.61	AAK98708.1	7e-09
Similar to A. thaliana chromosome [*O. sativa*]	Contig5588_at	-1.07	5.1	BAA92405.1	5e-19
Putative protein; protein id: At3g62660.1 [*A. thaliana*]	HVSMEc0020G05f_x_at	1.65	2.57	NP_191825.2	7e-15
**Transcription**					
U2 snRNP auxiliary factor, large subunit [*Nicotiana plumbaginifolia*]	HT09G06u_s_at	1.21	2	CAA77136.1	3e-19
Putative protein; protein id: At5g16470.1 [*A. thaliana*]	Contig4486_at	1.86	2.02	NP_197151.1	3e-34
Putative protein; protein id: At4g12060.1 [*A. thaliana*]	Contig8787_s_at	1.32	2.03	NP_567386.1	4e-06
Glycine-rich RNA-binding protein [*O. sativa* (japonica)]	Contig17116_at	1.26	2.06	T04346	4e-34
U2 snRNP auxiliary factor, large subunit [*N. plumbaginifolia*]	HT09G06u_at	-1.42	2.07	CAA77136.1	3e-19
Expressed protein; protein id: At1g27760.1 [*A. thaliana*]	Contig10309_s_at	1.25	2.11	NP_564294.1	7e-06
Putative protein; protein id: At4g39470.1 [*A. thaliana*]	Contig23874_at	1.26	2.11	NP_568061.1	4e-32
Polyadenylate-binding protein II (PAB2) [*A. thaliana*]	S0001100024A03F1_x_at	1.51	2.14	NP_196597.1	6e-11
Heat shock transcription factor HSF1 [*A. thaliana*]	Contig6968_at	1.49	2.16	NP_189095.1	4e-43
Histone H3[*T. aestivum*]	HX03P07u_at	1.15	2.16	P02300	1e-40
Vrga1 [Aegilops ventricosa]	HX08E06r_at	1.47	2.18	AAF19148.1	2e-28
Putative protein; protein id: At5g62580.1[*A. thaliana*]	Contig11745_at	1.27	2.19	NP_201064.2	5e-29
Sun protein (fmu protein), putative [*A. thaliana*]	EBma01_SQ005_J20_at	1.07	2.2	NP_187924.2	0.036
AT3g03380/T21P5_20 [*A. thaliana*]	Contig25436_at	-1.12	2.23	AAL84951.1	0.036
Expressed protein; protein id: At3g21200.1[*A. thaliana*]	HVSMEb0005M12r2_at	1.09	2.23	NP_566678.1	4e-21
(AC010793) F20B17.8 [*A. thaliana*]	HVSMEl0006J08r2_at	1.62	2.25	AAF68123.1	4e-08
ORF63 [*O. sativa* (japonica)]	HVSMEl0012A13f_at	1.49	2.26	NP_039438.1	2e-16
Glucose-6P/phosphate translocator precursor [*Mesembryanthemum crystallinum*]	HVSMEg0005J01r2_x_at	1.02	2.29	AAF86908.1	6e-23
Ubiquitin-conjugating enzyme E2-23 kDa [*T. aestivum*]	Contig3907_at	1.17	2.3	P16577	3e-80
Histone H4 [*T. aestivum*]	Contig30_at	1.02	2.31	HSWT41	8e-40
Expressed protein; protein id: At2g36885.1[*A. thaliana*]	Contig10158_at	1.58	2.32	NP_565853.1	3e-52
Putative transposon protein [*O. sativa*]	HVSMEi0006G21r2_at	1.2	2.37	AAK63883.1	3e-13
JUN-like bZIP transcription factor [*Emericella nidulans*]	HVSMEi0002D19r2_at	-1.02	2.38	AAM00250.1	4e-04
P0681F05.22 [*O. sativa* (japonica)]	Contig13711_at	1.55	2.41	BAC45150.1	3e-13
Chloroplast RNA-binding protein [*A. thaliana*]	Contig12973_at	1.7	2.42	NP_176208.1	3e-69
NBS-LRR-like protein [*H. vulgare*]	AF414176_at	-1.23	2.48	AAL07815.1	0
Tic62 protein [*Pisum sativum*]	rbasd23b03_s_at	1.13	2.56	CAC87810.2	0.004
Ubiquitin-conjugating enzyme E2-18 kDa [*A. thaliana*]	Contig4967_at	-1.07	2.59	P42743	4e-83
Ubiquitin-conjugating enzyme E2-17 kDa [*Solanum lycopersicum*]	Contig2231_at	1.27	2.61	P35135	5e-33
Putative 32.7 kDa jasmonate-induced protein [*H. vulgare*]	HVSMEm0015P16r2_at	1.18	2.65	T04375	5e-10
Putative calcium binding EF-hand protein [*H. vulgare*]	AJ250283_at	-1.18	2.68	CAB71337.1	e-117
Contains ESTs AU030866(E60360) [*O. sativa*]	EBem05_SQ004_C15_at	-1.46	2.68	BAB92518.1	2e-04
Polyadenylate-binding protein II, putative [*A. thaliana*]	S0001100024A03F1_at	1.19	2.7	NP_196597.1	6e-11
Putative histone H2A [*O. sativa* (japonica)]	HA12K13u_at	-1.15	2.72	AAN06860.1	8e-14
Putative protein [*A. thaliana*]	HVSMEf0019G08r2_s_at	-1.03	2.75	NP_200458.1	2e-12
Putative AP2 domain transcriptional regulator [*O. sativa* (japonica)]	Contig18390_at	1.93	2.77	AAM08622.1	9e-12
Cp31AHv protein [*H. vulgare*]	rbasd3a10_s_at	1.24	2.8	T05725	3e-46
Putative GTP-binding protein [*O. sativa* (japonica)]	Contig7575_at	1.41	2.88	AAK09228.1	e-106
Putative ribosomal RNA apurinic site specific lyase [*O. sativa* (japonica)]	Contig19093_at	-1.44	2.89	BAC16431.1	3e-07
Ycf65-like protein, chloroplast precursor [*H. vulgare*]	Contig6675_at	1.87	2.91	O48609	5e-62
Replication protein A2 [*O. sativa*]	Contig20672_x_at	-1.03	2.92	CAC03572.1	4e-04
Calcyclin binding protein-like [*O. sativa* (japonica)]	HU12O04u_at	1.19	3.12	BAB90152.1	2e-20
Peptide chain release factor 2, putative [*A. thaliana*]	HV05A02u_at	1.35	3.15	AAG50902.1	5e-06
Glutamate--tRNA ligase precursor [*H. vulgare*]	Contig11355_at	1.64	3.16	Q43768	e-124

Cullin [A. thaliana]	HVSMEb0008L04r2_x_at	1.19	3.26	BAB08502.1	1e-13
DNA binding protein [*H. vulgare*]	Contig9071_at	-1.2	3.53	CAA04440.1	e-115
Putative SCARECROW gene regulator-like [*O. sativa* (japonica)]	Contig9594_at	1.27	3.56	AAM08829.1	2e-74
Nascent polypeptide associated complex alpha chain [*N. tabacum*]	HVSMEf0015C15f_at	1.17	3.68	T03926	2e-04
Glutamyl-tRNA synthetase [*H. vulgare*]	Contig11354_at	1.01	4.34	Q43768	2e-60
Putative protein; protein id: At5g51180.1 [*A. thaliana*]	HW09E23u_at	1.24	4.65	NP_568754.1	1e-10
Mature anther-specific protein LAT61 [*L. esculentum*]	EBem09_SQ002_L08_x_at	1.38	7.83	AAK71662.1	0.026
Putative transposase protein [*O. sativa*]	HVSMEb0002O06r2_at	-1.15	16.17	AAK91322.1	3e-06
Cullin-like protein [*O. sativa* (japonica)]	HS01K16w_at	-1.15	2.67	BAB64732.1	1e-10
**Carbohydrate metabolism**					
Putative amylase [O. sativa (japonica)]	Contig8246_at	1.69	2	AAK27799.1	1e-93
Putative dermal glycoprotein precursor [*O. sativa* (japonica)]	Contig4632_s_at	1.18	2.01	BAB89707.1	9e-38
(Y14658) m12 [*H. vulgare*]	HS18K19u_s_at	1.34	2.01	CAA74988.1	5e-15
Putative allyl alcohol dehydrogenase [*O. sativa* (japonica)]	HVSMEg0006O20r2_at	1.38	2.06	BAB90185.1	4e-87
Putative carboxymethylenebutenolidase [*O. sativa* (japonica)]	Contig4769_at	1.19	2.07	BAB91862.1	e-100
Ribulose-1,5-bisphosphate carboxylase/oxygenase small subunit [*T. aestivum*]	Contig838_s_at	1.02	2.1	BAB19812.1	3e-94
Putative dTDP-glucose 4,6-dehydratase [*Pinus sylvestris*]	HVSMEm0018L03r2_at	-1.84	2.1	AAL74389.1	2e-12
Polygalacturonase [*L. esculentum*]	Contig21718_at	1.5	2.14	AAF61444.1	2e-26
Putative protein; protein id: At4g39970.1 [*A. thaliana*]	Contig23996_at	1.04	2.21	NP_568077.1	6e-49
Hypothetical protein alr3813 [*Anabaena sp.*]	Contig6656_at	1.53	2.21	NP_487853.1	7e-14
Cinnamoyl CoA reductase [*L. perenne*]	Contig6799_at	1.31	2.24	AAG09817.1	2e-92
Putative lectin [*H. vulgare*]	Contig11641_at	1.56	2.3	CAB40792.1	1e-91
Similar to NAD-dependent glyceraldehyde-3-phosphate dehydrogenase [*A. thaliana*]	Contig3720_at	-1.06	2.33	AAD34682.1	2e-46
Ribulose-1,5-bisphosphate carboxylase/oxygenase small subunit [*T. aestivum*]	Contig497_s_at	1.04	2.33	BAB19812.1	6e-60
UDP-glucuronic acid decarboxylase [*O. sativa* (japonica)]	rbags10p20_at	1.25	2.34	BAB84334.1	4e-27
Putative Glucan 1,3-beta-glucosidase precursor [*O. sativa* (japonica)]	HVSMEf0013J20f_at	-1.74	2.37	AAM08620.1	7e-25
Glycosyl hydrolase family 17 [*A. thaliana*]	Contig18116_at	1.25	2.44	NP_181494.1	1e-30
AT3g05350/T12H1_32 [*A. thaliana*]	Contig12679_at	1.28	2.47	AAL84973.1	3e-52
Putative cinnamoyl-CoA reductase [*O. sativa*]	Contig11163_at	1.38	2.5	AAL67601.1	1e-72
Cinnamic acid 4-hydroxylase [*Lithospermum erythrorhizon*]	Contig3442_at	-1	2.5	BAB71717.1	2e-97
Granule-bound starch synthase [*H. vulgare*]	S0001100126A06F1_x_at	-1.24	2.51	AAL77109.1	2e-27
AT3g48690/T8P19_200 [*A. thaliana*]	HVSMEi0007L20r2_x_at	1.11	2.54	AAL57633.1	1e-07
P-glycoprotein pgp1 [*A. thaliana*]	Contig18416_s_at	1.08	2.58	S21957	1e-16
ribulose-bisphosphate carboxylase [*T. aestivum*]	HVSMEm0020L23r2_x_at	1.23	2.58	RKWTS	1e-36
Putative lectin [*H. vulgare*]	AJ303111_s_at	1.65	2.62	CAC19668.1	3e-36
Glycosyl hydrolase family 3 [*A. thaliana*]	Contig21549_at	-1.17	2.65	NP_190288.1	8e-78
Putative 3-beta hydroxysteroid dehydrogenase/isomerase [*O. sativa*]	Contig6963_at	1.98	2.74	AAK73149.1	e-101
Cytosolic fructose-1,6-bisphosphatase [*M. acuminata*]	HU01I17w_at	1.7	2.79	AAD28755.1	1e-27
Soluble epoxide hydrolase [*Brassica napus*]	Contig5727_at	1.22	2.8	CAD30841.1	2e-65
Enolase [*O. sativa* (japonica)]	Contig1294_at	1.09	2.89	Q42971	e-126
Contains similarity to endo-1,3-1,4-beta-D-glucanase [*A. thaliana*]	HVSMEi0005H10r2_at	-1.04	2.91	BAB02775.1	4e-15
Ribulose bisphosphate carboxylase small chain[*T. aestivum*]	baak2o06_x_at	1.08	3.06	P05347	0.001
Ribulose bisphosphate carboxylase small chain [*T. aestivum*]	Contig594_x_at	1.12	3.08	P26667	5e-97
Cinnamoyl-CoA reductase [*L. perenne*]	Contig15809_at	1.61	3.2	AAL47182.1	3e-12
Putative beta-glucosidase [*O. sativa* (japonica)]	rbah54a21_at	-1.05	3.22	BAB67933.1	2e-29
Glucan endo-1,3-beta-glucosidase GIII [*H. vulgare*]	Contig1636_at	-1.14	3.28	Q02126	e-164
Serine/threonine protein phosphatase [*A. thaliana*]	Contig6535_at	1.05	3.31	Q9XF94	1e-52
Ribulose-1,5-bisphosphate carboxylase/oxygenase [*T. aestivum*]	HVSMEa0011D13r2_at	1.38	3.31	BAB19814.1	9e-17
Similar to alcohol dehydrogenase [*O. sativa* (japonica)]	Contig4526_at	1.39	3.37	BAC10091.1	5e-74
Cytosolic aldehyde dehydrogenase RF2C [*Z. mays*]	Contig6381_at	1.54	3.59	AAL99608.1	4e-97
UDP-glucose dehydrogenase [*P. tremula x P. tremuloides*]	HVSMEl0011E20r2_at	1.36	3.62	AAF04455.1	3e-09
Ribulose-bisphosphate carboxylase [*T. aestivum*]	Contig589_x_at	-1.05	3.7	RKWTS	3e-97
T1N15.3 [*A. thaliana*]	HVSMEa0012J22r2_at	-1.09	4.02	AAF79717.1	1e-22
Putative mannan endo-1,4-beta-mannosidase [*O. sativa* (japonica)]	HY04F23u_at	1.18	4.61	BAB91747.1	0.011
UDP-glucose 6-dehydrogenase [*G. max*]	rbags10b05_at	-1.98	5.47	Q96558	1e-46
Tonneau 1 [*O. sativa*]	Contig7890_at	-1.72	5.74	AAG35782.1	1e-52
Glyoxalase I [*O. sativa* (japonica)]	HW08F16V_x_at	-1.04	8.49	BAA36759.1	5e-15
Ribulose-1,5-bisphosphate carboxylase/oxygenase [*T. aestivum*]	Contig842_x_at	1.42	9.21	BAB19811.1	9e-97
Ribulose-bisphosphate carboxylase [*T. aestivum*]	Contig997_x_at	1.5	9.39	RKWTS	4e-88
Ribulose-1,5-bisphosphate carboxylase/oxygenase [*H. vulgare*]	Contig1004_x_at	-1.11	21.02	BAA35162.1	7e-80
**Cell growth, division**					
Trehalose-6-phosphate synthase, putative [*A. thaliana*]	Contig10696_at	-1.26	2.26	NP_177979.1	2e-19
Cyclin H-1 [*O. sativa*]	Contig18413_at	-1.47	4.89	BAB11694.1	7e-04

**Fat metabolism**					
Cytosolic acetyl-CoA carboxylase [*T. aestivum*]	Contig11326_at	1.31	2.11	A57710	1e-80
3-oxoacyl-[acyl-carrier-protein] synthase I, [*H. vulgare*]	Contig18583_at	1.56	2.26	P23902	2e-94
Putative fatty acid hydroxylase [*O. sativa* (japonica)]	Contig6314_at	1.1	2.35	AAK09233.1	2e-25
Putative APG protein [*A. thaliana*]	Contig12400_at	-1.04	2.42	NP_188258.1	3e-23
Lipase-like protein [*O. sativa* (japonica)]	Contig20235_s_at	-1.19	2.44	BAB89205.1	2e-19
Cyclopropane-fatty-acyl-phospholipid synthase, putative [*A. thaliana*]	Contig12308_at	1.34	2.52	NP_188993.1	1e-82
Putative stearoyl-acyl carrier protein desaturase [*O. sativa* (japonica)]	HS05L19u_at	1.47	2.54	BAC06230.1	1e-07
Putative lipoxygenase [*O. sativa* (japonica)]	Contig12574_at	1.64	2.69	AAL83618.1	1e-43
Cytosolic triosephosphate isomerase [*H. vulgare*]	Contig1629_x_at	1.01	3.35	P34937	2e-13
Nitrogen metabolism					
Probable hordein B1 [*H. vulgare*]	HB01O23r_x_at	-1.16	2.17	T04473	2e-19
Tryptophan synthase [*Neurospora crassa*]	HVSMEm0018C06r2_at	1.08	2.17	P13228	6e-67
Putative peptidyl-prolycis-trans isomerase protein [*O. sativa* (japonica)]	Contig16181_at	1.12	2.21	BAC22549.1	4e-53
Putative peptidyl-prolyl cis-trans isomerase[*O. sativa* (japonica)]	Contig9747_at	1.54	2.23	BAB39968.1	1e-79
Putative leucine zipper protein [*O. sativa* (japonica)]	Contig23563_at	-1.19	2.25	BAB86177.1	5e-53
Hordein [*H. vulgare*]	HB26H24r_x_at	1.01	2.36	AAA32967.1	3e-14
Jasmonate induced protein [*H. vulgare*]	Contig2899_at	1.19	2.42	S58215	6e-93
Putative gamma glutamyl hydrolase [*A thaliana*]	HVSMEk0023O04r2_at	-1.6	2.42	AAL49804.1	2e-22
Putative 3-isopropylmalate dehydrogenase[*A. thaliana*]	Contig5555_at	1.28	2.05	NP_178171.1	e-105
Isoleucine-tRNA ligase [*A. thaliana*]	Contig9189_at	-1.05	2.07	NP_192770.1	2e-69
Putative protein; protein id: At5g20520.1 [*A. thaliana*]	HM12J12r_at	-1.28	2.07	NP_568395.1	3e-07
Basic leucine zipper protein [*Z. mays*]	Contig16432_at	-1.04	2.1	T01415	1e-65
Seed maturation protein, putative [*A. thaliana*]	HS05M19u_at	-1.43	2.15	AAM64658.1	9e-26
Putative aspartate transaminase [*O. sativa* (japonica)]	HVSMEf0021O16r2_x_at	-1.12	2.16	BAB63467.1	9e-22
Putative protein; protein id: At5g33370.1 [*A. thaliana*]	HVSMEn0013D02r2_x_at	-1.14	2.26	NP_198322.1	2e-09
Putative aspartate transaminase [*O. sativa* (japonica)]	Contig4244_at	1.89	2.35	BAB63467.1	6e-93
(AC007396) T4O12.25 [*A. thaliana*]	Contig10834_at	1.63	2.64	AAF26765.1	3e-78
Putative aspartate transaminase [*O. sativa* (japonica)]	HVSMEf0021O16r2_at	1	2.89	BAB63467.1	9e-22
GMP synthase; protein id: At1g63660.1 [*A. thaliana*]	Contig8886_at	1.09	3.02	NP_176553.1	9e-68
Gamma 3 hordein [*H. vulgare*]	EBed07_SQ003_I06_at	-1.28	3.78	T05718	2e-04
Hydroxyproline-rich glycoprotein [*O. sativa*]	Contig553_s_at	1.71	3.81	S20500	6e-05
Leucyl aminopeptidase [*P. crispum*]	HV10J10u_at	1.86	12.32	T14912	1e-08
**Photosynthesis**					
Chlorophyll a/b binding protein [*Solanum tuberosum*]	Contig981_s_at	1.24	2.33	AAA80589.1	3e-95
Chlorophyll a/b binding protein [*S. tuberosum*]	Contig926_x_at	1.17	2.34	AAA80589.1	e-116
Protochlorophyllide reductase B [*H. vulgare*]	Contig2766_s_at	1.16	2.08	Q4285	2e-54
Protoporphyrin IX magnesium chelatase [*H. vulgare*]	Contig5341_at	1.36	2.09	S64722	e-167
Uroporphyrinogen decarboxylase [*H. vulgare*]	Contig6329_at	1.84	2.28	Q42855	e-138
Thylakoid lumen 15.0-kDa protein [*A. thaliana*]	Contig9582_at	1.29	2.28	NP_568781.1	5e-72
Mg-protoporphyrin IX [*H. vulgare*]	Contig10699_at	1.61	2.3	CAB58179.1	e-151
Putative phytochrome-associated protein [*O. sativa* (japonica)]	HV_CEb0024B09r2_s_at	-1.34	2.43	BAB91924.1	1e-19
Chlorophyll a/b-binding protein 1A precursor [*S. lycopersicum*]	baak26h09_x_at	1.35	2.53	A24039	2e-18
Putative phytochrome-associated protein [*O. sativa* (japonica)]	HV_CEb0024B09r2_at	-1.76	2.55	BAB91924.1	1e-19
Putative uroporphyrinogen decarboxylase [*O. sativa* (japonica)]	Contig8595_at	1.88	2.58	BAB21078.1	e-113
Chloroplast Cpn21 protein; protein id: At5g20720.1 [*A. thaliana*]	Contig10790_at	1.92	2.59	NP_197572.1	1e-78
Chlorophyll a/b-binding protein WCAB precursor [*T. aestivum*]	Contig628_x_at	1.01	2.74	AAB18209.1	e-104
Chlorophyll a/b-binding protein WCAB precursor [*T. aestivum*]	Contig347_s_at	1.07	2.91	AAB18209.1	6e-94
Mg-chelatase subunit XANTHA-F [*H. vulgare*]	Contig2985_s_at	1.4	2.99	AAK72401.1	e-139
Thylakoid lumen pentapeptide repeat family protein[*A. thaliana*]	HVSMEa0012N07r2_at	1.33	3.06	NP_566030.1	1e-51
Lil3 protein [*A. thaliana*]	Contig2314_at	1.97	3.08	AAM63936.1	2e-49
Chlorophyll a/b-binding protein WCAB precursor [*T. aestivum*]	Contig425_at	1.15	3.52	AAB18209.1	e-113
Chlorophyll a/b-binding protein WCAB precursor [*T. aestivum*]	Contig828_s_at	1.21	3.7	AAB18209.1	e-138
Chlorophyll A-B binding protein 3A [*S. lycopersicum*]	Contig960_s_at	1.84	4.11	P14276	6e-19
Chlorophyll a/b-binding protein WCAB precursor [*T. aestivum*]	Contig841_x_at	1.1	4.79	AAB18209.1	e-120
Chlorophyll a/b-binding protein WCAB precursor [*T. aestivum*]	Contig1012_s_at	1.18	5.02	AAB18209.1	4e-79
Chlorophyll A-B binding protein 3C [*S. lycopersicum*]	Contig25177_at	1.9	5.18	P07369	6e-74
Chlorophyll a/b-binding protein WCAB precursor [*T. aestivum*]	Contig418_at	1.89	8.28	AAB18209.1	e-128
Chlorophyll a/b-binding protein WCAB precursor [*T. aestivum*]	Contig422_at	1.38	12.09	AAB18209.1	e-132
**Protein synthesis**					
Ribosomal protein S5 [*S. oleracea*]	Contig4439_at	1.71	2.02	CAA63650.1	2e-55
Ribosomal protein L35A [*Z. mays*]	Contig2450_at	1.01	2.05	AAL59231.1	9e-54
Putative CER1 [*O. sativa*]	HB08F22r_x_at	1.1	2.05	AAG21908.1	2e-13

40S probable ribosomal protein S15 [*O. sativa*]	HV_CEa0012L06r2_x_at	-1.09	2.07	P31674	9e-14
Ribosomal protein L3 precursor [*N. tabacum*]	HA28J12r_s_at	1.68	2.16	T01736	0.013
50S ribosomal protein L5 [*O. sativa*]	Contig5776_s_at	1.62	2.17	AAC64970.1	3e-42
Plastid ribosomal protein CL15 [*A. thaliana*]	Contig5680_s_at	1.61	2.2	CAA77592.1	6e-53
Putative uncharacterized protein At5g05270 [*A. thaliana*]	Contig6242_at	1.35	2.22	NP_568154.1	2e-46
Putative lycopene epsilon-cyclase [O. sativa (japonica)]	Contig14290_at	1.29	2.23	BAC05562.1	e-118
Chalcone synthase [*S. cereale*]	Contig7358_at	1.12	2.27	P53414	6e-92
Putative monoterpene synthase [*O. sativa*]	Contig11021_at	1.06	2.28	AAL31055.1	1e-83
Small nuclear ribonucleoprotein at 69D[*D. melanogaster*]	Contig24581_at	-1.19	2.33	NP_524774.1	8e-36
Plastid-specific ribosomal protein [*Deschampsia antarctica*]	HVSMEa0002L16r2_at	1.29	2.34	AAM11915.1	4e-31
Putative chloroplast 50S ribosomal protein[*A. thaliana*]	Contig5573_at	1.6	2.36	NP_172011.1	7e-82
50S ribosomal protein L27[*O. sativa*]	Contig8437_at	1.56	2.36	O65037	6e-75
9S ribosomal protein [*Z. mays*]	Contig10093_s_at	1.68	2.38	AAK16543.1	2e-64
50S ribosomal protein L35 precursor [*S. oleracea*]	Contig9274_s_at	1.3	2.42	P23326	1e-28
Guanine nucleotide-binding protein subunit beta-like protein [*O. sativa*]	HVSMEg0007I08f_s_at	1.13	2.42	P49027	9e-08
Putative plastid ribosomal protein CL9 [*T. aestivum*]	Contig5492_at	1.52	2.43	AAM92711.1	4e-89
Putative plastid ribosomal protein L19 precursor [*O. sativa*]	Contig2941_at	1.7	2.49	CAC39039.1	4e-60
Ribosomal protein L17-like protein [*A. thaliana*]	Contig10356_at	1.81	2.56	AAM63452.1	4e-57
Plastid-specific ribosomal protein [*D. antarctica*]	HVSMEa0002L16r2_x_at	-1.81	2.56	AAM11915.1	4e-31
Ribosomal protein precursor [*A. thaliana*]	Contig5526_s_at	1.5	2.58	NP_568299.1	9e-46
Putative ribosomal protein L28 [*O. sativa*]	Contig4380_s_at	1.41	2.6	AAG03094.1	7e-36
Ribosome recycling factor [*S. oleracea*]	Contig5004_at	1.28	2.6	P82231	2e-71
Plastid-specific ribosomal protein 6 precursor [*S. oleracea*]	Contig5659_at	1.62	2.61	AAF64189.1	5e-20
Longevity assurance protein, putative [*A. thaliana*]	Contig13341_at	1.06	2.65	NP_172815.1	3e-38
Putative ribosomal protein L28 [*O. sativa*]	Contig4380_at	1.4	2.65	AAG03094.1	7e-36
Ribosomal protein L3 precursor [*N. tabacum*]	Contig5240_at	1.81	2.69	T01736	1e-93
Plastid ribosomal protein S6, putative [*A. thaliana*]	Contig8956_at	1.58	2.74	NP_176632.1	2e-44
60S ribosomal protein ML16 [*M. crystallinum*]	HV10E15u_at	-1	2.74	P34091	2e-08
Ribosomal protein L17-like protein [*A. thaliana*]	rbags18k24_s_at	1.83	2.85	AAM63452.1	2e-15
Plastid ribosomal protein CL15 [*A. thaliana*]	Contig5680_at	1.67	2.87	CAA77592.1	6e-53
Putative 50S ribosomal protein L34 [*O. sativa* (japonica)]	Contig5102_s_at	1.62	2.94	BAB92266.1	7e-34
50S ribosomal protein L35 precursor [*S. oleracea*]	Contig9274_at	1.89	2.94	P23326	1e-28
30S ribosomal protein S31 [*A. thaliana*]	Contig5708_at	1.66	2.96	NP_181349.1	8e-10
Naringenin-chalcone synthase [*H. vulgare*]	Contig7356_at	1.7	3.22	P26018	1e-88
Ribosomal protein L17-like protein [*A. thaliana*]	rbags18k24_x_at	1.71	3.29	AAM63452.1	2e-15
Putative elongation factor 1 beta [*H. vulgare*]	HY09G23u_x_at	-1.3	3.61	CAB90214.1	9e-17
Putative ribosomal protein L13 [*O. sativa* (japonica)]	Contig6936_at	1.97	3.64	BAB56046.1	e-105
Putative leucyl-tRNA synthetase [*A. thaliana*]	HVSMEa0013G22r2_at	-1.56	10.81	NP_172433.1	0.073
**Signal transduction**					
Putative protein kinase APK1A [*O. sativa* (japonica)]	HVSMEf0011I21r2_s_at	1.26	2.01	BAB55494.1	4e-26
Similar to serine/threonine kinases [*A. thaliana*]	Contig4086_at	1.06	2.07	AAF02840.1	4e-08
Putative protein kinase Xa21 [*O. sativa* (japonica)]	rbaal9i05_at	-1.73	2.07	BAC10827.]	4e-55
Putative wall-associated kinase 1 [*O. sativa* (japonica)]	Contig14031_at	-1.37	2.09	BAB40022.1	1e-81
T4O12.25 [*A. thaliana*]	Contig10835_at	1.41	2.14	AAF26765.1	1e-31
Vacuolar targeting receptor bp-80 [*T. aestivum*]	HVSMEa0002E06r2_at	1.06	2.14	AAF80450.1	3e-32
Putative protein kinase APK1B [*O. sativa* (japonica)]	Contig20776_at	1.08	2.18	BAB78635.1	2e-04
Shk1 kinase binding protein 15 [*S. pombe*]	HVSMEk0004A14r2_at	1.08	2.3	AAK51600.1	0.038
Putative receptor-type protein kinase LRK1 [*O. sativa*]	Contig13749_x_at	-1.06	2.35	BAC06925.1	2e-45
Expressed protein; protein id: At4g01150.1 [*A. thaliana*]	Contig13457_s_at	1.25	2.54	NP_567210.1	8e-38
OsCDPK7 [*O. sativa* (japonica)]	baal21h16_at	-1.37	3.19	BAB16888.1	1e-32
Phosphoribosyl pyrophosphate synthetase [*Saccharum hybrid*]	Contig5085_at	-1	3.34	AAN04491.1	8e-31
Nucleoside diphosphate kinase II [*S. oleracea*]	Contig7963_at	1.44	4.54	Q01402	2e-64
**Unknown classified**					
Expressed protein; protein id: At1g16080.1 [*A. thaliana*]	Contig13409_at	1.34	2	NP_563991.1	7e-35
Hypothetical protein; protein id: At2g32180.1 [*A. thaliana*]	Contig15715_at	1.82	2.01	NP_180777.1	3e-30
Unknown protein; protein id: At1g15290.1 [*A. thaliana*]	Contig11912_at	1.73	2.02	NP_172981.1	2e-65
Unknown protein [*O. sativa* (japonica)]	Contig20072_at	-1.28	2.02	AAM15787.1	9e-08
Hypothetical protein [*O. sativa* (japonica)]	Contig21194_s_at	-1.36	2.03	AAM51829.1	6e-47
Putative protein; protein id: At4g25370.1[*A. thaliana*]	Contig8788_at	1.47	2.04	NP_567718.1	6e-36
Putative protein; protein id: At5g41130.1 [*A. thaliana*]	HVSMEn0024J24r2_at	-1.45	2.04	NP_198929.1	5e-23
Unknown protein [*O. sativa* (japonica)]	Contig8064_at	1.3	2.06	BAB44030.1	2e-58
Hypothetical protein [*A. thaliana*]	Contig12503_s_at	1.01	2.08	T1346]	6e-41
Unknown protein [*O. sativa* (japonica)]	Contig18851_at	-1.13	2.08	BAC15986.a	2e-27

Unknown protein [*O. sativa*]	HK06N02r_at	-1.15	2.08	AAK82443.1	1e-07
Unknown protein [*O. sativa* (japonica)]	HVSMEa0001M23r2_at	1.03	2.09	BAB63527.1	2e-17
ESTs gb|T41688, gb|AI992698 [*A. thaliana*]	Contig12689_at	-1.04	2.1	AAF18514.1	9e-12
Putative protein; protein id: At5g05960.1 [*A. thaliana*]	Contig5474_at	1.76	2.11	NP_568160.1	5e-21
Arabinogalactan-protein [*A. thaliana*]	Contig6537_at	1.12	2.12	NP_566070.1	7e-10
Similar to unknown protein [*A. thaliana*]	Contig8508_at	1.91	2.12	NP_200633.1	3e-51
OSJNBb0021I10.13 [*O. sativa* (japonica)]	Contig12668_s_at	1.16	2.14	CAD39378.1	4e-37
Similar to nodulin [*O. sativa* (japonica)]	Contig1402_at	-1.35	2.14	BAC20892.1	1e-70
Unknown protein [*O. sativa* (japonica)]	Contig5712_at	1.04	2.15	BAB62639.1	1e-88
Hypothetical protein [*P. yoelii yoelii*]	HS08I03u_x_at	-1.31	2.15	EAA16547.1	2e-07
Unknown protein [*O. sativa* (japonica)]	Contig20713_at	1.92	2.16	BAB92723.1	7e-38
Expressed protein; protein id: At4g34090.1[*A. thaliana*]	Contig17805_at	1.48	2.17	NP_567951.1	5e-54
Similar to Nipped-B gene product [*O. sativa* (japonica)]	HVSMEg0017N16r2_at	-1.07	2.17	BAC15836.1	1e-80
Hypothetical protein [*O. sativa*]	Contig9589_at	1.93	2.2	AAK52167.1	6e-38
Putative protein [*A. thaliana*]	Contig24302_at	1.96	2.21	NP_193645.1	1e-23
Unknown protein [*O. sativa*]	HA11F06u_at	-1.11	2.21	AAL25173.1	0.018
Hypothetical protein [*O. sativa* (japonica)]	Contig9763_s_at	1.73	2.22	BAC19990.1	2e-42
Putative protein; protein id: At5g47060.1 [*A. thaliana*]	Contig7873_at	-1.09	2.23	NP_199517.1	7e-09
Expressed protein; protein id: At1g16080.1[*A. thaliana*]	HVSMEa0005L24r2_s_at	1.35	2.24	NP_563991.1	2e-10
LP01629p [D. melanogaster]	Contig5481_at	1.02	2.25	NP_651785.1	4e-07
Hypothetical protein [*O. sativa*]	Contig11363_at	1.27	2.26	AAK16173.1	3e-87
P0697C12.8 [*O. sativa* (japonica)]	rbasd11m11_at	-1.1	2.37	BAB64674.1	0.6
Hypothetical protein [*O. sativa* (japonica)]	Contig9951_s_at	1.39	2.38	BAB92407.1	9e-76
ESTs AU070372(S13446) [*A. thaliana*]	Contig4243_at	1.76	2.4	BAA82377.1	7e-74
Hypothetical protein; protein id: At2g03420.1 [*A. thaliana*]	Contig16125_at	1.95	2.42	NP_565301.1	5e-43
Hypothetical protein [*O. sativa* (japonica)]	Contig9952_at	1.35	2.42	BAB92407.1	7e-74
RIKEN cDNA 2210010N10 [*M.musculus*]	HD07I22r_at	-1.09	2.43	NP_082460.1	5e-08
Putative protein; protein id: At4g09620.1 [*A. thaliana*]	Contig8932_at	-1.37	2.52	NP_192700.1	6e-24
AgCP8235 [A. gambiae str. PEST]	Contig2024_at	1.05	2.58	EAA13897.1	0.006
Putative coatomer protein complex [*A. thaliana*]	HF09C04r_at	-1.28	2.62	NP_178116.1	0.011
Hypothetical protein [*H. vulgare*]	rbags25i16_at	1.8	2.67	S49173	6e-06
Hypothetical protein [*O. sativa* (japonica)]	HVSMEb0015P10r2_at	1.87	2.69	BAB39880.1	6e-11
Hypothetical protein T6H20.190 [*A. thaliana*]	Contig3396_s_at	1.43	2.7	T12970	3e-11
Similar to nodulin [*O. sativa* (japonica)]	Contig1404_x_at	-1.13	2.74	BAC20892.1	1e-83
(AF427791) BPM [*H. vulgare*]	Contig10288_at	1.02	2.76	AAM22812.1	e-113
Hypothetical protein [*O. sativa* (japonica)]	Contig11927_at	-1.42	2.79	BAB92864.1	9e-11
Hypothetical protein [*Thermobifida fusca*]	HT02L01r_at	-1.04	2.79	ZP_00059514.	0.061
Putative protein; protein id: At5g56480.1 [*A. thaliana*]	Contig2700_x_at	-1.31	2.84	NP_200459.1	6e-04
P0529E05.24 [*O. sativa* (japonica)]	Contig9863_at	1.23	2.86	BAB84408.1	5e-79
Hypothetical protein [*O. sativa*]	Contig24253_at	1.53	2.88	AAM08870.1	6e-21
Unknown protein; protein id: At2g33180.1 [*A. thaliana*]	Contig25351_at	1.62	2.95	NP_180876.1	8e-33
Expressed protein; protein id: At1g27760.1[*A. thaliana*]	Contig10309_at	1.16	2.96	NP_564294.1	7e-06
Expressed protein; protein id: At5g49940.1[*A. thaliana*]	Contig9435_at	-1.14	2.97	NP_568715.1	6e-42
Hypothetical protein [*Nostoc sp. PCC 7120*]	Contig6062_at	-1.01	3.1	NP_487053.1	1e-66
B1144G04.20 [*O. sativa* (japonica)]	Contig4215_at	1.67	3.23	BAB90029.1	9e-62
Hypothetical protein [*O. sativa* (japonica)]	Contig9057_at	-1.09	3.24	BAB86120.1	7e-66
ESTs D47168(S12332) [*Prunus armeniaca*]	Contig4273_at	-1.66	3.48	BAA95828.1	6e-52
Unknown protein [*O. sativa* (japonica)]	Contig10403_at	-1.66	3.54	AAM93676.1	2e-71
Contains ESTs AU172828(E31314) [*O. sativa* (japonica)]	Contig1404_at	-1.25	3.61	BAC20892.1	1e-83
Putative protein; protein id: At5g03150.1 [*A. thaliana*]	HS15C17r_at	-1.29	3.66	NP_195935.1	8e-04
Expressed protein; protein id: At4g29060.1 [*A. thaliana*]	Contig9936_at	1.76	3.95	NP_567820.1	2e-20
Hypothetical protein [*O. sativa* (japonica)]	EBan01_SQ005_B22_s_at	-1.34	4.01	BAA92905.1	4e-06
Unknown protein [*A. thaliana*]	Contig4011_at	1.64	5.48	AAM97054.1	6e-97
Unknown protein [*O. sativa* (japonica)]	HT09B09u_at	-1.61	7.64	AAG13433.1	9e-20
Putative protein; protein id: At5g27210.1[*A. thaliana*]	HU08E09u_at	1.23	11.78	NP_198073.1	4e-07
**None**					
none	EBpi03_SQ002_D12_at	-1.38	2	none	none
none	HA10M21u_at	1.08	2	none	none
none	HD12P17r_at	1.61	2	none	none
none	rbaal3o13_x_at	-1.2	2	none	none
none	rbags23o05_x_at	-1.03	2	none	none
none	Contig18687_at	-1.44	2.01	none	none
none	Contig6390_at	1	2.01	none	none

none	EBpi07_SQ002_J15_at	-1.33	2.01	none	none
none	HV_CEa0007J13r2_s_at	1.18	2.01	none	none
none	HVSMEm0017H02r2_at	-1.3	2.01	none	none
none	HVSMEn0022B10r2_s_at	1.32	2.01	none	none
none	HVSMEl0014J21r2_at	-1.24	2.02	none	none
none	HVSMEn0025O11r2_at	-1.44	2.02	none	none
none	EBro08_SQ012_P04_at	1.12	2.03	none	none
none	HU05P18u_x_at	-1.76	2.03	none	none
none	HVSMEf0009N22f_at	-1.18	2.03	none	none
none	HVSMEi0003I03r2_x_at	-1.14	2.03	none	none
none	HVSMEm0006E09r2_at	-1.03	2.03	none	none
none	HVSMEn0005O13f_x_at	-1.11	2.03	none	none
none	EBem10_SQ003_N11_at	1.09	2.04	none	none
none	HB10F07r_at	1.11	2.04	none	none
none	HM10P11r_s_at	1.27	2.04	none	none
none	Contig22151_at	1.01	2.05	none	none
none	Contig5302_at	1.5	2.05	none	none
none	Contig5303_at	1.23	2.05	none	none
none	EBpi01_SQ002_G19_at	1.22	2.05	none	none
none	HU05P18u_at	1.53	2.05	none	none
none	HZ45N21r_at	-1.13	2.05	none	none
none	Contig25410_at	1.05	2.06	none	none
none	HT11B22u_x_at	-1.01	2.06	none	none
none	HW01I01u_at	-1.06	2.06	none	none
none	HS18I18u_at	1.14	2.07	none	none
none	HVSMEn0017M01r2_at	1	2.07	none	none
none	Mla1_orf_3pri12_at	-1.41	2.07	none	none
none	HVSMEf0015D09r2_at	1.17	2.08	none	none
none	HVSMEi0003L18r2_at	-1.06	2.09	none	none
none	HVSMEm0005J23r2_at	1.19	2.09	none	none
none	Contig20805_at	1.62	2.1	none	none
none	HV11O23u_x_at	1.06	2.1	none	none
none	HVSMEi0003G04r2_s_at	-1.06	2.1	none	none
none	rbags19e09_at	-1.08	2.1	none	none
none	HV_CEa0009K20r2_x_at	1.75	2.11	none	none
none	HW03M21u_at	1.07	2.11	none	none
none	Contig11920_s_at	-1.31	2.12	none	none
none	HK03H11r_x_at	1.97	2.12	none	none
none	HVSMEf0010B06f2_x_at	1.13	2.12	none	none
none	HVSMEm0014C09r2_at	1.17	2.12	none	none
none	Contig15904_at	-1.59	2.13	none	none
none	Contig23196_at	1.15	2.13	none	none
none	EBem10_SQ001_I05_at	-1.15	2.13	none	none
none	HP01L10T_at	-1.07	2.13	none	none
none	HT07J02u_at	-1.12	2.13	none	none
none	Contig17816_s_at	-1.24	2.14	none	none
none	EBpi01_SQ004_E15_at	-1.26	2.14	none	none
none	Contig21381_at	1.13	2.15	none	none
none	Contig23137_at	-1.06	2.15	none	none
none	Contig23356_at	1.07	2.15	none	none
none	HV_CEa0015F23r2_x_at	-1.07	2.15	none	none
none	HM13O19r_at	1.16	2.16	none	none
none	HV_CEa0010K11f_x_at	1.16	2.16	none	none
none	HV_CEa0012D17r2_at	1.08	2.16	none	none
none	HU11H01u_at	-1.52	2.17	none	none
none	baak1h04_at	1.96	2.18	none	none
none	Contig11309_at	-1.23	2.18	none	none
none	HM06O03r_at	-1.27	2.18	none	none
none	rbasd24o18_s_at	1.06	2.19	none	none
none	Contig11540_at	1.35	2.2	none	none
none	HW07G01u_x_at	-1.43	2.2	none	none
none	HY08M06u_at	1.9	2.2	none	none
none	HF03J18r_at	1.09	2.21	none	none

none	HVSMEm0021D12f_s_at	-1	2.21	none	none
none	S0000200065D09F1_at	-1.03	2.21	none	none
none	Contig26575_at	-1.04	2.22	none	none
none	HW02P03u_x_at	-1.14	2.23	none	none
none	EBma03_SQ003_L14_s_at	1.03	2.24	none	none
none	HA04h14r_at	-1.02	2.24	none	none
none	HO09D16S_at	1.02	2.24	none	none
none	HV_CEb0011C06r2_at	-1.05	2.24	none	none
none	HS04L05u_x_at	-1.18	2.25	none	none
none	HU08L02u_at	1.17	2.25	none	none
none	HVSMEi0016B10r2_at	1.16	2.25	none	none
none	HVSMEm0023N10r2_at	1.1	2.25	none	none
none	Contig11217_at	-1.02	2.26	none	none
none	HW02E09u_s_at	1.16	2.27	none	none
none	Contig24402_at	-1.11	2.29	none	none
none	HV02E02u_at	-1.15	2.29	none	none
none	Contig16119_at	-1.21	2.3	none	none
none	Contig17920_at	1.1	2.3	none	none
none	Contig25328_at	-1.36	2.3	none	none
none	HVSMEb0007K08r2_x_at	-1.11	2.3	none	none
none	HW03F06u_at	-1.02	2.3	none	none
none	Contig3859_s_at	-1.28	2.31	none	none
none	HVSMEk0006K21r2_x_at	-1.42	2.31	none	none
none	Contig19752_at	1.09	2.32	none	none
none	HS05F15r_x_at	1.01	2.32	none	none
none	HV06H13u_x_at	1.34	2.32	none	none
none	Contig24133_at	-1.19	2.33	none	none
none	Contig25409_at	-1.02	2.33	none	none
none	Contig4552_at	-1.17	2.34	none	none
none	HVSMEl0006F02r2_x_at	-1.18	2.34	none	none
none	HU13L08u_at	-1.2	2.35	none	none
none	HVSMEg0012F06f2_at	-1.16	2.35	none	none
none	rbah41a21_x_at	-1.38	2.35	none	none
none	Contig17546_s_at	1.04	2.36	none	none
none	HU07L18r_x_at	1.11	2.37	none	none
none	HW03B09u_x_at	-1.24	2.37	none	none
none	HV10G07u_x_at	-1.33	2.38	none	none
none	HVSMEb0014F17r2_at	1.25	2.39	none	none
none	HVSMEm0007D16r2_at	1.15	2.39	none	none
none	Contig327_s_at	-1.07	2.4	none	none
none	rbags1o21_at	-1.58	2.4	none	none
none	Contig17988_at	1.27	2.41	none	none
none	HA12H06u_x_at	-1.2	2.41	none	none
none	HT08N20u_at	1.05	2.41	none	none
none	HVSMEk0007B15r2_s_at	-1.41	2.41	none	none
none	HV14K06u_x_at	1.12	2.42	none	none
none	HVSMEh0089C05r2_x_at	-1.22	2.44	none	none
none	HY08M06u_x_at	1.78	2.44	none	none
none	HF07C04r_at	1.13	2.45	none	none
none	Contig18378_s_at	-1.65	2.46	none	none
none	HY10A09u_at	-1.21	2.46	none	none
none	Contig25213_at	1.05	2.47	none	none
none	HVSMEa0006K18r2_at	1.16	2.47	none	none
none	HVSMEi0011P17r2_at	-1.25	2.48	none	none
none	Contig11919_at	-1.01	2.49	none	none
none	Contig17087_at	-1.13	2.5	none	none
none	S0000200070D03F1_x_at	-1.15	2.5	none	none
none	Contig17832_at	-1	2.51	none	none
none	Contig18240_at	-1.06	2.52	none	none
none	HZ50B24r_s_at	-1.18	2.52	none	none
none	Contig19463_at	1.29	2.53	none	none
none	HA03H21u_x_at	1.15	2.53	none	none
none	HU05O16u_at	-1.12	2.53	none	none

none	HVSMEm0008H21r2_at	1.02	2.54	none	none
none	rbaal37j13_at	-1.21	2.55	none	none
none	Contig8573_at	1.09	2.56	none	none
none	HK04G23r_s_at	1.45	2.56	none	none
none	HT09C05u_x_at	-1.26	2.56	none	none
none	HF24I04r_at	1.04	2.57	none	none
none	HO06H02S_at	1.04	2.57	none	none
none	HVSMEh0084N15r2_s_at	-1.81	2.57	none	none
none	Contig4339_at	1.08	2.58	none	none
none	HO02O08S_at	-1.58	2.58	none	none
none	HVSMEa0009A11r2_at	1.01	2.6	none	none
none	HVSMEa0016C11r2_x_at	1.02	2.6	none	none
none	Contig11541_at	1.28	2.62	none	none
none	EBma04_SQ002_L23_s_at	-1.05	2.63	none	none
none	HC106A04_T3_at	-1.09	2.63	none	none
none	HVSMEk0003F18r2_at	-1.36	2.63	none	none
none	rbaal12h01_at	1.95	2.63	none	none
none	HB21C01r_at	1.25	2.64	none	none
none	HD07K11r_at	1.14	2.65	none	none
none	Contig10140_s_at	1.85	2.66	none	none
none	Contig6449_at	1.24	2.66	none	none
none	rbags12d05_x_at	-1.33	2.66	none	none
none	Contig22429_at	1.03	2.67	none	none
none	HF03K12r_x_at	-1.15	2.67	none	none
none	HVSMEb0001J09r2_at	-1.11	2.68	none	none
none	HVSMEh0094K14f_s_at	1.58	2.68	none	none
none	Contig18959_at	1.58	2.7	none	none
none	Contig7698_at	1.17	2.7	none	none
none	HVSMEf0001G07r2_at	-1.08	2.71	none	none
none	HI04I05u_at	1.92	2.72	none	none
none	HV_CEa0004E24r2_at	1.11	2.74	none	none
none	Contig12631_at	-1.29	2.76	none	none
none	HVSMEb0002B09f_at	1.17	2.76	none	none
none	HVSMEl0020C07r2_x_at	-1.52	2.76	none	none
none	HK05K17r_at	-1.32	2.77	none	none
none	HVSMEg0015I17f_at	1.68	2.77	none	none
none	HU05P08r_at	-1.07	2.78	none	none
none	EBro08_SQ006_E16_at	-1.32	2.79	none	none
none	HVSMEg0008C16r2_s_at	1.15	2.79	none	none
none	Contig25703_at	1.03	2.82	none	none
none	Contig14105_at	-1.05	2.83	none	none
none	HVSMEi0002I06r2_at	-1.01	2.85	none	none
none	Contig24763_at	1.16	2.86	none	none
none	HVSMEl0020G17r2_at	1.89	2.86	none	none
none	Contig16496_at	-1.43	2.87	none	none
none	HC107D03_SK_at	1.02	2.9	none	none
none	EBma04_SQ004_G11_at	-1.08	2.96	none	none
none	HB26G03r_at	1.04	2.96	none	none
none	Contig9234_at	1.01	2.97	none	none
none	HVSMEi0013P04r2_at	1.08	2.97	none	none
none	EBma03_SQ002_M18_at	-1.21	2.98	none	none
none	HVSMEb0006O21r2_at	1.09	2.98	none	none
none	HVSMEf0005G15r2_x_at	1.32	2.98	none	none
none	HB23K10r_at	-1.34	3.02	none	none
none	HM07I17r_at	-1.28	3.02	none	none
none	HW04M05u_x_at	1.94	3.02	none	none
none	HVSMEb0001F21r2_x_at	-1.41	3.04	none	none
none	Contig20688_at	-1.25	3.05	none	none
none	HB32A22r_x_at	1.24	3.05	none	none
none	Contig12609_at	1.16	3.06	none	none
none	Contig5073_x_at	-1.01	3.06	none	none
none	Contig26149_at	1.15	3.08	none	none
none	HVSMEa0005M17r2_at	1.06	3.1	none	none

none	HVSMEn0009E17f_at	-1.04	3.12	none	none
none	HVSMEn0016M22r2_at	1.04	3.12	none	none
none	Contig16058_at	-1.08	3.14	none	none
none	EBem05_SQ003_J11_at	1.18	3.15	none	none
none	HVSMEa0015B19r2_x_at	-1.31	3.17	none	none
none	EBpi03_SQ003_K06_at	-1.24	3.2	none	none
none	HO02O02S_at	-1.21	3.2	none	none
none	HVSMEb0001J09r2_x_at	1.2	3.2	none	none
none	HVSMEn0024P16f_x_at	1.45	3.23	none	none
none	HB32A22r_at	1.41	3.24	none	none
none	EBma05_SQ002_C09_at	-1.25	3.26	none	none
none	Contig19296_at	-1.49	3.27	none	none
none	HV05C06u_x_at	-1.02	3.28	none	none
none	HW06N09u_x_at	1.07	3.29	none	none
none	HY08G17u_at	1.11	3.29	none	none
none	Contig11003_at	-1.08	3.31	none	none
none	HVSMEa0003B16r2_x_at	-1.46	3.32	none	none
none	HVSMEn0010A20f_at	-1.1	3.34	none	none
none	EBed01_SQ002_E13_at	-1.76	3.35	none	none
none	HVSMEa0006A05r2_x_at	-1.37	3.36	none	none
none	HC02E04_T3_at	1.22	3.37	none	none
none	EBma03_SQ001_D18_at	-1.2	3.38	none	none
none	HA03H11u_x_at	-1.08	3.4	none	none
none	EBro02_SQ006_G13_at	-1.09	3.42	none	none
none	HZ62C10r_at	-1.06	3.43	none	none
none	Contig14685_at	1.21	3.44	none	none
none	HB04I10r_x_at	-1.11	3.45	none	none
none	HV14M05r_at	-1.51	3.48	none	none
none	HVSMEc0001E08f2_at	-1.34	3.52	none	none
none	HVSMEl0002D12r2_x_at	1.01	3.52	none	none
none	HT06I11u_at	-1.02	3.53	none	none
none	HM03H10u_at	1.27	3.54	none	none
none	HVSMEn0020A01f_at	1.02	3.65	none	none
none	HT12P14u_at	-1.39	3.66	none	none
none	Contig14088_s_at	1.5	3.67	none	none
none	Contig2082_x_at	1.21	3.7	none	none
none	HVSMEl0010I15r2_s_at	1.97	3.7	none	none
none	rbaal17b01_s_at	-1.2	3.72	none	none
none	HY06G11u_at	1.11	3.79	none	none
none	Contig14088_x_at	1.28	3.83	none	none
none	Contig1502_at	1.54	3.85	none	none
none	HU05O20u_x_at	-1.23	3.87	none	none
none	Contig26496_at	-1.69	3.89	none	none
none	HK05N20r_at	-1.13	3.92	none	none
none	HM08L19r_at	-1.07	3.92	none	none
none	HVSMEk0017B16r2_at	-1.09	3.96	none	none
none	HVSMEh0081C04r2_s_at	-1.42	3.99	none	none
none	HT07L08u_at	1.45	4.02	none	none
none	Contig24783_at	1.14	4.05	none	none
none	HB09B20T_at	1.07	4.05	none	none
none	HV10G07u_at	-1.71	4.05	none	none
none	HVSMEl0025J01f_at	1.06	4.06	none	none
none	EBro01_SQ005_O17_at	-1.15	4.21	none	none
none	HW06L12u_at	-1.27	4.21	none	none
none	HB04I10r_at	1.31	4.22	none	none
none	HVSMEg0015H12r2_at	1.65	4.27	none	none
none	Contig5270_x_at	-1.02	4.28	none	none
none	HVSMEl0011N19r2_x_at	-1.83	4.34	none	none
none	Contig6741_at	-1.41	4.38	none	none
none	HVSMEn0005A08f_at	-1.9	4.42	none	none
none	HF21N04r_at	-1.41	4.49	none	none
none	HVSMEl0008K15r2_at	1.1	4.52	none	none
none	HVSMEm0022H22r2_at	-1.3	4.54	none	none

none	HV_CEb0017E04r2_at	1.16	4.59	none	none
none	HV_CEa0014A11r2_at	-1.82	4.6	none	none
none	HVSMEm0012M15r2_at	-1.25	4.61	none	none
none	HVSMEa0006K18r2_x_at	-1.29	4.75	none	none
none	Contig18830_s_at	1.05	4.76	none	none
none	Contig5073_at	-1.44	4.87	none	none
none	HVSMEl0007J20r2_at	1.06	5.14	none	none
none	Mla13_div5_x_at	1.43	5.19	none	none
none	HF16J13r_at	-1.11	5.34	none	none
none	HVSMEl0006C17r2_x_at	-1.09	5.39	none	none
none	Contig22670_at	-1.15	5.5	none	none
none	Contig3187_s_at	-1.71	5.53	none	none
none	HVSMEf0005G15r2_at	-1.04	5.57	none	none
none	HVSMEc0010M05r2_at	-1.17	5.59	none	none
none	EBan01_SQ004_G02_at	-1.83	5.9	none	none
none	HVSMEg0016O05r2_at	-1.72	5.9	none	none
none	Contig22071_at	1.3	6.05	none	none
none	HM10C10r_at	1.09	6.05	none	none
none	S0001000056F16F1_at	1.25	6.08	none	none
none	EBed01_SQ002_N21_at	1.51	7.43	none	none
none	HW06A17u_x_at	-1.12	7.61	none	none
none	Contig26462_at	-1.25	7.72	none	none
none	HD14K24r_at	-1.71	7.87	none	none
none	HW09C21u_x_at	-1.99	8.01	none	none
none	HS04H24r_at	-1.32	8.09	none	none
none	HD09M17r_at	1.17	9.38	none	none
none	HA22H13r_at	-1.35	9.66	none	none
none	HM01F03w_at	1.38	10.04	none	none
none	Contig2809_at	-1.6	10.12	none	none
none	Contig10584_at	-1.15	10.76	none	none
none	HVSMEm0001B20r2_at	1.34	11.95	none	none
none	rbags18e07_at	1.34	15.58	none	none
none	HVSMEf0003F23r2_at	0	21.64	none	none
none	HY10H17u_at	1.05	36.68	none	none
none	HW02A11u_s_at	-1.73	169.15	none	none
none	HS05F03r_s_at	1.51	193.12	none	none

* The fold change represents the mean ratio of gene expression in leaves of the two genotypes exposed to 5 μM Cd for 15 d over those in the control. Genes were considered up-regulated and down-regulated if the induction ratio was >2.0 and <-2.0, respectively.
