# Supplementary material for: Genome-wide transcriptome and functional analysis of two contrasting genotypes reveals key genes for cadmium tolerance in barley
Source: BMC Genomics. 2014 Jul 19;15(1):611. doi: 10.1186/1471-2164-15-611 (PMC4117959; doi:10.1186/1471-2164-15-611)
Supplement: Supplementary file 12 — Additional file 12: Figure S2: Functional categorisation and differential expression of Cd stress-regulated genes in barley leaves. (PDF 17 KB) [file 12864_2014_6304_MOESM12_ESM.pdf]

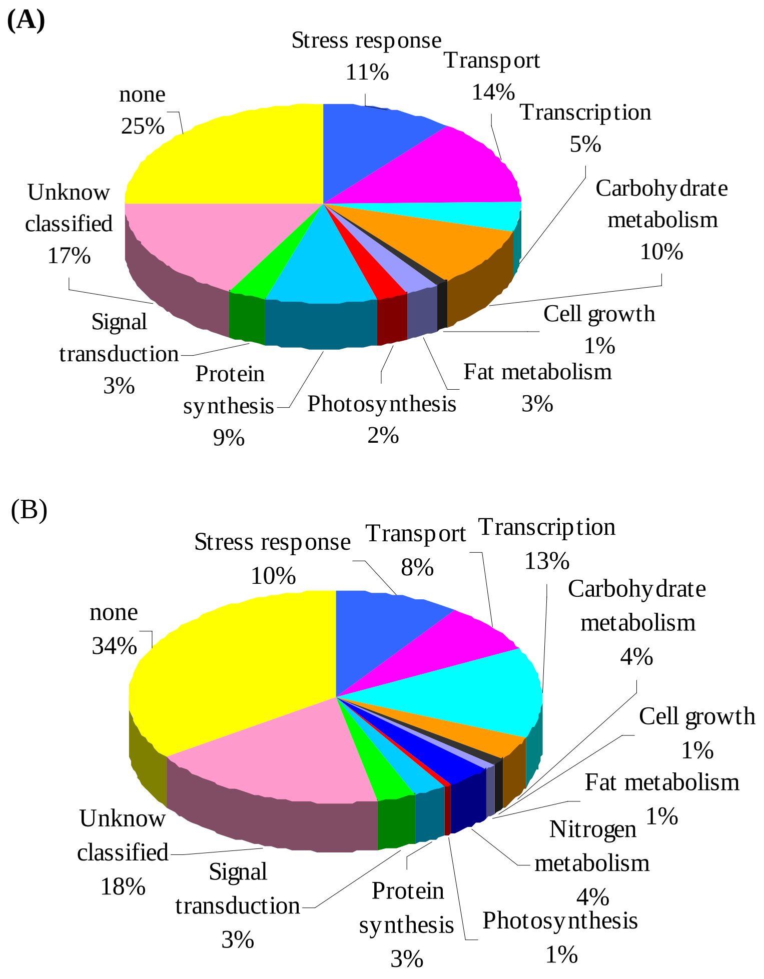

Additional File 2: Figure S2 Functional categorisation and differential expression of Cd stress-regulated genes in barley leaves. Functional categorisation was performed according to the agriGO methods. Pie charts show the distribution of different functional genes after exposing the plants to 5 μM Cd for 15 d. (A) up-regulated in Weisuobuzhi; (B) down-regulated in Dong 17.
