# Supplementary material for: Genome-wide transcriptome and functional analysis of two contrasting genotypes reveals key genes for cadmium tolerance in barley
Source: BMC Genomics. 2014 Jul 19;15(1):611. doi: 10.1186/1471-2164-15-611 (PMC4117959; doi:10.1186/1471-2164-15-611)
Supplement: Supplementary file 13 — Additional file 13: Figure S3: Integrated schematic diagram of the mechanisms involved in vacuolar compartmentalization of Cd in barley leaves. (PDF 63 KB) [file 12864_2014_6304_MOESM13_ESM.pdf]

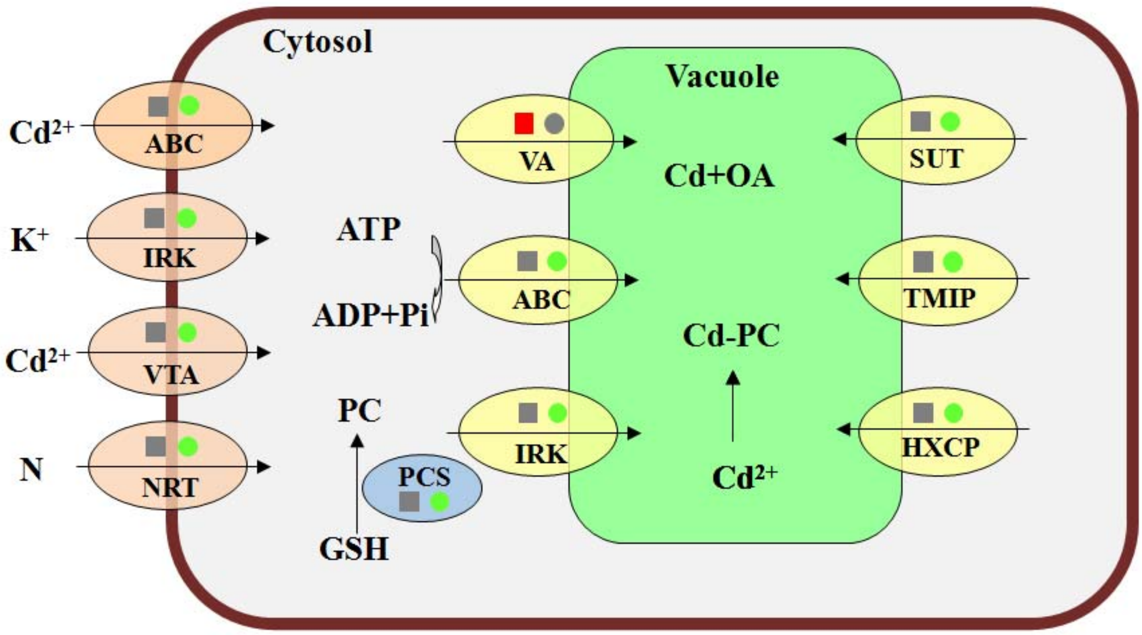

Additional File 3: Figure S3 Integrated schematic diagram of the mechanisms involved in vacuolar compartmentalization of Cd in barley leaves. Genes labelled with red, grey and green squares (Weisuobuzhi) circles (Dong17) are up-regulated, not changed and down-regulated by 5 µM Cd treatment, respectively. ABC, ABC transporter family protein; HXCP, Hexose carrier protein; IRK, Inwardly rectifying potassium channel; NRT, Nitrate and Nitrite transporter; OA, organic acids; PC, Phytochelatin; PCS, Phytochelatin synthetase; SUT, Sugar transporter; TMIP, Tonoplast membrane integral protein; VA, Vacuolar ATP synthase; VTA, Vesicle transfer ATPase.
