# Supplementary material for: Genome-wide transcriptome and functional analysis of two contrasting genotypes reveals key genes for cadmium tolerance in barley
Source: BMC Genomics. 2014 Jul 19;15(1):611. doi: 10.1186/1471-2164-15-611 (PMC4117959; doi:10.1186/1471-2164-15-611)
Supplement: Supplementary file 14 — Additional file 14: Table S11: Links between the novel Cd-responsive genes in barley leaves from this study and Cd-responsive miRNAs and their putative targets from the literature. (PDF 14 KB) [file 12864_2014_6304_MOESM14_ESM.pdf]

Additional File 14: Table S11 Links between the novel Cd-responsive genes in barley leaves from this study and Cd-responsive miRNAs and their putative targets from the literature.

Gene	Fold change (Cd *vs* control)		Changes of miRNA	Target	Reference
	Weisuobuzhi	Dong17			
Leucine-rich repeat transmembrane protein kinase	2.92	2.15	miR390 (-)	Leucine-rich repeat receptor-like protein kinase	Ding et al. (2011)
Apoplastic invertase	2.82	1.83	miR167 (-)	Invertase-like protein	Zhou et al. (2012)
Cytochrome P450	2.64	1.94	Osa-miR604 (-)	Cytochrome P450 family proteins	Huang et al. (2009)
Strictosidine synthase-related protein	2.31	2.04	Osa-miR606 (-)	Strictosidine synthas	Huang et al. (2009)
Glutathione transferase	2.23	2.65	miR156 (-)	Glutathione s-transferase 5	Xu et al. (2013)
AP2 domain transcriptional regulator	1.93	2.77	miR169 (-)	AP2 domain transcription factor	Xu et al. (2013)
Phytochelatin synthetase-like protein	1.15	-2.14	miR393 (+)	Phytochelatin synthetase 1	Xu et al. (2013)
DNA-binding protein 2	1.11	-3.2	MiR393 (+)	Similarity to DNA-binding protein	Zhou et al. (2012)
Serine/threonine kinases	1.06	2.07	miR156 (-)	Serine/threonine protein kinases Nek3	Zhou et al. (2012)
bZIP transcription factor	-1.02	2.38	miR166i/e/m/k/g (-)	HD Zip transcription factor	Ding et al. (2011)
Calcium binding EF-hand protein	-1.18	2.68	miR1432 (-)	EF-hand proteins	Ding et al. (2011)
Leucine zipper protein	-1.19	2.25	miR166 (-)	Homeodomain- leucine zipper protein	Xu et al. (2013)
Heat shock protein	-1.35	-2.74	miR396 (+)	Heat shock protein 90	Xu et al. (2013)

+/-, up-regulated/down-regulated in the corresponding reference

Ding YF, Chen Z, Zhu C: **Microarray-based analysis of cadmium-responsive microRNAs in rice (*Oryza sativa*).** *J Exp Bot* 2011, **60:**3563-3573.

Huang SQ, Peng J, Qiu CX, Yang ZM: **Heavy metal-regulated new microRNAs from rice.** *J Inorg Biochem* 2009, **103:**282-287.

Xu L, Wang Y, Zhai LL, Xu YY, Wang LJ, Zhu XW, Gong YQ, Yu RG, Limera C, Liu LW: **Genome-wide identification and characterization of cadmium-responsive microRNAs and their target genes in radish (*Raphanus sativus* L.) roots.** *J Exp Bot* 2013, **64:**4271-4287.

Zhou ZS, Song JB, Yang ZM: **Genome-wide identification of *Brassica napus* microRNAs and their targets in response to cadmium.** *J Exp Bot* 2012, **63:**4597-4613.
